# Conserved S-Layer-Associated Proteins Revealed by Exoproteomic Survey of S-Layer-Forming Lactobacilli

**DOI:** 10.1128/AEM.01968-15

**Published:** 2015-12-22

**Authors:** Brant R. Johnson, Jeffrey Hymes, Rosemary Sanozky-Dawes, Emily DeCrescenzo Henriksen, Rodolphe Barrangou, Todd R. Klaenhammer

**Affiliations:** a Graduate Program in Microbiology, College of Agriculture and Life Sciences, North Carolina State University, Raleigh, North Carolina, USA; b Department of Food, Bioprocessing, and Nutrition Sciences, North Carolina State University, Raleigh, North Carolina, USA

## Abstract

The Lactobacillus acidophilus homology group comprises Gram-positive species that include L. acidophilus, L. helveticus, L. crispatus, L. amylovorus, L. gallinarum, L. delbrueckii subsp. bulgaricus, L. gasseri, and L. johnsonii. While these bacteria are closely related, they have varied ecological lifestyles as dairy and food fermenters, allochthonous probiotics, or autochthonous commensals of the host gastrointestinal tract. Bacterial cell surface components play a critical role in the molecular dialogue between bacteria and interaction signaling with the intestinal mucosa. Notably, the L. acidophilus complex is distinguished in two clades by the presence or absence of S-layers, which are semiporous crystalline arrays of self-assembling proteinaceous subunits found as the outermost layer of the bacterial cell wall. In this study, S-layer-associated proteins (SLAPs) in the exoproteomes of various S-layer-forming Lactobacillus species were proteomically identified, genomically compared, and transcriptionally analyzed. Four gene regions encoding six putative SLAPs were conserved in the S-layer-forming Lactobacillus species but not identified in the extracts of the closely related progenitor, L. delbrueckii subsp. bulgaricus, which does not produce an S-layer. Therefore, the presence or absence of an S-layer has a clear impact on the exoproteomic composition of Lactobacillus species. This proteomic complexity and differences in the cell surface properties between S-layer- and non-S-layer-forming lactobacilli reveal the potential for SLAPs to mediate intimate probiotic interactions and signaling with the host intestinal mucosa.

## INTRODUCTION

Bacterial cell surface proteins play a critical role in the molecular dialogue between bacteria and their interaction with the host. For beneficial microbes, such as probiotics, these proteins mediate health-promoting functions through gastrointestinal adhesion, competitive exclusion of pathogens, enhancement of intestinal barrier function, and activation of gut mucosal immunity (1, 2). Probiotics are defined by the FAO/WHO as “live microorganisms that, when administered in adequate amounts, confer a health benefit on the host” (3). Some beneficial actions of these organisms are strain specific and can be harnessed to treat or reduce the risk of multiple maladies, including acute infectious diarrhea, irritable bowel syndrome, vaginal infections, ulcerative colitis, lactose maldigestion, and necrotizing enterocolitis (4). In fact, the efficacy of probiotic treatment depends largely on the various cell surface components that mediate this specificity (5). Therefore, the characterization of effector cell surface ligands and their health-promoting interactions with the host is of increasing scientific and medical interest.

Some of the most prevalent and well-studied probiotics are lactobacilli, many of which are members of the Lactobacillus acidophilus homology group (6). The L. acidophilus group is a clade of homologous Gram-positive Lactobacillus species that includes L. acidophilus, L. helveticus, L. crispatus, L. amylovorus, L. gallinarum, L. delbrueckii subsp. bulgaricus, L. gasseri, and L. johnsonii (7–11). Although these bacteria are closely related phylogenetically, they have varied ecological lifestyles ranging from dairy and food fermentations to allochthonous probiotics or autochthonous commensals of the host gastrointestinal and urogenital tracts. Biochemically, they are obligately homofermentative; they almost exclusively ferment sugar (>85%) to lactate via the Embden-Meyerhof-Parnas pathway. Early taxonomic descriptions were based on the metabolic end products of fermentation, resulting in a seemingly indistinguishable group of microbes, which were all called L. acidophilus (10). However, DNA-DNA hybridization studies revealed the heterogeneity in the group (11, 12). Since then, genome sequencing and comparative genomic analyses have clearly established and solidified the current description of the L. acidophilus group (13, 14). Notably, these closely related strains can be dichotomized based on their ability to create surface (S)-layer protein arrays as the outermost constituent of the cell wall (15).

Bacterial S-layers are semiporous proteinaceous crystalline arrays composed of self-assembling (glyco)protein subunits called S-layer proteins (SLPs) (15). They can be found in both Gram-positive and Gram-negative bacteria and species of Archaea but are not ubiquitous in all microorganisms. When present, S-layers form two-dimensional lattices on the outermost layer of the cell, which are tethered through noncovalent interactions with the cell wall (15). S-layers from various species of the L. acidophilus homology group have been characterized for their roles in intestinal adhesion, competitive exclusion of pathogens, and immunomodulation of the gastrointestinal mucosa. *In vitro* studies using intestinal epithelial cell lines suggest that the S-layer is a major factor in intestinal adhesion for L. acidophilus (16, 17), L. crispatus (18–20), L. helveticus (21), and L. amylovorus (22). In fact, this adhesion has been shown to competitively exclude enteropathogenic bacteria by both L. crispatus (23) and L. helveticus (24, 25). Compelling studies have begun to reveal the mechanisms of gastrointestinal immunomodulation. For example, SlpA, the primary constituent of the S-layer in L. acidophilus NCFM, was found to bind to dendritic cell (DC) orthologous C-type lectin receptors (CLR), DC-specific intercellular adhesion molecule 3 (ICAM-3)-grabbing nonintegrin (DC-SIGN) (26), and a specific intracellular adhesion molecule-3-grabbing nonintegrin homolog-related 3 (SIGNR-3) (27). This SlpA-CLR interaction exerts regulatory signals, which have been reported to mitigate inflammatory disease states and promote the maintenance of healthy intestinal barrier function (27). Similar experiments have aimed to elucidate the roles of the S-layer in modulating gastrointestinal immunity for L. crispatus (28), L. helveticus (29), and L. amylovorus (22).

The S-layer-forming species of the L. acidophilus homology group form S-layers composed of a dominant protein constituent, SlpA/Slp1 (∼46 kDa), and the minor constituents SlpB/Slp2 (∼47 kDa) and SlpX (∼51 kDa) (30). Recent evidence, however, suggests that the S-layer may not be as monomorphic as previously proposed. In L. acidophilus NCFM, proteomic analysis revealed the presence of 37 noncovalently bound extracellular S-layer-associated proteins (SLAPs), 23 of which are putative/uncharacterized proteins of unknown function (31). In this study, the noncovalent exoproteomes of various S-layer- and non-S-layer-forming Lactobacillus strains were proteomically identified, genomically compared, and transcriptionally analyzed. These data reveal both the conservation and variability of SLAPs across lactobacilli and their potential to mediate intimate interactions with the intestinal mucosa.

## MATERIALS AND METHODS

### Bacterial strains and growth conditions.

The bacterial strains used in this study are reported in Table 1. Lactobacillus strains were propagated statically at 37°C under ambient atmospheric conditions in de Man-Rogosa-Sharpe (MRS) broth (Difco Laboratories, Inc., Detroit, MI).

**TABLE 1:** Strains used in this study

Organism (strain)^*a*^	Study designation	Source^*b*^	Origin	S-layer	Reference
L. acidophilus (NCFM)	NCK56		Human intestinal isolate	**+**	47
L. helveticus (1846)	NCK230	NCDO	Dairy isolate	+	48
**L. helveticus** (481-C)	NCK246	NCDO	Dairy isolate	+	49
L. helveticus	NCK338	NCDO	Dairy isolate	+	50
**L. helveticus** (CNRZ32)	NCK936	CNRZ	Industrial cheese starter culture	+	51
**L. helveticus** (ATCC 15009)	NCK1088	ATCC	Dairy isolate	+	52
**L. crispatus** (ATCC 33820)	NCK777	ATCC	Human isolate	+	53, 54
**L. crispatus**	NCK953		Chicken isolate	+	
**L. crispatus** (CZ6)	NCK1351		Human endoscopy isolate	+	55
**L. amylovorus** (ATCC 33620)	NCK776	ATCC	Cattle feces	+	56, 57
L. gallinarum (ATCC 33199)	NCK778	ATCC	Chicken isolate	+	58
L. gallinarum	NCK1560		Chicken isolate	+	
**L. delbrueckii subsp. bulgaricus**	NCK1561		Dairy isolate	−	
L. gasseri (ATCC 33323)	NCK334	ATCC	Human isolate	−	59
L. johnsonii (ATCC 33200)	NCK779	ATCC	Human isolate	−	58
L. reuteri (ATCC 23272)^*c*^	NCK702	ATCC	Human feces	−	11
**L. casei** (ATCC 393)^*c*^	NCK125	ATCC	Dairy isolate	−	60

a Proteins from organisms indicated in bold were proteomically identified using LC-MS/MS.

b NCDO, National Collection of Dairy Organisms; ATCC, American Type Culture Collection; CNRZ, Centre National de Recherches Zootechniques.

c Species outside the L. acidophilus homology group.

### DiversiLab analysis of strains.

L. crispatus and L. helveticus strains were typed using the repetitive extragenic palindromic-PCR (Rep-PCR)-based DiversiLab typing system (bioMérieux, Durham, NC). DNA from the Lactobacillus strains was extracted using a Mo Bio UltraClean microbial DNA isolation kit (Mo Bio, Carlsbad, CA) and quantified using a NanoDrop 1000 spectrophotometer (Thermo Scientific, Waltham, MA). The DNA was then normalized to 20 ng μl^−1^ with UltraPure distilled water (Invitrogen, Carlsbad, CA). Rep-PCR was performed in preparation for typing using the Lactobacillus DiversiLab kit (bioMérieux). DNA amplification was performed in a Bio-Rad MyCycler thermal cycler (Bio-Rad, Hercules, CA), programmed for 2 min at 94°C (initial denaturation) and 35 cycles of 30 s at 94°C (denaturation), 30 s at 55°C (annealing), and 90 s at 70°C (extension), followed by a final extension cycle of 3 min at 70°C using AmpliTaq DNA polymerase from Applied Biosystems (Carlsbad, CA). The reaction mixture was pipetted into the DiversiLab system chip along with the DiversiLab DNA reagents and supplies (bioMérieux), according to the manufacturer's protocol. The chip samples were analyzed using the DiversiLab software version 3.4, and the similarity of the strains was determined by comparing the resulting electropherogram/bar codes.

### Extraction of extracellular noncovalently bound cell surface proteins.

Noncovalently bound cell surface proteins, including S-layer proteins and S-layer-associated proteins, were extracted from the Lactobacillus strains using LiCl denaturing salt, as described previously (31). Briefly, cells were grown in 200 ml of MRS broth to stationary phase (16 h), centrifuged at 2,236 × *g* for 10 min (4°C), and washed twice with 25 ml of cold phosphate-buffered saline (PBS) (Gibco) (pH 7.4). The cells were agitated for 15 min at 4°C following the addition of 5 M LiCl (Fisher Scientific). Supernatants containing SLPs and SLAPs were harvested via centrifugation at 8,994 × *g* for 10 min (4°C), transferred to a 6,000- to 8,000-kDa Spectra/Por molecular porous membrane (Spectrum Laboratories), and dialyzed against cold distilled water for 24 h. The precipitate was harvested at 20,000 × *g* for 30 min and agitated for a second time with 1 M LiCl at 4°C for 15 min to disassociate the SLAPs from the SLPs. The suspension was then centrifuged at 20,000 × *g* for 10 min, and the SLAP supernatants were separated from the SLP pellet, transferred to the 6,000- to 8,000-kDa Spectra/Por molecular porous membrane, and dialyzed against cold distilled water for 24 h. Finally, the precipitate was harvested via centrifugation at 20,000 × *g* for 30 min to pellet the SLAPs. Both SLP and SLAP pellets were resuspended in 10% (wt/vol) SDS (Fisher). Proteins were quantified via a bicinchoninic acid assay kit (Thermo Scientific) and visualized via SDS-PAGE using precast 4% to 20% Precise Tris-HEPES protein gels (Thermo Scientific). The gels were stained using AcquaStain (Bulldog Bio), according to the manufacturer's instructions. SLAP extractions were performed with two biological replicates for each strain and visualized through SDS-PAGE to confirm that the resultant banding patterns were reproducible.

### Proteomic identification and analysis.

SLAPs extracted from the various Lactobacillus species were identified using liquid chromatography-tandem mass spectrometry (LC-MS/MS) from the Genome Center Proteomics Core at the University of California, Davis, CA, as described previously (31). Proteomic screenings were performed once per strain and used as a tool for selecting candidate SLAPs within each strain. Tandem mass spectra were extracted and the charge state deconvoluted using MM File Conversion version 3. All MS/MS samples were analyzed using X! Tandem (Tornado version; The GPM [www.thegpm.org/]). UniProt searches were performed using proteome databases for the respective proteins isolated from L. acidophilus NCFM, L. helveticus CNRZ32, L. crispatus ST1, and L. amylovorus GRL1112. X! Tandem was searched with a fragment ion mass tolerance and parent ion tolerance of 20 ppm. The iodoacetamide derivative of cysteine was specified in X! Tandem as a fixed modification. The deamination of asparagine and glutamine, oxidation of methionine and tryptophan, sulfonation of methionine, tryptophan oxidation to formylkynurenine of tryptophan, and acetylation of the N terminus were specified in X! Tandem as variable modifications. Scaffold (version Scaffold_3.6.1; Proteome Software) was used to validate MS/MS-based peptide and protein identifications. Peptide identifications were accepted if they exceeded specific database search engine thresholds. X! Tandem identifications required scores of >1.2 with a mass accuracy of 5 ppm. Protein identifications were accepted if they contained at least two identified peptides. Using the parameters described above, the false-discovery rate was calculated to be 1.1% at the protein level and 0% at the peptide level. Proteins that contained similar peptides and that could not be differentiated based on MS/MS analysis alone were grouped to satisfy the principles of parsimony. For this study, only proteins with unique spectral counts of >20 were considered significant. For all analyses, total spectral counts were utilized as a semiquantitative indicator of protein abundance (32). Two-way clustering of total spectral counts was performed using JMP Genomics (version 5; SAS). Protein domains were identified for analysis using the Pfam protein family database (33).

### Genomic *in silico* analyses.

Genomic analysis was performed on genomes curated from the genome library of the National Center for Biotechnology Information (NCBI [http://www.ncbi.nlm.nih.gov/genome/]), including L. acidophilus NCFM (GenBank accession no. NC_006814.3), L. helveticus CNRZ32 (GenBank accession no. NC_021744.1), L. amylovorus GRL1112 (GenBank accession no. NC_014724.1), L. crispatus ST1 (GenBank accession no. NC_014106.1), L. delbrueckii subsp. bulgaricus ATCC 11842 (GenBank accession no. NC_008054.1), and L. casei ATCC 334 (GenBank accession no. NC_008526.1). Identified genes were compared using the BLASTn and BLASTp features of NCBI (http://blast.ncbi.nlm.nih.gov/Blast.cgi). SignalP 4.1 was used to predict the signal peptidase cleavage site of each identified protein (34). Genomes were uploaded to Geneious 8.0.5 (35) for comparative genomic and promoter analyses of the identified SLAP genes. The genetic context of SLAP genes was examined using the chromosomal graphical interface in Geneious 8.0.5. *In silico* promoter elements were identified in the upstream intergenic regions of SLAP genes using PromoterWise (http://www.ebi.ac.uk/Tools/psa/promoterwise/). To identify conserved promoter elements between the various SLAP genes, genome-wide sequence motifs of the putative −10 and −35 regions were scanned against the four S-layer-forming genomes using Geneious 8.0.5, with a variable spacer length of 16 to 23 nucleotides (nt) between the −10 and −35 regions.

### RNA extraction, sequencing, and transcriptional analysis.

Cells were grown to mid-log phase (8 h) and flash-frozen for RNA extraction and sequencing. RNA was extracted using the Zymo Direct-zol RNA MiniPrep kit (Zymo Research, Irvine, CA) and analyzed for quality using an Agilent 2100 Bioanalyzer (Agilent Technologies, Santa Clara, CA). Library preparation and RNA sequencing were performed at the High-Throughput Sequencing and Genotyping Unit of the Roy J. Carver Biotechnology Center, University of Illinois at Urbana-Champaign, IL. For each sample, rRNA was removed with the Ribo-Zero bacterial kit (Illumina, San Diego, CA), followed by library preparation with the TruSeq stranded RNA sample preparation kit (Illumina). Single-read RNA sequencing was performed using an Illumina HiSeq 2500 ultrahigh-throughput sequencing system) with a read length of 180 nt. Raw sequencing reads were assessed for quality using FastQC version 0.11.3 (http://www.bioinformatics.babraham.ac.uk/projects/fastqc/) and processed using Geneious 8.0.5 (35). Briefly, after the adaptor sequences were trimmed, the raw reads were quality trimmed to remove sequence reads with an error probability limit of 0.001 (Phred score, 30) and filtered to remove reads <20 nt. These quality trimmed and filtered sequences were then mapped to the reference genomes of the S-layer-forming Lactobacillus spp. using Bowtie 2 (36), with default settings within Geneious 8.0.5 (35). The sequencing coverage depths were calculated to be 767×, 730×, 727×, and 665× for L. acidophilus NCFM strain NCK56, L. amylovorus ATCC 33620 strain NCK776, L. crispatus ATCC 33820 strain NCK777, and L. helveticus CNRZ32 strain NCK938, respectively. Transcriptional analyses were based on the normalized transcripts per million (TPM) calculation within Geneious 8.0.5 (35).

## RESULTS

### Proteomic identification of noncovalently bound extracellular proteins in S-layer- and non-S-layer-forming lactobacilli.

**FIG 1:**
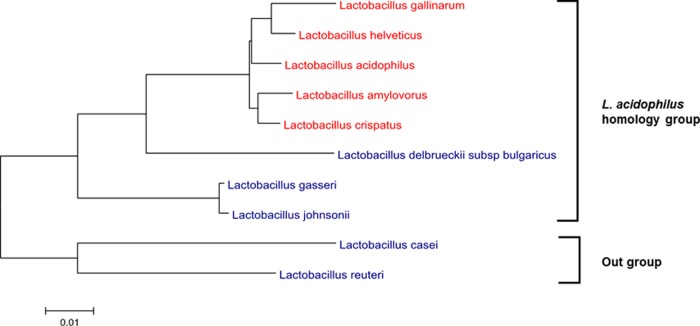
16S rRNA dendrogram of the S-layer-forming (red) and non-S-layer-forming (blue) species of the L. acidophilus homology group. The tree is rooted by the non-S-layer-forming species L. casei and L. reuteri, which are not members of the L. acidophilus homology group.

Based on the previous identification of S-layer-associated proteins (SLAPs) in L. acidophilus NCFM (31), we performed exoproteome screenings on multiple S-layer- and non-S-layer-forming strains of Lactobacillus. Thus, five S-layer- and five non-S-layer-forming Lactobacillus species were analyzed (Fig. 1). Seventeen strains were tested in total, comprising 12 S-layer- and 5 non-S-layer-producing lactobacilli (Table 1). Notably, 15 of the strains are members of the closely related L. acidophilus homology group.

**FIG 2:**
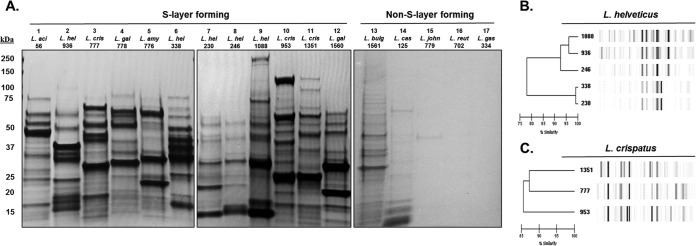
Noncovalently bound exoproteomes were extracted using LiCl and electrophoresed on SDS-PAGE gels. These gels are representative of protein extractions from two biological replicates of each strain. (A) The S-layer-forming strains of the L. acidophilus (*L. aci*) homology group presented a diverse array of proteins in the LiCl extracts, including many anticipated S-layer-associated proteins (SLAPs). In contrast, the non-S-layer-forming species harbored very few proteins in the cell surface extracts. Five strains of L. helveticus (*L. hel*) (B) and three strains of L. crispatus (*L. cris*) (C) were typed using the Rep-PCR-based DiversiLab typing system. *L. gal*, L. gallinarum; *L. amy*, L. amylovorus; *L. bulg*, L. delbrueckii subsp. bulgaricus; *L. cas*, L. casei; *L. john*, L. johnsonii; *L. reut*, L. reuteri; *L. gas*, L. gasseri.

Electrophoresis of SLAP extractions revealed a surprisingly diverse array of protein banding patterns in the S-layer-forming species and a notable absence of proteins in the non-S-layer-forming species (Fig. 2). SLAP extractions were performed on two biological replicates, and the SDS-PAGE banding patterns of the SLAPs extracted from each strain did not differ in the major banding patterns between replicates. Further, the LiCl extract of L. acidophilus demonstrated a banding profile similar to that of the SLAPs identified previously (28) (Fig. 2, lane 1). Proteins from the other S-layer-forming strains, including L. crispatus, L. amylovorus, L. gallinarum, and L. helveticus, were not only distinct from L. acidophilus but also from one another. Moreover, there was also heterogeneity in the protein banding between various strains within each species. In the five L. helveticus strains, there were distinctive differences between the various dairy isolates NCK936, NCK338, NCK230, NCK246, and NCK1088 (Fig. 2A, lanes 2 and 6 to 9). The three L. crispatus strains were also discrete from one another (Fig. 2A, lanes 3, 10, and 11). Rep-PCR-based DiversiLab strain typing was performed on the five L. helveticus and three L. crispatus strains to examine genomic similarities (Fig. 2B and C). The five L. helveticus strains clustered into two groups with >93% and >98% similarity (Fig. 2B), and the L. crispatus strains were >85% similar (Fig. 2C). Remarkably, the L. helveticus strains NCK338 and NCK230, and NCK1088 and NCK936, distinctly varied in terms of the isolated extracellular proteins (Fig. 2A) despite >98% and >95% similarity between the Rep-PCR typing patterns (Fig. 2B). A similar trend was observed among the L. crispatus strains. Thus, there was no correlation between the genotype clustering and the exoproteome profiles revealed by SDS-PAGE.

There were very few proteins isolated from the non-S-layer-forming species of Lactobacillus, as observed in the gel lanes of the SDS-PAGE (Fig. 2, lanes 13 to 17). L. johnsonii and L. gasseri of the L. acidophilus homology group exhibited no discernible proteins in the gel lanes (Fig. 2, lanes 15 and 17). L. delbrueckii subsp. bulgaricus, the non-S-layer-producing strain, which is the most closely related and progenitor to the other S-layer-forming members of the L. acidophilus homology group (Fig. 1), showed only a small number of proteins isolated from the LiCl extract (Fig. 2, lane 13). Distantly related L. casei, devoid of any S-layer, also exhibited few proteins (Fig. 2, lane 14). To identify the electrophoresed proteins, lanes with visible proteins in the gel were sent for proteomic identification (Table 1, in bold).

**FIG 3:**
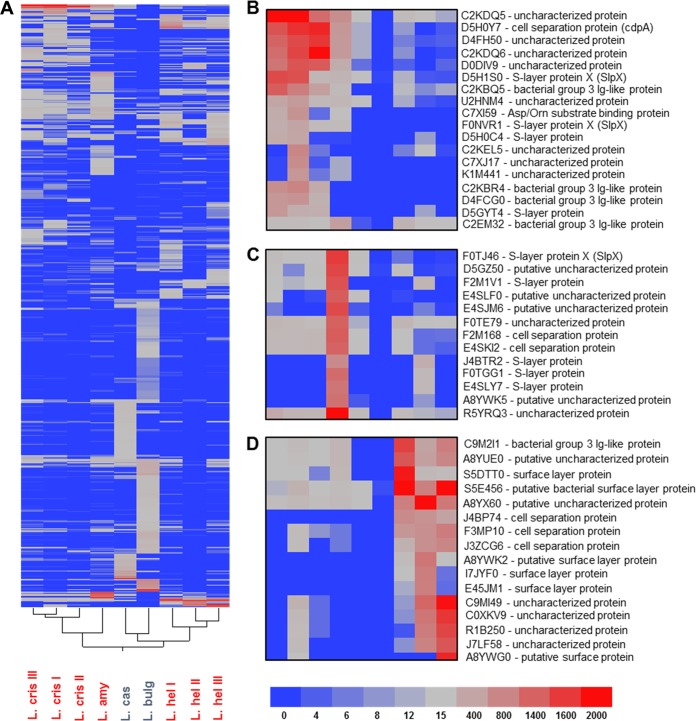
(A) A total of 2,929 proteins were identified from the S-layer-forming strains (red) of L. crispatus, L. amylovorus, and L. helveticus and the non-S-layer-forming strains (blue) of L. delbrueckii subsp. bulgaricus and L. casei. Two-way clustering was performed on the identified proteins based on their similarity between strains and visualized using a red-blue heat map. The colors in the heat map represent the spectral counts of the identified proteins (semiquantitative measure of protein abundance), with red being the most present (400 to 1,000 total spectral counts), gray being somewhat present (12 to 400 total spectral counts), and blue being low or no presence (0 to 12 total spectral counts). Regarding the S-layer-forming strains, there were three main clusters of proteins: SLAPs specific to L. crispatus (B), L. amylovorus (C), and L. helveticus (D). These three clusters have been noted with the corresponding UniProt and protein annotations of the identified proteins.

Of the 12 S-layer-forming strains, seven were selected for proteomic identification, including three L. helveticus strains, three L. crispatus strains, and one L. amylovorus strain (Table 1, underlined). Notably, L. gallinarum was not selected for analysis, as there are no publically available genomes or proteomes published for this species to date. From the five non-S-layer-forming species tested, only L. delbrueckii subsp. bulgaricus and L. casei were selected from proteomic screening, as they were the only non-S-layer-forming species in which proteins were isolated from the SLAP extraction (Table 1, underlined). Proteins were identified from the LiCl extracts of the seven S-layer- and two non-S-layer-forming Lactobacillus species using liquid chromatography-tandem mass spectrometry (see Table S1 in the supplemental material). Two-way clustering was performed based on the total spectral counts of identified proteins and visualized using a two-way clustering heat map (Fig. 3A). The proteins identified in the two non-S-layer-forming strains, L. casei and L. delbrueckii subsp. bulgaricus, are unambiguously distinct from the other seven S-layer-forming strains. Furthermore, almost all of the proteins identified in the non-S-layer-forming strains were predicted intracellular proteins, likely presented extracellularly as the result of cell death occurring at stationary phase. With regard to the S-layer-forming Lactobacillus species, there were three main groupings of proteins identified: SLAPs specific to L. crispatus (Fig. 3B), SLAPs specific to L. amylovorus (Fig. 3C), and SLAPs specific to L. helveticus (Fig. 3D). Surprisingly, although each group had distinctive homologies, the same types of proteins were observed in each group. In fact, these proteins, which included multiple putative uncharacterized proteins, cell surface proteases, and group 3 bacterial Ig-like domain proteins, were the same types of proteins identified as SLAPs in L. acidophilus NCFM (see Table S2 in the supplemental material). Notably, these putative SLAPs were not found in the non-S-layer-producing strains analyzed, which were L. casei and L. delbrueckii subsp. bulgaricus.

### Functional exoproteomic analysis of S-layer- and non-S-layer-forming lactobacilli.

**FIG 4:**
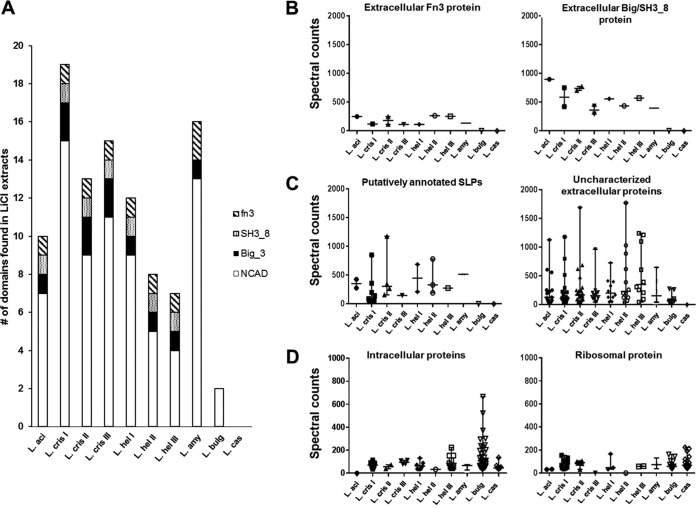
(A) Four protein domains found consistently in the proteins identified within the LiCl extracts: NCAD (white), BIg_3 (black), SH3_8 (dots), and fn3 (diagonal lines). Dot plots were created using the semiquantitative total spectral counts from the identified proteins of each strain. Plotted are the extracellular Fn3 proteins and extracellular BIg_3/SH3_8 proteins (B), putative annotated S-layer proteins and uncharacterized extracellular proteins (C), and intracellular and ribosomal proteins (D). The proteins in panel C contain the NCAD, while the proteins in panel D do not.

After proteomic identification, selected putative SLAPs and noncovalently bound extracellular proteins were functionally analyzed based on predicted protein domains. Four predominant protein domains were found consistently in the S-layer-forming species tested (Fig. 4A), including SLAP (PF03217), Big_3 (PF07523), SH3_8 (PF13457), and fn3 (PF00041). We propose that the SLAP (PF03217) domain, responsible for the noncovalent attachment of SLP and other extracellular proteins in lactobacilli, be redesignated the noncovalent attachment domain (NCAD). This domain designation prevents confusion with the abbreviation for S-layer-associated proteins, SLAPs. Notably, the NCAD was the most abundant protein domain identified in the extracellular fractions tested (Fig. 4A). Other domains associated with bacterial extracellular proteins, including group 3 bacterial Ig-like domains (Big_3), SH3-like domains (SH3_8), and fibronectin type III domains (fn3), were found in the proteomic analysis of the S-layer-forming species but were absent from the non-S-layer-forming species (Fig. 4A). Notably, only two NCAD-containing proteins were identified within the exoproteome of L. delbrueckii subsp. bulgaricus, while none of these domains were identified in the exoproteome of the non-S-layer-forming L. casei.

Identified proteins were functionally categorized based on putative domains and placed into one of six groupings: extracellular fn3 domain proteins and extracellular BIg3/SH3_8 proteins (Fig. 4B), putatively annotated SLPs and uncharacterized extracellular proteins (Fig. 4C), and intracellular proteins and ribosomal proteins (Fig. 4D). The distribution of the proteins within these functional groupings was plotted for each of the strains using the semiquantitative total spectral counts identified through the LC-MS/MS survey (Fig. 4B to D). Group 3 bacterial Ig-like domain proteins, which contain the Big_3 and SH3_8 domains, were only found in the SLAP fractions of the S-layer-forming lactobacilli (Fig. 4B). Similarly, uncharacterized proteins putatively annotated as SLPs and fibronectin-binding proteins were found solely in the S-layer-forming species of Lactobacillus (Fig. 4B and C). There was an increase in both the occurrence and abundance of NCAD-containing uncharacterized extracellular proteins in the SLAP fractions from the S-layer strains compared to the non-S-layer strains (Fig. 4C). Furthermore, there was an increase in the presence of intracellular proteins, including ribosomal proteins, in the non-S-layer strains (Fig. 4D), as measured by total spectral counts. These data reveal a pattern of noncovalently bound proteins identified in S-layer species of Lactobacillus compared to non-S-layer-forming lactobacilli.

### Genomic characterization of genes corresponding to the extracellular S-layer-associated proteins.

**FIG 5:**
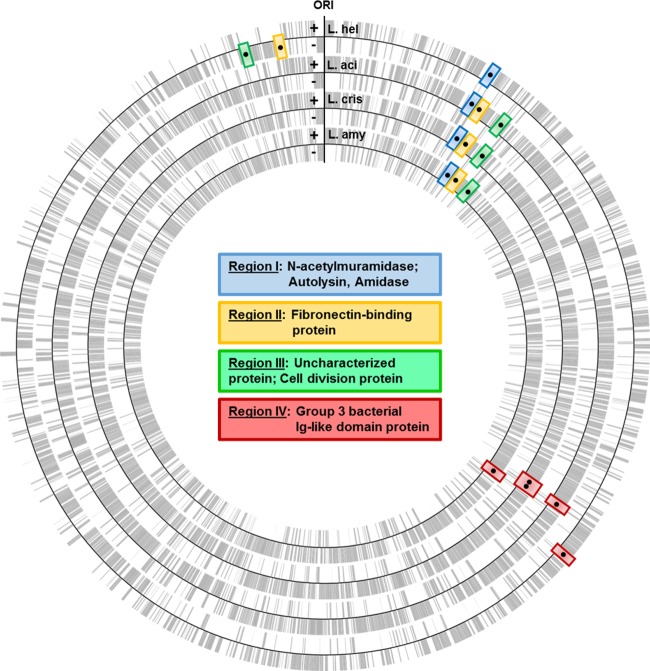
All open reading frames (ORFs) from the positive (+) and negative (−) strands of L. helveticus CNRZ32, L. acidophilus NCFM, L. crispatus ST1, and L. amylovorus GRL1112 were mapped onto circular chromosomes with an annotated origin of replication (Ori). Four conserved SLAP gene regions were identified based on position between strains. Blue, region I; yellow, region II; green, region III; red, region IV.

The putative SLAPs identified in this study, along with the previously identified SLAPs of L. acidophilus NCFM, were curated to the genomes of L. acidophilus NCFM, L. helveticus CNRZ32, L. amylovorus GRL 1112, and L. crispatus ST1 (see Table S2 in the supplemental material). By visualizing the corresponding genes on the four genomes, four conserved genetic regions containing six genes were consistently observed (Fig. 5). Two cell division-related genes, including an *N*-acetylmuramidase and autolysin, are found in region I. Region II is composed of genes encoding fn3 domain-containing fibronectin-binding proteins. Region III also contains two cell division-related genes, including the gene encoding cell division protein A (*cdpA*) (33). Finally, region IV includes genes encoding group 3 bacterial Ig-like proteins, which contain the domains Big_3 and SH3_8. The relative positions of the four gene regions were conserved among the four genomes, with the exception of regions II and III in L. helveticus, which were translocated to the minus strand leading away from the origin of replication (Fig. 5).

**FIG 6:**
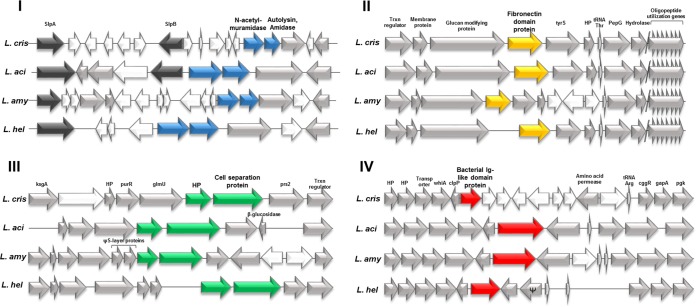
Genomic context of the five SLAP gene regions among the four strains of Lactobacillus: L. crispatus ST1, L. acidophilus NCFM, L. amylovorus GRL1112 and L. helveticus CNRZ32. Arrows represent genes. Gray arrows represent conserved synteny between the four strains, while white arrows represent divergence. Colored arrows represent the SLAP gene regions as follows: blue, region 1; yellow, region II; green, region III; red, region IV. HP, hypothetical protein; Trxn, transcriptional.

In addition, the genetic context of each region was examined within the four strains. Notably, there was synteny observed between the four chromosomal regions of each organism (Fig. 6). Although region I was the least syntenic overall, it is noteworthy that the *N*-acetylmuramidase and autolysin/amidase genes were positioned directly downstream of the genes encoding the primary S-layer protein, *slpA* and *slpB*. Conversely, region II exhibited increased conservation of genetic loci near the SLAP gene encoding a fibronectin-binding protein, including genes for a high-molecular-weight glucan-modifying protein, a tyrosine-tRNA synthetase, and an oligopeptide utilization gene cluster. Region III was also syntenic surrounding the putative SLAP genes, with genes encoding the *pur* operon repressor gene *purR* and the cell division gene *glmU*. Last, region IV containing the gene encoding the putative SLAP with a group 3 bacterial Ig-like domain was directly downstream of the endopeptidase gene, *clpP*, and upstream of the glycolysis genes *gapA* and *pgk*.

### RNA sequencing and transcriptional analysis of the S-layer-forming Lactobacillus species.

**FIG 7:**
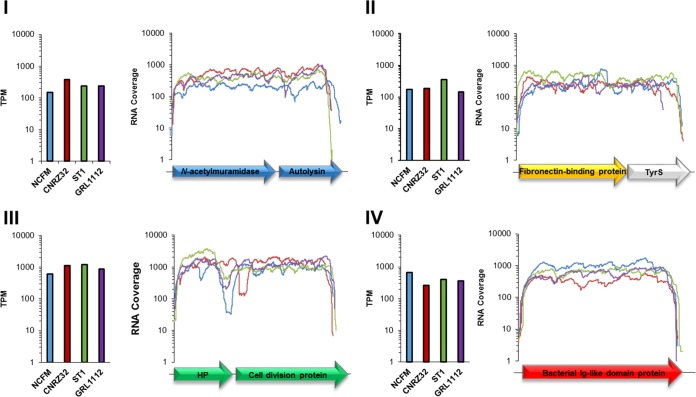
Transcription levels of the four conserved SLAP genomic regions were measured through RNA sequencing. (I to IV) Illustrated expression shown in each region: blue, region I; yellow, region II; green, region III; red, region IV. The bar graphs for each panel present the normalized TPM, while the line graphs present RNA coverage across each gene from the SLAP regions in L. acidophilus NCFM (light blue), L. helveticus CNRZ32 (dark red), L. crispatus ST1 (light green), and L. amylovorus GRL1112 (purple).

Whole-transcriptome profiling through deep RNA sequencing (RNA-seq) was employed to examine the global expression of the putative SLAP gene regions in L. acidophilus, L. helveticus, L. crispatus, and L. amylovorus. While expression was similar between the four strains in each gene region (Fig. 7, bar graphs), the gene regions were themselves expressed at different levels (Fig. 7, line graphs). Both regions I and II had expression levels between 100 and 500 TPM, while regions III and IV had expression levels of >1,000 TPM (Fig. 7). These data also confirmed the monocistronic expression of region IV and the predicted polycistronic expression of the *N*-acetylmuramidase and autolysin of region I. Conversely, the cell division genes in region III appeared to be monocistronically expressed. Surprisingly, the gene encoding a fibronectin-binding protein of region II was found to be polycistronically expressed, along with a tyrosyl-tRNA synthetase gene, *tyrS*. Finally, *in silico* promoter identification and analysis suggested that the *N*-acetylmuramidase gene and the group 3 bacterial Ig-like domain gene were under the constitutive transcriptional control of a putative σ^70^ (*rpoD*)-like promoter with a TANAAT −10 region consensus motif and an NTGTNT −35 region consensus motif (see Fig. S1 in the supplemental material). This promoter was found upstream of numerous housekeeping genes, including *ftsA*, *ldhD*, *secA*, and *eno* (see Fig. S1 in the supplemental material).

## DISCUSSION

Previous work has shown that the S-layers are more complex than previously understood. SLAPs were first identified in L. acidophilus NCFM and were hypothesized to scaffold to the cell wall with the S-layer (31). Additionally, a recent proteomic cell-shaving study in the S-layer-forming food bacterium Propionibacterium freudenreichii characterized various cell surface proteins, including putative SLAPs, for their anti-inflammatory immunomodulatory capacity (37). In the present study, we demonstrate that the presence or absence of an S-layer has a clear and direct impact on the exoproteomic composition of Lactobacillus species (Fig. 2). In S-layer-forming species of the L. acidophilus homology group, numerous noncovalently bound proteins were identified, which may be associated with the S-layer. In contrast, the few proteins that were isolated with LiCl treatment in the non-S-layer-forming strains were mostly intracellular proteins. These observations substantiate the aforementioned studies, lending credence to the existence of SLAPs as an integral component of the complex S-layer.

There were four protein domains found consistently within the putative SLAPs: BIg_3 (PF07523), SH3_8 (PF13457), fn3 (PF00041), and NCAD (PF03217). NCAD are predicted to be responsible for the noncovalent attachment of S-layer proteins to the cell wall in Lactobacillus species (38). Notably, there are extracellular proteins within the annotated proteome of L. delbrueckii subsp. bulgaricus that contain the NCAD. Similarly, the fn3 domain, an Ig-fold domain found in fibronectin-binding proteins, was also within the predicted proteomes of the non-S-layer-forming species L. gasseri and L. johnsonii. In both of these examples, the domains were ubiquitously identified in the noncovalently bound exoproteome fractions of the S-layer-forming strains but were not apparent in the exoproteomes extracted from the non-S-layer-forming strains. These observations suggest that the S-layer may be an important scaffold for extracellular proteins with NCAD.

From the numerous putative SLAPs, six were found to be conserved among the four S-layer-forming strains, L. acidophilus, L. crispatus, L. amylovorus, and L. helveticus, into four genomic regions. These four genomic regions include genes encoding the cell division protein CdpA, an *N*-acetylmuramidase, an uncharacterized fibronectin-binding protein, and an uncharacterized group 3 bacterial Ig-like domain protein. The cell division protein CdpA was first functionally described in L. acidophilus NCFM (39). Specifically, phenotypic analysis of a *cdpA* knockout strain revealed a strain with increased chain length, aberrant cell morphology, decreased resistance to environmental stressors, and decreased adhesion to Caco-2 epithelial cells (39). The direct mechanisms regarding the function of CdpA and the aforementioned phenotypes were unclear but were thought to be a pleiotropic response to the modified cell wall structure. Notably, the results of the current study offer further insight into this mechanism. First, the protein has two of the NCAD, suggesting localization to the cell wall along with the S-layer. Second, CdpA is one of the most prevalent SLAPs in the S-layer-forming strains but is not found in any non-S-layer-forming Lactobacillus species. It is possible that CdpA is a structural intermediary between the cell wall and the S-layer and other SLAPs during cell division. There is evidence for this in the original study in which the *cdpA*-deficient strain was treated with guanidine HCl, and the extracted extracellular SLAPs and SLPs were reduced compared to those of the parent strain (39). These observations indicate that CdpA may be an important component of S-layer structure and function.

The conserved SLAP gene regions were organized into four regions, which demonstrated remarkable conservation in genome position within the overall chromosome architectures (Fig. 5). Strand location of genes on the bacterial chromosome is an important factor for codon usage, which correlates with gene expression (40–42). Moreover, genes of low-G+C-content Gram-positive bacteria illustrate a strand bias for the positive and negative leading strands diverging from the origin of replication (43, 44). The conserved SLAP genes reflect this bias, as they were all found on the leading strands of the positive and negative strands of the chromosomes (Fig. 5).

The transcription of these genes, as measured by RNA sequencing, was similar among the four strains, albeit their rates of transcription were not uniform throughout all four gene regions (Fig. 7). In fact, the genes encoding the *N*-acetylmuramidase and group 3 bacterial Ig-like domain protein appeared to be under the control of a putative σ^70^(*rpoD*)-like promoter. The −10 region followed the TANAAT consensus described by Pribnow (45), while the −35 region followed an NTGTNT consensus. These motifs are similar to the σ^70^-like promoters of housekeeping genes identified in Lactobacillus plantarum (46). Housekeeping genes, such as *ftsA*, *ldhD*, *secA*, and *eno*, were identified as genes under similar transcriptional control.

Taken together, the genomic architecture and transcription data suggest that the conserved SLAPs found in the S-layer-forming strains of Lactobacillus are housekeeping genes expressed at constitutive levels. Given their conservation, we conclude that they likely participate in various essential cell processes, such as cell wall hydrolysis, maintenance of cell shape, protein turnover, and cell adhesion. It is notable that genes encoding SLAPs with rudimentary function, such as *cdpA* and the *N*-acetylmuramidase gene, are absent in non-S-layer-forming strains. There also remain the two uncharacterized proteins, the fibronectin-binding protein and the group 3 bacterial Ig-like domain proteins, which have yet to be functionally characterized and are functionally associated with S-layer-forming strains.

Given the extracellular localization of these proteins, the SLAPs identified in this study may have unexplored, potentially important roles in probiotic-host interactions and signaling. Among the conserved SLAPs explored, both the fibronectin-binding protein and the group 3 bacterial Ig-like domain protein have Ig-like folds within their respective amino acid tertiary structures, which may be involved in cell-to-cell adhesion or cell-to-host adhesion. Furthermore, all of these proteins, regardless of their cellular function, are accessible for intimate interactions with the gut epithelium and mucosal immune system (31, 37). In this study, all proteomic and genomic comparisons made for L. helveticus, L. crispatus, and L. amylovorus were made with only one respective genome for each species (L. helveticus CNRZ32, L. crispatus ST1, and L. amylovorus GRL1112). A more complete picture could be made if the genomes of each strain tested were utilized as proteomic and genomic references.

Despite being prevalent among all bacterial types, little is known about the evolutionary function of S-layers. Here, we present the S-layer as a scaffold for numerous noncovalently attached secreted proteins. These S-layer-associated proteins are conserved among S-layer-forming species and absent in non-S-layer-forming species. It is unambiguously clear that the noncovalent exoproteomes of the S-layer-forming strains are more diverse and dynamic than those of the non-S-layer-forming strains. The understanding of these exoproteins opens new avenues for the functional characterization of the S-layer and the health-promoting mechanisms of probiotic-host signaling and cross talk.

## Supplementary Material

Supplemental material
