# Supplementary material for: Conserved S-Layer-Associated Proteins Revealed by Exoproteomic Survey of S-Layer-Forming Lactobacilli
Source: Appl Environ Microbiol. 2015 Dec 22;82(1):134–45. doi: 10.1128/AEM.01968-15 (PMC4702614; doi:10.1128/AEM.01968-15)
Supplement: Supplemental material [file supp_82_1_134__index.html]

Conserved S-Layer-Associated Proteins Revealed by Exoproteomic Survey of S-Layer-Forming Lactobacilli — Supplemental material 

# Conserved S-Layer-Associated Proteins Revealed by Exoproteomic Survey of S-Layer-Forming Lactobacilli

## Supplemental material

- Supplemental file 1 -

  UniProt ID, molecular weight, and normalized spectral count of each protein identified after extraction of non-covalently bound exoproteomes of various S-layer and non-S-layer lactobacilli (Table S1); putative SLAPs identified in the non-covalently bound exoproteomes of the S-layer-forming strains (Table S2); putative promoter elements in the sequence directly upstream of the SLAP gene regions, and results of cotranscriptional analysis showing varying levels of expression of the 8 housekeeping genes examined (Fig. S1).

  PDF, 1005K
