# Supplementary material for: Conserved S-Layer-Associated Proteins Revealed by Exoproteomic Survey of S-Layer-Forming Lactobacilli
Source: Appl Environ Microbiol. 2015 Dec 22;82(1):134–45. doi: 10.1128/AEM.01968-15 (PMC4702614; doi:10.1128/AEM.01968-15)
Supplement: Supplemental material [file AEM.01968-15_zam999116813so1.pdf]

<u>Supplemental Tables and Figure:</u>

Supplemental Table 1: The non-covalently bound exoproteomes of various S-layer and non-S-layer lactobacilli were extracted with LiCl treatment and identified through LC-MS/MS. Strains included: S-layer forming *L. acidophilus*, *L. helveticus*, *L. crispatus*, and *L. amylovorus*; and non-S-layer forming *L. delbrueckii* subsp. *bulgaricus* and *L. casei*. Reported are the UniProt IDs of each identified protein, the molecular weight, and the normalized spectral count.

Supplemental Table 2: Putative SLAPs were identified in the non-covalently bound exoproteomes of the S-layer forming strains *L. acidophilus*, *L. helveticus*, *L. amylovorus*, and *L. crispatus*. Reported are the identified proteins, ORF, UniProt ID, molecular weight, identified pfam domains, Amino Acid coverage, and normalized spectral counts.

Supplemental Figure 1: To contextualize the transcriptome data, we performed *in silico* analyses in tandem with the RNA-seq to find putative promoter elements in the sequence directly upstream of the SLAP gene regions. Two similar promoter elements were discovered upstream of the *N*-acetylmuramidase (*Nmur*) of region I and the group 3 bacterial Ig-like domain gene (*BIg3*) of region IV. For both promoters the -10 region consisted of a TANAAT consensus motif, while the -35 region followed a NTGTNT consensus motif, in which N represents any nucleotide (A). There was a spacing of 17 nucleotides between the -35 and -10 sites of the *Nmur* promoter, but the spacing was 23 nucleotides for the *BIg3* promoter (B, bolded). Despite this discrepancy, the level of expression was comparable for each gene. In fact, the *BIg3* gene was more highly expressed than *Nmur*, notwithstanding the increased spacing between the -10 and -35 sites (C). The promoters containing the TANAAT and NTGTNT consensus motifs with a spacing of 16 to 23 base pairs in the upstream untranslated regions within the four genomes were curated (8B, non bolded). Numerous housekeeping genes were found to be downstream of the putative

promoters, including the β subunit of RNA polymerase (*rpoB*), initiation factor A (*infA*), cell division protein, *ftsA*, *D*-lactate dehydrogenase (*ldhD*), the protein translocase subunit, *secA*, enolase (*eno*), *lex*A repressor, and *slpX*. Finally, co-transcriptional analysis using normalized TPM revealed that the 8 housekeeping genes examined had varying levels of expression (C). Distance between the -10 and -35 site did not appear to have any effect on expression. For example, the gene with the highest expression, *eno*, had 20 base pairs between the -10 and -35. Additionally, the *infA* had much higher expression than *rpoB* even though they both had a separation of 16 base pairs between -10 and -35 promoter elements (B).

Supplemental Table 1: Identified proteins in the non-covalently bound exoproteomes of S-layer and non-S-layer forming lactobacilli

Identified Proteins		Spectral counts								
Uniprot ID	Molecular '	**L. crispatus CZ6**	**L. amylovorus ATCC 3620**	**L. casei ATCC 393**	**L. bulgaricus**	**L. helveticus CNRZ32**	**L. helveticus 481-C**	**L. helveticus ATCC15009**	**L. crispatus ATCC33820**	**L. crispatus CI**
tr|C2KDQ5|C2KD	27 kDa	1181	237	9	0	47	42	18	1698	963
tr|D5H1S0|D5H	53 kDa	850	79	13	1	12	3	3	1176	146
tr|U2HNM4|U2	18 kDa	241	63	10	0	11	3	2	477	136
tr|F0NVR1|F0N	56 kDa	182	45	0	0	0	0	0	371	88
tr|U2H0C9|U2H	6 kDa	88	0	0	0	0	0	0	184	1
tr|F0TJ46|F0TJ	54 kDa	59	516	15	0	5	0	1	47	36
tr|C2HMW6|C2	54 kDa	59	225	13	0	8	0	0	157	99
tr|Q9L5V6|Q9L	53 kDa	0	75	3	0	0	0	0	82	72
tr|C2ELK0|C2EI	55 kDa	0	0	0	0	0	0	0	93	0
tr|D5H0Y7|D5H	63 kDa	802	196	5	0	9	0	4	1428	1555
tr|D4FH50|D4F	63 kDa	696	193	5	0	6	3	5	1219	1425
tr|C2KDQ6|C2K	63 kDa	644	193	5	0	8	0	2	1274	1753
tr|D0DIV9|D0D	63 kDa	590	111	2	0	3	0	6	1138	1117
tr|C2KBQ5|C2K	61 kDa	749	48	0	0	13	10	18	773	431
tr|F0TE79|F0TE	61 kDa	117	395	5	0	71	25	24	140	59
tr|C2HND8|C2H	62 kDa	70	90	0	0	66	29	36	115	57
tr|C2EM32|C2E	59 kDa	61	171	1	0	89	36	37	105	47
tr|C9M2I1|C9M	61 kDa	22	72	0	0	557	395	569	118	18
tr|A8YUE0|A8Y	61 kDa	22	71	0	0	482	434	502	70	18
tr|F6CEJ2|F6CE	61 kDa	4	47	1	0	73	56	62	4	9
tr|D5H2K8|D5H	105 kDa	518	0	0	0	0	0	0	0	0
tr|E4SK60|E4SI	42 kDa	0	11	0	0	3	0	0	0	0
tr|D4YTJ2|D4Y	44 kDa	0	0	0	0	0	2	1	0	0
tr|C2KBR4|C2K	56 kDa	422	0	0	0	0	0	0	704	292
tr|D4FCG0|D4F	56 kDa	356	0	0	0	0	0	0	560	215
tr|F2LZ27|F2LZ	55 kDa	0	0	0	0	0	0	0	30	21
tr|I7K169|I7K1	28 kDa	0	0	0	0	0	0	0	50	0
tr|D5GYT4|D5GY	44 kDa	359	0	0	0	0	14	0	265	249
tr|K1MC51|K1M	78 kDa	294	2	0	0	0	0	0	0	0
tr|D5GXP1|D5G	79 kDa	258	0	0	0	0	0	0	0	0
tr|C7XHB7|C7X	79 kDa	183	0	0	0	0	0	0	0	0
tr|Q9Z4J9|Q9Z	47 kDa	143	0	0	0	0	32	0	0	19
tr|C2KD47|C2K	27 kDa	111	0	0	0	0	26	9	198	196
tr|C7XM73|C7X	32 kDa	99	0	0	0	0	6	0	0	0
tr|C7Y4D6|C7Y	47 kDa	98	1	0	0	1	55	16	9	12
tr|F8T7L9|F8T7	47 kDa	91	0	0	0	0	0	0	8	0
tr|R5ZBK8|R5Z	49 kDa	61	0	0	0	85	0	33	0	9
tr|E2IPN7|E2IP	43 kDa	54	0	0	0	130	192	48	0	0
tr|A8YX12|A8Y	49 kDa	52	0	0	0	0	108	40	0	0
tr|F0TI54|F0TI	50 kDa	40	29	0	0	30	34	3	0	13
tr|Q09FM2|Q0	47 kDa	38	7	0	0	0	0	9	9	11
tr|Q56TM2|Q5	44 kDa	38	0	0	0	56	0	31	1	22
tr|Q56TM9|Q5	40 kDa	37	0	0	0	0	68	0	1	12
tr|Q8VW91|Q8	48 kDa	36	0	0	0	211	331	58	27	0
tr|O07120|O07	47 kDa	32	11	0	0	32	29	0	8	0
tr|I7JZZ0|I7JZZ	14 kDa	26	7	0	0	0	0	0	10	11
tr|F6CFK7|F6CI	49 kDa	25	7	0	0	31	323	10	8	10
tr|S5DTT0|S5D	47 kDa	24	61	0	0	688	138	74	32	6
tr|Q8VW90|Q8	41 kDa	24	98	0	0	206	156	274	59	9
tr|Q8VW92|Q8	48 kDa	15	42	0	0	210	282	49	19	0
tr|L7YE91|L7YE	34 kDa	14	0	0	0	60	318	3	0	0
tr|K8DVK7|K8D	35 kDa	0	82	0	0	128	79	143	24	0
sp|P35829|SLA	47 kDa	0	40	0	0	34	0	16	0	0
tr|F3MND6|F3	11 kDa	0	0	0	0	149	121	65	30	0
tr|Q9XB19|Q9	46 kDa	0	96	0	0	359	138	138	39	0
tr|C2HLL1|C2H	9 kDa	0	8	0	0	35	0	0	0	0
tr|Q9S396|Q9S	47 kDa	0	63	0	0	234	159	185	49	0
tr|Q09FL7|Q09	48 kDa	0	0	0	0	0	0	0	8	19
tr|Q6DV80|Q6	39 kDa	0	0	0	0	109	114	47	0	0
tr|Q56TM1|Q5	40 kDa	0	0	0	0	58	0	0	0	0
tr|Q56TM8|Q5	39 kDa	0	77	0	0	0	0	0	0	0
tr|R5YRQ3|R5Y	28 kDa	226	650	30	0	43	17	18	186	157
tr|C2EQL6|C2E	29 kDa	216	197	8	0	186	76	161	250	167
tr|F6CFQ2|F6C	29 kDa	136	161	7	0	234	125	209	130	91
tr|C2HM24|C2	30 kDa	130	195	0	0	22	18	31	113	85
tr|A8YX60|A8Y	40 kDa	52	47	10	0	326	1774	607	140	59
tr|S5E4S6|S5E	37 kDa	16	37	7	2	728	771	1242	209	19
tr|J4BT78|J4BT	7 kDa	0	0	0	0	9	0	50	5	0
tr|J4BP74|J4BP	32 kDa	0	0	0	0	280	586	281	0	0
tr|C7XIS9|C7XI	66 kDa	195	21	0	0	0	0	0	557	4
tr|E4SKB5|E4SI	66 kDa	37	99	0	3	9	9	16	102	2
tr|C2EMF4|C2E	66 kDa	37	24	0	0	12	14	22	104	0
tr|F0NSB2|F0N	66 kDa	35	28	0	0	0	0	0	74	0
tr|D5GY99|D5G	66 kDa	14	21	0	0	0	0	0	52	0
tr|C4VLD0|C4V	51 kDa	11	19	0	0	0	0	0	37	0
tr|A4ZH36|A4Z	65 kDa	8	0	0	0	0	0	0	42	0
tr|F4AGJ7|F4A	65 kDa	2	0	0	1	0	0	1	30	0
tr|D5H0C4|D5H	42 kDa	199	1	0	0	0	13	0	346	1
tr|D5H2B5|D5H	29 kDa	178	0	0	0	0	0	0	16	208
tr|C2KBR2|C2K	29 kDa	159	0	0	0	0	0	0	11	185
tr|C7XL60|C7X	30 kDa	154	55	28	8	3	14	2	105	13
tr|E4SKX0|E4SI	30 kDa	92	73	0	8	4	14	0	72	7
sp|A8YXK8|RL2	30 kDa	92	55	0	8	0	20	8	72	7
tr|C2EQV0|C2E	29 kDa	72	27	0	8	0	9	0	65	5
tr|N1ZQT8|N1Z	30 kDa	55	18	0	6	0	0	2	41	6
sp|Q04C12|RL2	30 kDa	24	15	25	48	0	5	0	11	0
sp|Q38UR5|RL2	30 kDa	22	14	36	0	1	0	0	13	0
sp|B3WAL4|RL	30 kDa	17	6	143	1	3	3	2	4	2
tr|G2KVY9|G2K	31 kDa	13	0	0	0	0	0	0	0	0
sp|Q88XY3|RL2	30 kDa	0	0	29	6	0	0	0	10	0
sp|Q03PW0|RL	31 kDa	0	5	28	0	0	0	0	0	0
tr|C2HQ77|C2H	66 kDa	61	18	0	0	7	6	4	3	0
tr|F2M0I5|F2M	66 kDa	61	18	0	0	15	9	8	4	0

tr|C2EMF3|C2E	66 kDa	52	0	0	0	0	0	0	0	0
tr|F3MLV0|F3M	65 kDa	45	38	0	14	108	181	244	27	0
tr|S5E085|S5E(	65 kDa	45	38	0	0	108	158	249	24	0
tr|A8YVU4|A8Y	65 kDa	39	38	0	0	84	150	226	24	0
tr|F0NSB1|F0N	66 kDa	39	40	0	0	65	116	184	24	0
tr|I7JZC4|I7JZC	48 kDa	18	29	0	0	5	0	9	34	0
tr|E4SL67|E4SL	65 kDa	13	44	0	0	8	11	9	17	0
tr|I7LEY1|I7LEY	66 kDa	12	0	0	0	0	6	7	5	0
tr|F0TFS6|F0TF	66 kDa	12	151	0	0	17	25	39	18	1
tr|C2KFZ5|C2K	25 kDa	6	7	0	0	22	20	25	19	0
tr|I7KG73|I7KG	65 kDa	4	0	0	0	16	9	21	2	0
tr|D5H0E5|D5H	66 kDa	2	1	0	12	6	3	7	49	11
tr|G6F4C8|G6F	66 kDa	0	0	0	7	14	9	17	1	0
tr|F4ACM2|F4/	66 kDa	0	0	0	0	7	0	0	3	0
tr|C2KDX3|C2K	23 kDa	0	0	0	4	0	0	0	16	5
tr|K0NHV5|K0N	67 kDa	0	0	0	0	0	0	17	2	0
tr|C2HQH3|C2H	67 kDa	0	36	0	0	0	0	0	0	0
tr|C7XJD8|C7X.	48 kDa	141	6	0	0	9	7	15	83	17
tr|C2EPU3|C2E	47 kDa	71	5	0	0	9	0	15	33	8
tr|E4SMC9|E4S	47 kDa	62	25	0	0	0	3	11	35	6
tr|C9M3W3|C9	48 kDa	34	0	0	0	27	19	35	22	9
tr|F0NW69|F0I	48 kDa	30	9	0	0	4	5	13	18	4
tr|C2KET8|C2KET	39 kDa	136	0	0	0	0	0	0	3	8
tr|C2KGZ0|C2K	22 kDa	131	17	0	0	1	0	0	83	7
tr|E4SKN8|E4S	22 kDa	56	85	0	0	1	0	1	37	3
tr|C2KBF7|C2KBF	22 kDa	130	9	0	0	4	0	0	139	148
tr|D5H2S7|D5H	44 kDa		70	36	129	16	0	67	33	79
tr|C9M0F6|C9M	44 kDa	73	59	36	129	25	0	86	30	56
tr|S6DLG9|S6D	44 kDa	48	63	10	98	14	0	48	20	44
tr|D4YS23|D4Y	44 kDa	46	25	29	145	5	0	36	7	35
tr|Q8KMQ9|Q8	28 kDa	45	8	30	269	0	0	31	0	24
sp|Q04B37|EFT	43 kDa	41	8	46	372	1	0	26	18	22
sp|B3WE38|EF	44 kDa	37	8	136	88	0	0	21	18	18
tr|M9T1J1|M9T	27 kDa	28	6	31	64	9	0	31	5	20
tr|C7XTP2|C7X	44 kDa	20	8	21	37	0	0	0	0	0
tr|H1LCP2|H1L	43 kDa	8	6	36	21	0	0	0	0	0
tr|J3JBI8|J3JBI8	44 kDa	1	0	25	29	0	0	0	0	0
tr|C4RA50|C4R	36 kDa	0	0	28	0	0	0	0	0	0
tr|C5J1V4|C5J1	30 kDa	0	0	111	77	0	0	0	0	0
tr|C8PAS6|C8P	43 kDa	0	0	33	117	0	0	13	0	0
tr|Q599J5|Q59	18 kDa	0	0	17	1	0	0	0	0	0
tr|E3SW19|E3S	31 kDa	0	7	8	52	0	0	0	0	0
tr|C5J1V9|C5J1	32 kDa	0	0	111	71	0	0	0	0	0
tr|G6CGX7|G6(	43 kDa	0	8	6	15	0	0	0	0	0
tr|C5J229|C5J2	32 kDa	0	0	22	10	0	0	0	0	0
tr|C5J1Y1|C5J1	29 kDa	0	0	111	0	0	0	0	0	0
tr|D5H393|D5H3	52 kDa	119	0	0	0	0	0	0	114	111
tr|D5GYI3|D5G	27 kDa	115	3	2	0	3	0	4	68	38
tr|E3R5W0|E3I	27 kDa	83	2	1	0	3	0	3	236	111
tr|E4SL88|E4SL	28 kDa	1	0	0	0	0	0	19	4	1
tr|J3ZDC4|J3ZD	28 kDa	0	0	0	0	11	10	257	82	1
tr|A8YW20|A8Y	25 kDa	0	0	0	0	6	12	145	60	1
tr|F0NWW5|F(	27 kDa	0	0	0	0	6	7	159	63	0
tr|C2KGY6|C2K	18 kDa	109	79	7	20	37	16	49	96	10
sp|A8YXK2|RS7	18 kDa	99	66	18	16	46	18	60	89	8
tr|I7LB13|I7LB1	18 kDa	35	78	8	20	35	5	30	42	6
sp|B3WAM3|R	18 kDa	0	5	120	0	1	0	7	0	0
sp|Q38UQ8|RS	18 kDa	0	3	21	0	0	0	0	0	0
tr|C2KE29|C2K	33 kDa	105	3	0	0	9	12	8	49	12
tr|F6CDL2|F6C	33 kDa	43	5	0	0	9	18	12	26	5
tr|A4UAE0|A4U	33 kDa	26	3	0	0	18	35	22	20	4
tr|I7LD71|I7LD	33 kDa	12	0	0	0	0	0	0	0	0
tr|C7XHP3|C7X	23 kDa	101	25	18	18	4	3	5	99	12
sp|A8YUL2|RS4	23 kDa	35	59	18	0	10	3	27	26	4
sp|Q04B92|RS4	24 kDa	24	5	15	107	0	0	0	24	4
tr|I7K127|I7K1	23 kDa	24	37	15	38	0	0	9	24	0
tr|C4VMR6|C4V	23 kDa	23	6	10	16	0	0	0	30	4
tr|C8PDK6|C8P	24 kDa	8	0	10	0	0	0	0	7	0
tr|C2KGD6|C2K	42 kDa	97	0	0	0	0	0	0	108	0
tr|C2D336|C2D	39 kDa	11	0	0	0	0	0	0	0	0
tr|C2KFW2|C2I	29 kDa	94	119	3	0	137	9	51	85	23
sp|A8YVR9|RS2	29 kDa	63	131	7	35	166	13	77	68	17
sp|Q74IR7|RS2	29 kDa	51	87	0	49	78	0	37	40	0
tr|I7L8Y7|I7L8Y	29 kDa	33	62	0	29	47	3	29	32	9
tr|C4VLA7|C4V	29 kDa	19	50	0	57	58	0	26	0	0
tr|C0XLI3|C0XL	30 kDa	13	19	10	0	20	0	0	15	0
sp|B3WES8|RS	30 kDa	12	34	47	20	25	3	15	16	3
sp|Q049U2|RS	28 kDa	3	20	6	144	25	3	17	15	1
tr|H1X5E7|H1X	29 kDa	0	0	8	12	17	0	0	15	3
tr|C7XTR6|C7X	29 kDa	0	0	11	0	0	0	0	0	0
tr|H4GKR7|H4(	29 kDa	0	34	0	0	0	0	0	0	0
tr|F2M168|F2M	62 kDa	95	413	9	0	18	6	8	131	142
tr|E4SKI2|E4SK	62 kDa	91	391	11	0	19	6	5	131	142
tr|C2KCU0|C2K	58 kDa	91	5	3	5	27	0	68	11	16
tr|E4SLF6|E4SL	58 kDa	52	6	3	0	32	0	85	7	9
sp|A8YTH8|CH	58 kDa	40	4	5	0	37	0	138	13	9
sp|O68324|CH	58 kDa	38	2	5	0	48	0	154	14	9
tr|C5J3D2|C5J3	20 kDa	32	0	4	0	16	0	53	7	10
tr|I7LGV1|I7LG	57 kDa	28	0	3	0	19	0	55	4	8
tr|F0JZ61|F0JZ(	57 kDa	20	0	1	19	5	0	8	5	7
tr|C5J3K2|C5J3	21 kDa	18	0	5	0	22	0	82	8	7
sp|B3W9W7|C	57 kDa	12	0	22	0	12	0	30	4	6
tr|D7V4Z7|D7V	58 kDa	11	0	8	0	12	0	23	0	0
tr|N1ZV35|N1Z	57 kDa	10	0	8	0	0	0	0	0	0
tr|B3XQT7|B3X	57 kDa	8	0	3	1	6	0	17	3	0
tr|C5J349|C5J3	20 kDa	0	0	8	0	0	0	11	0	0

tr|R9GCH4|R9(	57 kDa	0	0	3	0	0	0	18	0	0
tr|Q2Z1H5|Q2.	20 kDa	0	0	0	0	0	0	0	0	0
tr|D5GY85|D5(	42 kDa	92	0	0	0	0	0	0	81	99
tr|C2KFX7|C2K	42 kDa	86	0	0	0	0	0	0	80	89
tr|C2KEW4|C2I	34 kDa	66	10	0	0	4	35	21	93	23
tr|F0NS42|F0N	35 kDa	44	16	0	0	4	99	57	64	17
tr|A8YTM0|A8'	30 kDa	42	0	0	0	4	124	48	63	13
tr|N2AC72|N2/	45 kDa	23	19	0	0	0	14	0	25	13
tr|C7XIX1|C7XI	44 kDa	21	14	0	0	7	24	23	203	96
tr|F0TFW7|F0T	44 kDa	15	137	0	0	0	0	0	44	18
tr|C7Y7D0|C7Y	42 kDa	11	38	0	0	0	12	10	43	7
tr|C9M0W5|C9	29 kDa	0	0	0	0	3	80	63	22	8
tr|E4SNP9|E4S	35 kDa	0	14	0	0	0	18	0	0	0
tr|C2EMB2|C2I	46 kDa	0	53	0	0	0	0	39	0	0
tr|C2KGY7|C2K	77 kDa	88	34	4	136	6	0	38	6	14
sp|Q5FM92|EF	77 kDa	71	25	4	134	9	0	46	5	10
tr|C9M229|C9I	77 kDa	65	25	4	136	9	0	56	6	10
tr|N1ZQU4|N1	77 kDa	47	14	4	129	6	0	33	4	7
sp|Q04C17|EF(	77 kDa	35	13	5	295	1	0	25	4	7
tr|C8PCE5|C8P	77 kDa	18	12	4	137	0	0	23	4	3
tr|M5J726|M5.	77 kDa	12	5	7	54	0	0	11	3	0
sp|B3WAM2|E	77 kDa	3	3	46	25	0	0	2	0	0
sp|B2GDX1|EF(	76 kDa	0	0	5	17	0	0	0	0	0
tr|C2EV93|C2E	77 kDa	0	0	0	16	0	0	0	0	0
tr|K0NP44|K0N	77 kDa	0	0	0	268	0	0	0	0	0
tr|F4FSR5|F4F	77 kDa	0	0	11	32	0	0	0	0	0
tr|F7QRW9|F7(	78 kDa	0	0	0	16	0	0	0	0	0
tr|D5H370|D5I	63 kDa	75	54	0	18	55	1	123	21	26
tr|C9M016|C9I	63 kDa	67	65	0	18	62	0	153	22	26
tr|F0NTC5|F0N	64 kDa	59	64	0	18	58	0	132	21	24
tr|F6CB49|F6C	63 kDa	50	59	0	18	47	0	105	16	17
tr|N2A671|N2/	63 kDa	35	26	0	17	23	0	59	8	14
tr|C2KGL0|C2K	44 kDa	67	70	0	55	27	0	50	12	22
tr|E4SIH2|E4SI	44 kDa	51	100	1	57	38	0	57	15	15
tr|F6CB39|F6C	44 kDa	45	44	0	55	36	0	58	12	15
tr|C9M005|C9I	44 kDa	42	51	0	39	61	0	87	15	14
tr|C2E5R0|C2E	44 kDa	35	42	0	0	30	0	49	11	14
tr|I7IZW0|I7IZ\	44 kDa	31	36	0	0	19	0	48	0	11
tr|C4VMS3|C4'	44 kDa	0	7	0	24	0	0	33	0	0
tr|C8PAP7|C8P	43 kDa	0	31	0	0	0	0	0	0	0
tr|E3R1A8|E3R	34 kDa	69	0	0	2	0	0	0	38	8
tr|C2EQ10|C2E	34 kDa	29	1	0	0	0	0	0	19	7
tr|I7L5J3|I7L5J.	34 kDa	12	0	0	0	0	0	0	4	0
tr|K0NV95|K0N	34 kDa	3	0	0	41	0	0	0	3	0
tr|F0JZ01|F0JZ(	34 kDa	2	0	0	72	0	0	0	2	0
tr|G6EWB3|G6	34 kDa	0	0	0	61	0	0	0	0	0
tr|C2KH58|C2K	58 kDa	66	74	0	43	124	2	50	73	2
tr|S5DXP8|S5D	58 kDa	54	65	0	43	131	0	55	64	2
tr|F6CD33|F6C	57 kDa	53	58	0	39	108	2	47	65	2
sp|Q74L27|SYE	57 kDa	34	18	0	40	22	0	22	23	0
sp|Q048S3|SYE	57 kDa	31	17	0	98	20	0	21	24	2
tr|C8PBH8|C8F	57 kDa	29	9	0	33	3	0	15	22	0
tr|K0NQN9|K0I	57 kDa	29	2	0	73	8	0	16	23	0
tr|H3RQR7|H3I	58 kDa	27	0	0	0	0	0	15	0	0
sp|Q38YY5|SYE	57 kDa	26	1	0	34	4	0	15	19	0
tr|G9ZMK3|G9.	55 kDa	22	0	0	0	0	0	0	0	0
tr|D0DYI6|D0D	57 kDa	8	9	0	29	3	0	9	6	0
tr|C2EQZ8|C2E	58 kDa	0	76	0	0	118	0	52	66	0
tr|J0L674|J0L6'	56 kDa	0	1	0	0	0	0	0	19	0
tr|J2Z4L3|J2Z4I	56 kDa	0	0	0	11	0	0	0	0	0
tr|N1ZE78|N1Z	57 kDa	0	0	0	0	0	0	15	0	0
sp|Q03SU7|SYI	57 kDa	0	0	0	0	0	0	0	19	0
tr|D5H2U3|D5I	39 kDa	67	7	0	0	5	4	4	29	28
tr|R5YIL7|R5YII	41 kDa	1	54	0	0	7	14	11	1	1
tr|D5H0B8|D5I	92 kDa	54	32	0	34	63	0	34	18	0
tr|S5DWM8|S5	92 kDa	50	32	0	23	72	0	43	17	2
tr|C2KCN2|C2K	92 kDa	49	30	0	29	59	0	32	16	0
tr|C2ENW0|C2	92 kDa	45	27	0	34	55	0	34	16	0
tr|F6CF31|F6CI	92 kDa	41	18	0	23	45	0	28	11	0
tr|I7LAM3|I7LA	93 kDa	18	11	0	0	18	0	10	3	0
tr|C4VNE1|C4\	92 kDa	12	6	0	26	14	0	7	0	0
tr|F0K388|F0K.	92 kDa	10	4	0	142	5	0	4	2	0
tr|Q1GC38|Q1(	92 kDa	10	0	0	137	5	0	0	0	0
tr|C8PB12|C8P	91 kDa	9	2	0	11	8	0	4	0	0
tr|C6VIH5|C6V	95 kDa	1	0	3	13	0	0	0	2	0
tr|C2KC97|C2K	47 kDa	55	21	0	23	22	0	53	14	108
tr|E4SIA4|E4SI	47 kDa	27	29	0	32	25	0	86	10	42
tr|C9M0A5|C9I	47 kDa	21	15	0	0	28	0	112	10	46
sp|A8YUV4|EN	47 kDa	21	15	0	0	28	0	113	10	46
sp|Q043Z5|EN(	47 kDa	19	15	0	28	19	0	58	6	44
tr|I7LAF5|I7LAI	47 kDa	17	15	0	32	19	0	38	0	40
tr|D5GZY0|D5(	44 kDa	53	0	0	0	0	0	0	119	0
tr|C2KF05|C2K	44 kDa	52	0	0	0	6	0	0	127	0
tr|E4SND3|E4S	44 kDa	22	0	0	0	11	0	0	28	0
tr|I7IVU5|I7IVU	43 kDa	20	0	0	0	0	0	0	0	0
tr|J7LIA5|J7LIA	44 kDa	13	0	0	0	23	0	23	9	0
tr|C2KDG2|C2I	47 kDa	53	0	0	0	0	0	0	24	26
tr|C2KH12|C2I	13 kDa	52	17	7	5	0	0	1	41	4
tr|E4SKZ1|E4SI	13 kDa	34	26	7	5	0	0	1	30	4
tr|E4SWH8|E4!	13 kDa	13	9	7	13	0	0	0	15	3
sp|B3WAJ4|RS	13 kDa	9	8	59	4	0	0	0	8	2
tr|J3JB44|J3JB4	14 kDa	0	0	12	2	0	0	0	4	0
tr|D5GZ50|D5(	23 kDa	47	483	13	1	31	1	2	13	54
tr|A8YWG4|A8	24 kDa	7	105	6	0	168	0	1	2	23
tr|J3X9R8|J3X9	24 kDa	7	105	6	0	124	0	0	0	23
tr|I7KPU2|I7KP	19 kDa	1	27	0	0	11	0	0	0	2

tr|C2KE06|C2K	8 kDa	1	14	0	0	0	0	0	0	7
tr|A8YWD2|A8YV	14 kDa	45	48	1	17	34	5	8	32	10
tr|D4FE26|D4F	40 kDa	41	0	0	0	0	0	0	91	25
tr|C2KB49|C2K	40 kDa	29	0	0	0	0	0	0	71	25
tr|C2KGM6|C2	19 kDa	42	0	0	0	0	0	0	99	30
tr|K1NFD6|K1N	10 kDa	38	0	0	0	0	0	0	92	28
sp|A8YXL4|RS1	11 kDa	43	12	0	5	16	15	10	24	5
sp|Q04C06|RS	11 kDa	21	3	0	15	4	7	3	8	1
tr|I7J269|I7J26	31 kDa	40	48	0	0	0	0	0	30	44
tr|F2M1V1|F2N	72 kDa	28	449	0	0	0	173	0	23	20
tr|J4BVJ4|J4BV	28 kDa	28	48	0	0	0	0	0	23	20
tr|J4BTR2|J4BT	71 kDa	0	251	0	0	0	262	0	0	0
tr|F0TGG1|F0T	71 kDa	0	362	0	0	0	120	0	0	0
tr|C2EL23|C2EI	72 kDa	0	22	0	0	0	13	0	0	0
tr|E4SLY7|E4SL	71 kDa	0	325	0	0	0	125	0	0	0
tr|N1ZH29|N1ZH	66 kDa	38	0	0	0	11	0	5	3	0
tr|C2EQY0|C2E	14 kDa	36	5	0	7	6	0	0	7	2
tr|N1ZQQ7|N1	14 kDa	18	3	0	7	0	0	0	2	0
tr|A8YTB2|A8Y	14 kDa	13	2	0	7	9	0	1	3	1
tr|D5GY60|D5(	97 kDa	35	16	2	4	21	13	9	17	2
sp|A8YVQ7|IF2	97 kDa	19	16	2	4	31	21	14	8	2
tr|F0TFM5|F0T	97 kDa	19	22	2	0	21	13	8	8	2
tr|C9LZ88|C9L	98 kDa	19	16	0	0	32	19	14	8	2
sp|Q5FJN6|IF2_	98 kDa	14	11	0	0	14	11	8	8	0
tr|D4YUU7|D4	58 kDa	13	7	0	2	11	6	6	6	0
tr|C2KCN9|C2K	34 kDa	35	3	0	0	2	0	2	73	2
tr|C2ENV2|C2E	34 kDa	21	3	0	0	0	0	0	62	0
tr|C2KGZ5|C2K	25 kDa	33	25	2	9	9	13	9	39	7
tr|C2EQV3|C2E	25 kDa	30	25	0	0	0	0	9	35	0
sp|A8YXL1|RS3	25 kDa	26	30	2	12	10	20	12	27	7
tr|E4SKX3|E4SI	25 kDa	26	36	0	12	11	14	9	33	7
tr|F0JZX3|F0JZ	25 kDa	7	23	7	58	5	5	4	12	4
sp|Q034Y9|RS	25 kDa	0	0	50	7	0	0	1	2	1
tr|D5H0T3|D5H	49 kDa	34	21	0	0	151	80	24	157	3
tr|J7LHE7|J7LH	39 kDa	0	0	0	0	0	0	0	0	0
tr|F3ML23|F3N	42 kDa	34	0	0	0	16	187	4	40	0
tr|S5DTK6|S5D	41 kDa	0	0	0	0	20	200	6	24	0
tr|C9M1B8|C9I	41 kDa	0	0	0	0	17	174	0	24	0
tr|C2KB82|C2K	42 kDa	33	0	0	0	0	1	0	4	0
tr|A8YTZ4|A8Y	41 kDa	12	0	0	0	0	2	0	0	0
tr|A8YVY6|A8Y	44 kDa	33	0	0	0	64	70	82	100	31
tr|J3XA08|J3XA	43 kDa	31	9	0	0	3	36	44	76	17
tr|F0NRS9|F0N	45 kDa	0	0	0	0	14	50	66	98	31
tr|C7XL73|C7XI	13 kDa	28	31	1	3	22	1	6	12	0
tr|E4SKY3|E4SI	13 kDa	14	60	1	0	24	1	4	2	0
tr|D8FPG0|D8F	13 kDa	8	4	4	14	0	0	0	5	0
sp|Q04BZ9|RL1	13 kDa	6	0	4	14	0	0	0	4	0
tr|F4FSP7|F4FS	13 kDa	2	10	5	0	8	0	0	1	0
tr|C9M247|C9I	13 kDa	0	0	0	0	33	3	8	0	0
tr|C2KBZ5|C2K	46 kDa	30	12	0	7	9	4	2	16	0
tr|D5H2J7|D5H	46 kDa	24	17	0	0	11	0	4	8	0
sp|Q5FKW9|TH	46 kDa	24	12	0	0	11	0	4	8	0
tr|C9M2S0|C9N	46 kDa	19	11	0	8	20	6	9	8	0
tr|D5H2B9|D5H	37 kDa	29	0	5	279	4	0	11	2	112
tr|F0NUV8|F0N	37 kDa	24	0	5	327	4	0	11	2	71
tr|F6CEI9|F6CE	37 kDa	8	0	2	81	0	0	6	0	30
tr|K0NKE8|K0N	37 kDa	3	0	11	727	4	0	2	2	19
tr|Q04BH2|Q0	37 kDa	3	0	8	666	4	0	2	0	18
tr|B3WCW4|B3	37 kDa	0	0	36	124	1	0	0	0	1
tr|S4PMX9|S4F	36 kDa	0	0	0	21	0	0	0	0	0
tr|C7XW46|C7	36 kDa	0	0	7	125	0	0	0	0	2
tr|C2ETG1|C2E	36 kDa	0	0	7	121	0	0	0	0	0
tr|K5EN00|K5E	36 kDa	0	0	0	48	0	0	0	0	0
tr|N2A0J5|N2A	36 kDa	0	0	7	90	0	0	0	0	18
tr|C8P6V4|C8P	36 kDa	0	0	0	124	0	0	0	0	0
tr|I7LBT6|I7LBT	36 kDa	0	0	0	258	0	0	0	0	0
tr|F0K1U9|F0K1U	44 kDa	28	22	0	162	9	0	23	8	11
tr|C2KD87|C2K	17 kDa	23	8	0	0	7	1	3	31	1
tr|N1ZXF5|N1Z	20 kDa	7	0	0	0	2	0	1	12	1
tr|F0TGC2|F0T	17 kDa	6	14	0	0	7	2	5	8	1
tr|C2HQW2|C2	17 kDa	6	9	0	0	7	2	7	16	0
tr|C2KH01|C2K	20 kDa	26	23	0	16	5	0	13	32	11
tr|E4SKX9|E4SI	20 kDa	25	38	0	16	5	0	14	28	10
sp|A8YXL7|RL5	20 kDa	23	30	0	0	0	0	11	17	7
tr|C9M243|C9I	20 kDa	19	27	0	19	7	0	18	25	7
sp|Q04C03|RL5	20 kDa	3	8	1	42	6	0	7	12	5
tr|D0DFA9|D0I	29 kDa	26	0	0	6	0	0	0	5	0
tr|G6EUB5|G6I	29 kDa	1	2	0	36	0	0	0	0	0
tr|F0K1S4|F0K	25 kDa	0	2	0	47	0	0	0	0	0
sp|A8YXM9|RS	14 kDa	25	26	25	24	0	3	0	21	1
sp|Q04BZ0|RS	14 kDa	14	22	26	36	0	1	0	11	0
sp|B3WAJ3|RS	14 kDa	14	23	43	24	0	0	0	11	0
tr|I7IVC9|I7IVC	14 kDa	4	3	16	9	0	0	0	0	0
tr|G2KVW7|G2	11 kDa	0	0	15	0	0	0	0	0	0
tr|J0KYU7|J0KY	14 kDa	0	20	19	0	0	1	0	10	0
sp|B2GDU4|RS	14 kDa	0	0	12	0	0	0	0	0	0
tr|C2KD46|C2K	25 kDa	25	0	0	0	0	0	0	26	23
sp|A8YXK5|RL3	23 kDa	24	25	0	50	0	0	0	19	0
sp|Q04C15|RL	23 kDa	11	17	0	74	0	0	0	17	0
tr|C2KDV3|C2KD	43 kDa	23	0	0	0	0	0	0	100	93
tr|C2KH08|C2K	16 kDa	22	11	11	0	1	0	0	4	0
tr|E4SKY6|E4SI	16 kDa	20	13	11	0	0	0	0	4	0
tr|B3WAJ9|B3V	15 kDa	18	9	50	4	0	0	0	3	2
tr|A8YW12|A8	44 kDa	22	0	0	0	0	194	0	16	21
tr|F0NRL3|F0N	44 kDa	14	0	0	0	0	182	0	12	17
tr|D5H0V9|D5H0	13 kDa	21	0	0	0	0	0	0	6	17

sp|Q04C18|RS	18 kDa	21	60	8	102	34	2	11	22	4
tr|D5H2N6|D5	76 kDa	20	23	0	0	2	5	3	13	29
tr|R5YYL2|R5Y	79 kDa	6	82	0	0	3	14	9	3	5
tr|J3ZET6|J3ZE	79 kDa	5	23	0	0	3	52	17	5	7
tr|N2A286|N2A	59 kDa	0	0	0	0	2	10	3	0	0
tr|C2EMG0|C2	80 kDa	0	16	0	0	0	8	0	0	0
tr|C9M2W1|C9	67 kDa	0	23	0	0	3	52	19	0	0
tr|C2HNP9|C2H	79 kDa	0	20	0	0	0	23	8	0	9
tr|D5H0Y4|D5H	30 kDa	20	0	0	0	14	3	7	14	3
tr|C9M1R2|C9I	31 kDa	9	0	0	2	30	6	22	4	1
tr|E4SKH9|E4S	30 kDa	0	0	0	0	9	0	0	0	0
tr|D5H194|D5H	40 kDa	20	0	0	0	0	0	0	1	0
tr|E4SLF0|E4SLF	28 kDa	19	413	12	0	1	0	0	0	1
tr|C2KH06|C2K	18 kDa	17	15	5	0	0	0	0	9	0
tr|D4YTA2|D4Y	18 kDa	14	7	5	0	0	0	0	7	0
sp|Q035A0|RS	18 kDa	6	0	13	0	0	0	0	2	0
sp|A8YVT1|RL1	13 kDa	17	6	1	17	0	0	0	9	0
sp|Q049S3|RL1	13 kDa	8	2	1	32	0	0	0	6	0
sp|A8YXL0|RL22_	13 kDa	17	26	0	3	2	0	0	19	3
tr|C7XM27|C7	48 kDa	16	1	0	0	4	10	9	66	3
tr|F6CBN0|F6C	48 kDa	14	1	0	0	4	10	8	59	3
tr|A8YU36|A8Y	48 kDa	9	1	0	0	8	18	16	42	1
tr|C2HN29|C2H	48 kDa	9	3	0	0	5	11	9	39	0
tr|C2HM25|C2HN	60 kDa	16	33	0	0	11	9	7	18	32
tr|D5GZX0|D5G	96 kDa	16	2	0	3	1	0	4	0	0
sp|Q10730|AN	96 kDa	0	0	0	0	1	0	22	0	0
tr|E4SNC5|E4S	96 kDa	0	4	0	0	0	0	11	0	0
tr|F3MN74|F3I	96 kDa	0	0	0	0	0	0	23	0	0
tr|D5H0V7|D5I	52 kDa	15	0	0	0	22	20	30	247	2
tr|C7XKV4|C7X	52 kDa	10	0	0	0	35	30	45	234	2
tr|C9M1N6|C9	52 kDa	5	0	0	0	112	264	253	43	0
tr|A8YX29|A8Y	52 kDa	5	0	0	0	95	253	243	45	0
tr|C2HLZ4|C2H	52 kDa	4	0	0	0	13	23	31	83	0
tr|G6F4C9|G6F	66 kDa	14	9	0	104	8	22	31	47	1
tr|D1YG09|D1Y	65 kDa	0	0	0	0	0	14	22	21	0
tr|F0K2X6|F0K	46 kDa	13	14	0	238	11	0	23	2	15
tr|K0NFE8|K0N	47 kDa	0	15	0	120	16	0	30	0	20
sp|A8YXK1|RS1	15 kDa	14	22	14	5	0	0	0	10	0
sp|Q04C19|RS	15 kDa	11	13	14	6	0	0	0	9	0
sp|B3WAM4|R	15 kDa	7	7	42	1	0	0	0	4	1
sp|A8YXL2|RL1	16 kDa	14	15	0	8	0	0	4	4	0
tr|C4VM78|C4	16 kDa	11	12	0	0	0	0	0	0	0
sp|Q04C08|RL	16 kDa	6	0	0	31	0	0	1	0	0
sp|B3WDV5|RS4_	23 kDa	14	8	90	10	2	0	2	2	1
tr|C7XKV9|C7X	60 kDa	13	0	0	0	1	4	11	74	12
tr|A8YX36|A8Y	59 kDa	6	0	0	0	2	8	22	32	8
tr|I7LFC8|I7LF	60 kDa	3	0	0	0	0	0	2	12	3
tr|E4SK81|E4SI	60 kDa	1	0	0	0	0	0	0	17	3
tr|G6F3U1|G6F	62 kDa	0	0	0	41	2	0	0	0	0
tr|A4UAC4|A4U	60 kDa	0	0	0	0	0	0	5	17	0
tr|F6CFM6|F6C	61 kDa	0	0	0	0	0	0	0	17	0
tr|F0JZE1|F0JZI	26 kDa	0	0	0	10	0	0	0	0	0
tr|R1B2Q0|R1E	60 kDa	0	0	0	37	0	0	0	1	0
tr|F6CFM5|F6C	60 kDa	0	0	0	0	0	0	0	35	0
tr|E3R5Z3|E3R	58 kDa	0	0	0	0	0	0	0	72	0
tr|C2KD34|C2K	76 kDa	13	0	0	0	0	0	2	6	0
tr|A8YWB1|A8	74 kDa	2	0	0	0	0	0	11	0	0
tr|G6F6B1|G6F	74 kDa	0	0	0	19	0	0	0	0	0
tr|F0HU57|F0H	80 kDa	0	0	0	12	0	0	0	0	0
tr|C2KGJ9|C2K	34 kDa	13	3	0	0	17	0	11	0	3
tr|F6CB50|F6C	34 kDa	6	0	0	0	10	0	5	0	0
tr|E4SIG1|E4SI	34 kDa	5	4	0	0	7	0	8	0	0
tr|F0K1T7|F0K	63 kDa	13	27	0	226	22	0	12	5	5
tr|R1CXN4|R1C	63 kDa	11	20	0	192	0	0	6	0	5
tr|C4VMT3|C4	63 kDa	5	8	3	30	0	0	3	0	0
tr|I7KGT9|I7KG	63 kDa	5	8	2	66	0	0	0	3	3
tr|C7XU23|C7X	52 kDa	0	0	0	12	0	0	0	0	0
tr|C4VQ71|C4V	64 kDa	0	16	2	60	0	0	0	0	0
tr|C8PAQ7|C8F	63 kDa	0	8	0	40	0	0	0	0	0
tr|G6ES43|G6ES4	9 kDa	13	5	0	49	16	0	1	2	0
tr|C2KGF5|C2K	60 kDa	12	0	0	0	0	1	0	15	1
tr|A8YWM5|A8	60 kDa	11	0	0	0	0	0	0	12	0
tr|C2KGZ1|C2K	12 kDa	12	1	0	2	0	0	0	12	3
tr|C2KEW1|C2I	50 kDa	12	0	0	0	0	0	0	0	0
sp|Q5FJK5|RS1	10 kDa	10	13	0	0	0	0	0	8	1
sp|A8YVT4|RS1	10 kDa	7	11	0	0	0	0	0	4	0
sp|B3WEU4|RS	11 kDa	0	0	50	0	0	0	0	0	0
tr|D5GZD1|D5G	61 kDa	11	27	0	0	0	0	0	8	0
tr|C7XJB1|C7X	61 kDa	10	27	0	0	0	0	0	10	0
tr|C2KEG9|C2K	61 kDa	8	35	0	5	0	0	0	12	0
tr|N2AN03|N2A	36 kDa	6	22	0	0	0	0	0	3	0
tr|E4SMF4|E4S	61 kDa	3	57	0	0	0	0	0	5	0
tr|D5GZC7|D5GZ	20 kDa	11	0	0	0	0	0	0	15	7
tr|C2KET7|C2K	34 kDa	11	0	0	4	0	0	0	5	2
tr|D5GYT0|D5GY	28 kDa	11	0	0	0	0	2	1	6	0
tr|F0TIK1|F0TIK1_	20 kDa	11	10	0	0	4	0	0	12	8
tr|D5H0U5|D5I	45 kDa	10	0	0	0	6	0	0	84	40
tr|C7XKU2|C7X	45 kDa	9	0	0	0	6	0	0	88	37
tr|C2KGN9|C2K	80 kDa	10	0	0	0	18	2	5	2	0
tr|C2EMZ3|C2E	81 kDa	10	0	0	0	0	0	0	0	0
tr|F0NTA0|F0N	81 kDa	7	0	0	0	20	3	6	0	0
tr|C9LZZ1|C9LZ	81 kDa	7	0	0	0	20	3	6	0	0
tr|F6CB25|F6C	81 kDa	6	0	0	0	13	2	0	0	0
tr|I7K224|I7K2	80 kDa	4	0	0	0	14	0	0	1	0
tr|F0K2V7|F0K	82 kDa	0	0	0	16	0	0	0	0	0
tr|D5GZ67|D5G	92 kDa	10	0	0	1	0	0	0	0	0

tr|C2KC61|C2K	50 kDa	9	61	0	0	0	0	24	2	18
tr|E4SI62|E4SI	49 kDa	7	88	0	1	6	0	28	2	10
tr|C9M0F7|C9	49 kDa	6	60	0	0	9	0	48	2	9
tr|F6CEC0|F6C	49 kDa	6	38	0	2	0	0	31	0	9
tr|J4BV42|J4B	50 kDa	6	60	0	0	0	0	36	0	9
tr|C2EMK3|C2	49 kDa	0	37	0	0	0	0	28	0	8
tr|C2KD24|C2K	35 kDa	9	0	0	0	0	0	0	35	9
tr|C7Y6B3|C7Y	35 kDa	7	0	0	0	0	0	0	44	10
tr|A4ZGW3|A4	28 kDa	5	0	0	3	0	0	12	0	4
tr|E4SJM6|E4S	34 kDa	8	469	1	0	0	8	1	0	0
tr|S5E507|S5E	17 kDa	4	130	0	0	0	7	0	0	0
tr|A8YWK5|A8	34 kDa	0	344	1	0	0	12	0	0	0
tr|C2KBR7|C2K	46 kDa	8	0	5	34	0	0	10	0	1
sp|A8YUE3|PG	43 kDa	3	0	4	34	0	0	18	0	0
sp|O32756|PG	43 kDa	2	0	4	165	0	0	3	0	0
tr|K0NDC7|K0	43 kDa	2	0	4	103	0	0	2	0	0
sp|B3WCW5|P	42 kDa	1	0	18	20	0	0	2	0	0
tr|C2EM36|C2	46 kDa	1	0	4	25	0	0	5	0	0
tr|J3JAY7|J3JA	43 kDa	0	0	0	37	0	0	0	0	0
tr|S6BQV5|S6B	42 kDa	0	0	14	0	0	0	0	0	0
sp|Q048Z3|SY	97 kDa	3	1	0	110	1	0	1	0	0
sp|A8YTB1|RL1	17 kDa	8	1	0	0	0	3	1	27	1
tr|E4SL00|E4SL	16 kDa	4	3	0	0	0	0	1	12	1
tr|C2KH14|C2K	35 kDa	7	13	4	11	11	0	8	0	4
sp|A8YXN0|RP	35 kDa	5	12	4	11	14	0	10	3	3
sp|Q04BY9|RP	35 kDa	4	13	4	87	6	0	7	3	3
tr|M4NK63|M	26 kDa	0	0	0	10	0	0	0	0	0
tr|C2KB43|C2K	66 kDa	7	0	0	29	1	0	0	0	0
tr|G6EVW7|G6	66 kDa	2	0	0	82	0	0	0	0	0
tr|D6S505|D6S	66 kDa	1	0	0	12	1	0	0	0	0
tr|D4FEF2|D4F	13 kDa	7	0	0	0	0	0	0	31	7
tr|F0K101|F0K	27 kDa	3	0	0	33	0	6	3	3	1
tr|F0JZT5|F0JZ	54 kDa	0	1	0	46	0	0	0	0	0
tr|C2KD26|C2K	50 kDa	7	0	0	0	0	0	30	2	4
tr|A8YWA4|A8	51 kDa	4	0	0	10	1	0	44	3	3
tr|C8PAE9|C8P	50 kDa	4	0	0	24	0	0	11	1	0
tr|I7L654|I7L6	50 kDa	4	0	0	12	0	0	13	1	0
sp|O32757|TPIS_	27 kDa	7	0	0	29	0	0	2	0	2
tr|D5GXV7|D5	50 kDa	6	8	0	8	15	0	0	0	5
tr|C9LZ15|C9L	50 kDa	4	9	0	8	24	0	0	0	4
tr|E4SJH5|E4SJ	50 kDa	3	23	0	8	20	0	0	0	3
tr|K1M4D1|K1	55 kDa	6	4	0	0	0	0	0	38	3
tr|D5GYZ1|D5	55 kDa	6	4	0	0	0	0	0	39	1
tr|D5GZK5|D5	36 kDa	4	0	0	0	0	0	0	7	10
sp|A8YXL5|RL1	13 kDa	6	3	15	3	2	0	3	2	0
sp|B3WAK7|RL	13 kDa	4	3	26	4	2	0	2	1	0
sp|Q04C05|RL	13 kDa	4	3	17	11	2	0	2	1	0
tr|I7IVD2|I7IV	13 kDa	0	0	17	4	2	0	0	1	0
tr|D4YSL4|D4Y	33 kDa	3	10	0	4	0	0	2	0	4
tr|E4SM21|E4S	33 kDa	0	20	0	0	0	0	0	0	0
tr|C2HLY2|C2HL	45 kDa	6	0	0	0	0	0	0	0	0
sp|B3WEL7|RS	7 kDa	4	13	106	2	0	0	0	6	1
sp|Q88VR5|RS	8 kDa	2	3	70	0	0	0	0	3	0
sp|A8YVN1|RS21	7 kDa	6	12	26	0	0	0	0	14	1
sp|Q04C10|RL22_	13 kDa	6	7	0	15	2	0	0	4	2
tr|C2KFW1|C2	38 kDa	5	34	2	0	1	0	1	0	8
tr|R5YS99|R5Y	37 kDa	4	53	2	0	1	0	1	0	3
sp|A8YVR8|EF	38 kDa	3	29	0	0	1	0	6	0	2
tr|D8FMC0|D8	51 kDa	0	0	0	43	0	0	0	0	0
tr|D5GYE1|D5	34 kDa	5	0	0	0	0	0	0	0	10
tr|C7XIX0|C7XI	33 kDa	4	0	0	0	0	0	0	0	11
tr|D5GZG0|D5GZ	92 kDa	5	0	0	0	0	0	12	0	0
tr|C2KFS1|C2KFS	41 kDa	5	0	0	0	0	0	0	0	0
tr|E4SIG8|E4SI	27 kDa	0	17	0	0	0	3	1	0	0
tr|C2EN32|C2E	19 kDa	5	16	0	0	0	0	0	2	1
tr|E4SIN4|E4SI	20 kDa	2	17	0	0	0	0	0	0	0
sp|A8YXN1|RL17_	14 kDa	5	10	0	0	0	0	0	17	1
tr|E4SM52|E4SM	40 kDa	5	13	0	0	0	0	0	2	0
tr|A4ZH27|A4Z	41 kDa	5	8	0	0	50	70	69	4	4
tr|F3MPK9|F3	136 kDa	4	5	0	34	18	0	0	0	0
sp|Q04C22|RP	136 kDa	3	1	1	196	4	0	0	0	0
tr|N1ZQV0|N1	136 kDa	3	5	1	44	13	0	0	0	0
tr|C6VN61|C6	135 kDa	1	0	7	24	0	0	0	0	0
sp|B3WAM8|R	134 kDa	1	1	24	20	3	0	0	0	0
tr|I7IZJ9|I7IZJ9_	135 kDa	0	5	0	41	11	0	0	0	0
tr|G9ICP9|G9I	86 kDa	0	0	11	0	0	0	0	0	0
tr|E0YTW7|E0	12 kDa	0	0	0	28	0	0	0	0	0
tr|F0K366|F0K	59 kDa	1	2	0	49	0	0	2	0	0
tr|E4SKC4|E4SI	60 kDa	0	12	0	0	0	0	3	0	0
tr|C5F561|C5F	68 kDa	3	0	13	0	0	0	1	0	0
sp|Q04BV6|GA	54 kDa	3	0	0	17	0	0	3	0	0
tr|D8FNZ3|D8	35 kDa	3	0	1	20	0	0	0	0	0
sp|A8YVX6|RL27_	10 kDa	4	0	0	0	0	0	0	18	0
tr|C2KGD7|C2KG	27 kDa	4	0	0	0	0	0	0	2	0
tr|C8PD72|C8PD	66 kDa	4	0	0	0	0	14	18	2	0
tr|A8YWT3|A8	25 kDa	4	12	0	0	0	0	1	0	0
tr|E4SM23|E4S	25 kDa	0	33	0	0	0	0	0	0	0
tr|F0JZZ9|F0JZZ9_	14 kDa	4	0	0	19	0	0	0	0	0
tr|F3MN05|F3M	20 kDa	4	0	0	0	0	0	5	4	2
tr|N2A9R3|N2	69 kDa	3	4	0	18	0	0	2	0	0
tr|G6ETM4|G6	69 kDa	1	0	0	37	0	0	0	0	0
tr|R1CVP3|R1C	69 kDa	1	0	0	31	0	0	0	0	0
tr|F3N086|F3N	62 kDa	0	0	15	0	0	0	0	0	0
tr|B3WE23|B3	68 kDa	0	0	25	1	0	0	0	0	0
sp|Q04BF6|GL	49 kDa	0	0	0	41	0	0	0	0	0
sp|A8YVR3|SY	63 kDa	3	9	0	0	89	0	21	2	1

tr|E4SJZ9|E4SJ	63 kDa	3	11	0	0	30	0	12	2	1
tr|I7JU69|I7JU	63 kDa	0	3	0	5	30	0	7	1	0
tr|D7V3Y9|D7V	63 kDa	0	0	0	5	32	0	6	0	0
tr|C2KF80|C2K	45 kDa	3	0	0	0	0	0	2	171	30
sp|B3WAL8|RS	12 kDa	0	2	37	0	0	0	0	0	0
sp|A5VLK6|RS1	12 kDa	0	0	24	0	0	0	0	0	0
sp|A8YVH1|PP	34 kDa	3	0	0	0	0	0	19	0	1
tr|E4SKF0|E4SKF	11 kDa	3	17	0	1	2	0	0	3	0
tr|C2EQL7|C2EQI	61 kDa	3	6	0	0	0	5	4	10	11
sp|Q049V5|IF2	91 kDa	3	3	0	107	1	2	0	2	0
tr|K0NQ12|K0I	91 kDa	0	3	0	30	0	0	0	0	0
tr|F6CFQ3|F6CFC	60 kDa	3	1	0	0	21	50	0	8	11
tr|E4SW47|E4SW	33 kDa	3	11	0	14	3	0	2	0	4
tr|B5QPS8|B5QP	7 kDa	3	0	16	0	0	0	0	0	0
tr|C5F3S3|C5F	67 kDa	3	0	40	0	0	0	0	0	0
tr|S6C884|S6C	67 kDa	0	0	16	0	0	0	0	0	0
tr|F0K3A3|F0K	53 kDa	3	0	0	58	0	0	0	2	0
tr|F0K1J0|F0K1	101 kDa	0	0	0	34	0	0	0	0	0
tr|C5G2X8|C5G	102 kDa	0	0	0	11	0	0	0	0	0
tr|K0NGD5|K0I	101 kDa	0	0	0	26	0	0	0	0	0
tr|D5H154|D5I	136 kDa	2	2	4	43	1	0	0	2	1
tr|F3MPL0|F3N	136 kDa	2	2	4	50	2	0	1	2	0
sp|Q04C21|RP	136 kDa	1	0	4	203	1	0	0	1	0
tr|R9G902|R9G	136 kDa	1	0	11	0	0	0	0	0	0
sp|Q034X1|RP	136 kDa	1	0	28	28	0	0	0	1	0
tr|J2ZQN4|J2Z	135 kDa	0	0	6	22	0	0	0	0	0
tr|K5ENX7|K5E	135 kDa	0	0	6	23	0	0	0	2	0
tr|C2D3G6|C2D	136 kDa	0	0	6	26	0	0	0	0	0
sp|Q03PV0|RP	136 kDa	0	0	3	22	0	0	0	0	0
tr|U2H4H6|U2	34 kDa	0	0	1	8	0	0	0	0	0
tr|I7LDR6|I7LD	136 kDa	0	2	4	54	1	0	1	0	0
tr|N1ZW37|N1	137 kDa	0	0	8	21	1	0	0	0	0
tr|G9ICQ0|G9I	129 kDa	0	0	16	35	0	0	0	1	0
tr|G6CE90|G6C	136 kDa	0	0	9	33	0	0	0	0	0
tr|C7XKU9|C7X	26 kDa	2	11	0	16	5	0	21	1	6
tr|D8FQZ9|D8F	26 kDa	1	9	2	74	0	0	7	0	4
tr|F3MNR7|F3I	27 kDa	0	13	2	16	9	0	25	1	4
tr|C2EH56|C2E	26 kDa	0	0	1	12	0	0	0	0	0
tr|C2ETX9|C2E	26 kDa	0	0	2	0	0	0	5	0	0
tr|B2GF82|B2G	26 kDa	0	0	0	0	0	0	0	0	0
tr|M5J812|M5	26 kDa	0	0	2	16	0	0	0	0	0
tr|S2MVG4|S2I	9 kDa	0	0	0	4	0	0	0	0	0
tr|F2LYZ4|F2LY	62 kDa	2	2	0	19	0	0	0	0	0
tr|F0K1M3|F0K	62 kDa	1	1	0	69	0	0	0	1	0
tr|C2KGK1|C2K	34 kDa	2	5	0	0	6	0	2	0	1
tr|A8YV24|A8Y	34 kDa	1	5	0	0	10	0	2	0	0
sp|O84913|PEI	41 kDa	1	0	0	0	9	0	12	0	0
tr|F0K0D8|F0K	103 kDa	0	0	0	54	0	0	0	0	0
tr|D8FLW7|D8I	109 kDa	0	0	0	48	0	0	0	0	0
sp|Q04C01|RS	14 kDa	0	0	0	21	0	0	0	0	0
sp|B3WAK4|RS	15 kDa	0	0	68	1	0	0	0	0	0
sp|Q5FIY7|SYFI	89 kDa	1	0	0	0	15	0	3	0	0
tr|C9M4A8|C9I	89 kDa	0	0	0	0	37	0	16	0	0
tr|F0HX11|F0H	58 kDa	2	5	0	50	1	0	3	0	0
tr|F3MPK5|F3N	60 kDa	0	7	0	9	10	0	8	0	0
tr|I7K035|I7K0	58 kDa	0	8	0	17	3	0	2	0	0
tr|J4BTA1|J4BT	60 kDa	0	10	0	0	0	0	0	0	0
sp|Q88XW3|RL	5 kDa	0	3	11	7	0	0	0	0	0
sp|Q04BZ2|RL	4 kDa	0	2	0	11	0	0	0	0	0
tr|F0JZF8|F0JZI	40 kDa	0	0	0	37	0	0	2	0	0
tr|C9M1P3|C9I	40 kDa	0	0	0	3	0	0	24	0	2
tr|C2KDN2|C2K	40 kDa	0	0	0	0	0	0	9	0	11
tr|G6F6P4|G6F	62 kDa	2	5	0	66	0	0	0	2	0
sp|B3WEW2|RL2	7 kDa	2	2	213	0	0	0	0	1	0
tr|E4SLC3|E4SLC	35 kDa	2	10	0	0	3	5	3	7	1
tr|D8FNG9|D8I	21 kDa	2	1	0	7	2	0	1	0	0
tr|F0K3H7|F0K	50 kDa	2	0	1	301	0	0	4	0	0
tr|R1AZ75|R1A	50 kDa	0	0	1	255	0	0	0	0	0
tr|S2KRL8|S2KI	50 kDa	0	0	1	271	0	0	0	0	0
sp|B3WET9|RL	13 kDa	2	0	33	2	0	0	0	0	0
sp|Q03RU8|RL	14 kDa	0	0	10	0	0	0	0	0	0
tr|G2KTK6|G2K	14 kDa	0	0	10	0	0	0	0	0	0
tr|C0YWK9|C0	13 kDa	0	0	10	0	0	0	0	0	0
sp|B3WAK5|RL5_	20 kDa	2	8	15	7	6	0	0	2	3
tr|D5GYW3|D5	73 kDa	1	6	1	15	15	0	4	0	6
tr|F0K3K7|F0K	73 kDa	0	6	1	87	10	0	4	0	2
tr|C9M4D5|C9I	74 kDa	0	9	1	13	34	0	12	0	5
tr|D8GHG5|D8	75 kDa	0	5	5	16	10	0	4	0	0
tr|E4SLN3|E4SI	74 kDa	0	21	1	14	22	0	6	0	5
tr|I7J152|I7J15	73 kDa	0	6	1	12	14	0	3	0	0
tr|F6CDN2|F6C	73 kDa	0	9	0	0	15	0	0	0	3
tr|S2RUS5|S2R	30 kDa	0	0	3	0	10	0	0	0	0
tr|F6CCK8|F6C	42 kDa	1	36	0	0	7	10	2	7	0
tr|A8YWH9|A8	14 kDa	1	36	0	0	0	0	0	0	0
tr|C9M1Y4|C9I	42 kDa	0	0	0	0	0	10	0	72	1
tr|C2KEL5|C2KI	42 kDa	0	0	0	0	6	22	2	687	5
tr|A8YWH8|A8	21 kDa	0	0	0	0	7	10	0	6	0
tr|C2KBD5|C2K	36 kDa	1	0	0	0	0	0	0	16	0
tr|G6ETN5|G6E	68 kDa	0	0	0	68	0	0	0	0	0
tr|F2M1R8|F2I	8 kDa	1	3	0	11	0	0	0	0	0
tr|F6CDN6|F6C	8 kDa	0	3	0	14	0	0	0	0	0
tr|C2KC11|C2K	35 kDa	1	0	0	14	0	0	0	0	0
tr|F0K1J4|F0K1	35 kDa	0	0	0	22	0	0	0	0	0
tr|E1NEP4|E1N	35 kDa	0	0	0	12	0	0	0	0	0
tr|E4SK47|E4SI	48 kDa	1	0	0	0	0	4	0	23	0
tr|K1M441|K1I	46 kDa	1	12	0	0	0	0	0	517	5

<table>
<tr><td>tr|C7XJ17|C7XJ</td><td>45 kDa</td><td>1</td><td>12</td><td>0</td><td>0</td><td>0</td><td>0</td><td>0</td><td>620</td><td>2</td></tr>
<tr><td>sp|Q04B65|SYI</td><td>106 kDa</td><td>0</td><td>0</td><td>0</td><td>85</td><td>0</td><td>0</td><td>0</td><td>0</td><td>0</td></tr>
<tr><td>tr|K6RHV8|K6F</td><td>105 kDa</td><td>0</td><td>0</td><td>13</td><td>0</td><td>0</td><td>0</td><td>0</td><td>0</td><td>0</td></tr>
<tr><td>sp|A8YUK1|ATI</td><td>52 kDa</td><td>1</td><td>10</td><td>1</td><td>41</td><td>6</td><td>0</td><td>32</td><td>2</td><td>7</td></tr>
<tr><td>tr|Q6RX76|Q6F</td><td>41 kDa</td><td>1</td><td>7</td><td>1</td><td>27</td><td>5</td><td>0</td><td>19</td><td>0</td><td>6</td></tr>
<tr><td>sp|Q04BA3|AT</td><td>52 kDa</td><td>0</td><td>9</td><td>1</td><td>137</td><td>6</td><td>0</td><td>13</td><td>2</td><td>3</td></tr>
<tr><td>sp|Q1WUC6|A</td><td>51 kDa</td><td>0</td><td>9</td><td>1</td><td>37</td><td>4</td><td>0</td><td>7</td><td>0</td><td>0</td></tr>
<tr><td>tr|G6F6T7|G6F</td><td>54 kDa</td><td>0</td><td>9</td><td>0</td><td>125</td><td>6</td><td>0</td><td>13</td><td>0</td><td>0</td></tr>
<tr><td>tr|I7KI41|I7KI4</td><td>52 kDa</td><td>0</td><td>10</td><td>0</td><td>0</td><td>0</td><td>0</td><td>0</td><td>0</td><td>6</td></tr>
<tr><td>tr|C9M4G5|C9</td><td>178 kDa</td><td>0</td><td>0</td><td>0</td><td>0</td><td>0</td><td>0</td><td>138</td><td>1</td><td>0</td></tr>
<tr><td>tr|G8DA68|G8I</td><td>179 kDa</td><td>0</td><td>0</td><td>0</td><td>0</td><td>0</td><td>0</td><td>124</td><td>0</td><td>0</td></tr>
<tr><td>tr|F6CCI9|F6C</td><td>79 kDa</td><td>1</td><td>0</td><td>0</td><td>0</td><td>3</td><td>0</td><td>107</td><td>3</td><td>0</td></tr>
<tr><td>tr|A4ZH30|A4Z</td><td>79 kDa</td><td>0</td><td>0</td><td>0</td><td>0</td><td>3</td><td>0</td><td>223</td><td>7</td><td>0</td></tr>
<tr><td>tr|D0DZE5|D0D</td><td>76 kDa</td><td>0</td><td>0</td><td>0</td><td>0</td><td>0</td><td>0</td><td>33</td><td>0</td><td>0</td></tr>
<tr><td>tr|F2M0D7|F2I</td><td>44 kDa</td><td>1</td><td>7</td><td>0</td><td>0</td><td>9</td><td>0</td><td>10</td><td>0</td><td>1</td></tr>
<tr><td>tr|C9LZ91|C9LZ</td><td>41 kDa</td><td>0</td><td>5</td><td>0</td><td>0</td><td>15</td><td>0</td><td>13</td><td>0</td><td>1</td></tr>
<tr><td>tr|C9M3E6|C9M</td><td>40 kDa</td><td>1</td><td>0</td><td>0</td><td>0</td><td>15</td><td>2</td><td>21</td><td>0</td><td>1</td></tr>
<tr><td>tr|C2KGD3|C2K</td><td>44 kDa</td><td>1</td><td>0</td><td>0</td><td>0</td><td>0</td><td>0</td><td>0</td><td>12</td><td>0</td></tr>
<tr><td>tr|A8YV00|A8YV</td><td>70 kDa</td><td>1</td><td>1</td><td>0</td><td>1</td><td>0</td><td>0</td><td>11</td><td>0</td><td>0</td></tr>
<tr><td>tr|S2QGB6|S2C</td><td>37 kDa</td><td>1</td><td>0</td><td>14</td><td>0</td><td>0</td><td>0</td><td>0</td><td>0</td><td>0</td></tr>
<tr><td>tr|S2NWD1|S2</td><td>98 kDa</td><td>0</td><td>0</td><td>24</td><td>0</td><td>0</td><td>0</td><td>0</td><td>0</td><td>0</td></tr>
<tr><td>tr|C7TJQ0|C7T</td><td>102 kDa</td><td>0</td><td>3</td><td>15</td><td>0</td><td>0</td><td>0</td><td>0</td><td>0</td><td>0</td></tr>
<tr><td>sp|Q04BB3|RF</td><td>41 kDa</td><td>0</td><td>1</td><td>0</td><td>17</td><td>2</td><td>0</td><td>3</td><td>0</td><td>0</td></tr>
<tr><td>tr|E4SJB8|E4SJB8</td><td>41 kDa</td><td>1</td><td>38</td><td>0</td><td>0</td><td>0</td><td>0</td><td>0</td><td>0</td><td>0</td></tr>
<tr><td>tr|G6F6V1|G6F</td><td>45 kDa</td><td>1</td><td>5</td><td>0</td><td>57</td><td>5</td><td>0</td><td>0</td><td>0</td><td>0</td></tr>
<tr><td>tr|K0NQU8|K0I</td><td>45 kDa</td><td>0</td><td>5</td><td>0</td><td>45</td><td>5</td><td>0</td><td>0</td><td>0</td><td>0</td></tr>
<tr><td>tr|F0K100|F0K10</td><td>95 kDa</td><td>1</td><td>0</td><td>0</td><td>16</td><td>0</td><td>0</td><td>0</td><td>0</td><td>0</td></tr>
<tr><td>sp|Q048T9|RL1_</td><td>25 kDa</td><td>1</td><td>0</td><td>0</td><td>49</td><td>0</td><td>0</td><td>0</td><td>0</td><td>0</td></tr>
<tr><td>tr|A8YWA2|A8</td><td>36 kDa</td><td>1</td><td>2</td><td>0</td><td>0</td><td>10</td><td>33</td><td>23</td><td>6</td><td>1</td></tr>
<tr><td>tr|D8FNZ4|D8F</td><td>78 kDa</td><td>0</td><td>1</td><td>0</td><td>62</td><td>0</td><td>0</td><td>0</td><td>0</td><td>0</td></tr>
<tr><td>tr|Q1G9W9|Q1</td><td>79 kDa</td><td>0</td><td>1</td><td>0</td><td>63</td><td>0</td><td>0</td><td>0</td><td>0</td><td>0</td></tr>
<tr><td>tr|S2K8Q1|S2K</td><td>78 kDa</td><td>0</td><td>1</td><td>0</td><td>62</td><td>0</td><td>0</td><td>0</td><td>0</td><td>0</td></tr>
<tr><td>tr|K0NTQ3|K0N</td><td>77 kDa</td><td>0</td><td>0</td><td>0</td><td>32</td><td>0</td><td>0</td><td>0</td><td>0</td><td>0</td></tr>
<tr><td>tr|B3WEA5|B3</td><td>78 kDa</td><td>0</td><td>0</td><td>13</td><td>0</td><td>0</td><td>0</td><td>0</td><td>0</td><td>0</td></tr>
<tr><td>tr|F0K0Q0|F0K</td><td>91 kDa</td><td>0</td><td>0</td><td>0</td><td>12</td><td>0</td><td>0</td><td>0</td><td>0</td><td>0</td></tr>
<tr><td>tr|E4SX83|E4S</td><td>91 kDa</td><td>0</td><td>0</td><td>0</td><td>10</td><td>0</td><td>0</td><td>0</td><td>0</td><td>0</td></tr>
<tr><td>tr|F0JZU5|F0JZ</td><td>80 kDa</td><td>0</td><td>0</td><td>0</td><td>19</td><td>0</td><td>0</td><td>0</td><td>0</td><td>0</td></tr>
<tr><td>tr|E4SMW8|E4</td><td>32 kDa</td><td>0</td><td>10</td><td>0</td><td>0</td><td>0</td><td>0</td><td>0</td><td>0</td><td>0</td></tr>
<tr><td>tr|F0HTF0|F0H</td><td>211 kDa</td><td>0</td><td>0</td><td>0</td><td>72</td><td>0</td><td>0</td><td>0</td><td>0</td><td>0</td></tr>
<tr><td>tr|Q48545|Q48</td><td>212 kDa</td><td>0</td><td>0</td><td>0</td><td>80</td><td>0</td><td>0</td><td>0</td><td>0</td><td>0</td></tr>
<tr><td>tr|Q04A74|Q0</td><td>197 kDa</td><td>0</td><td>0</td><td>0</td><td>82</td><td>0</td><td>0</td><td>0</td><td>0</td><td>0</td></tr>
<tr><td>tr|F0JZJ7|F0JZJ</td><td>75 kDa</td><td>0</td><td>0</td><td>0</td><td>45</td><td>0</td><td>0</td><td>0</td><td>0</td><td>0</td></tr>
<tr><td>tr|J4BUL7|J4BUL</td><td>91 kDa</td><td>0</td><td>0</td><td>0</td><td>0</td><td>0</td><td>0</td><td>63</td><td>1</td><td>0</td></tr>
<tr><td>tr|D8FP97|D8F</td><td>33 kDa</td><td>0</td><td>0</td><td>0</td><td>16</td><td>0</td><td>0</td><td>0</td><td>0</td><td>0</td></tr>
<tr><td>sp|Q04B11|OB</td><td>48 kDa</td><td>0</td><td>0</td><td>0</td><td>19</td><td>0</td><td>0</td><td>0</td><td>0</td><td>0</td></tr>
<tr><td>sp|B3WF43|RL20</td><td>13 kDa</td><td>0</td><td>0</td><td>54</td><td>0</td><td>0</td><td>0</td><td>0</td><td>0</td><td>0</td></tr>
<tr><td>tr|F0K1P0|F0K</td><td>49 kDa</td><td>0</td><td>0</td><td>0</td><td>244</td><td>0</td><td>0</td><td>0</td><td>0</td><td>0</td></tr>
<tr><td>tr|G6F6N7|G6F</td><td>49 kDa</td><td>0</td><td>0</td><td>0</td><td>231</td><td>0</td><td>0</td><td>0</td><td>0</td><td>0</td></tr>
<tr><td>tr|K0NQR3|K0N</td><td>49 kDa</td><td>0</td><td>0</td><td>0</td><td>109</td><td>0</td><td>0</td><td>0</td><td>0</td><td>0</td></tr>
<tr><td>tr|M5J752|M5</td><td>49 kDa</td><td>0</td><td>0</td><td>0</td><td>11</td><td>0</td><td>0</td><td>0</td><td>0</td><td>0</td></tr>
<tr><td>tr|D8FR00|D8F</td><td>47 kDa</td><td>0</td><td>0</td><td>0</td><td>13</td><td>0</td><td>0</td><td>0</td><td>0</td><td>0</td></tr>
<tr><td>tr|E4SVW7|E4S</td><td>48 kDa</td><td>0</td><td>0</td><td>0</td><td>11</td><td>0</td><td>0</td><td>0</td><td>0</td><td>0</td></tr>
<tr><td>tr|F0K1M2|F0K1</td><td>49 kDa</td><td>0</td><td>0</td><td>0</td><td>48</td><td>0</td><td>0</td><td>0</td><td>0</td><td>0</td></tr>
<tr><td>tr|H3RS95|H3F</td><td>78 kDa</td><td>0</td><td>0</td><td>0</td><td>48</td><td>0</td><td>0</td><td>0</td><td>0</td><td>0</td></tr>
<tr><td>tr|F0K120|F0K</td><td>77 kDa</td><td>0</td><td>0</td><td>0</td><td>176</td><td>0</td><td>0</td><td>0</td><td>0</td><td>0</td></tr>
<tr><td>tr|C2EHW0|C2</td><td>78 kDa</td><td>0</td><td>0</td><td>0</td><td>50</td><td>0</td><td>0</td><td>0</td><td>0</td><td>0</td></tr>
<tr><td>tr|S6BTF7|S6B</td><td>80 kDa</td><td>0</td><td>0</td><td>0</td><td>32</td><td>0</td><td>0</td><td>0</td><td>0</td><td>0</td></tr>
<tr><td>tr|C4MCM0|C4</td><td>45 kDa</td><td>0</td><td>0</td><td>0</td><td>0</td><td>37</td><td>0</td><td>31</td><td>2</td><td>0</td></tr>
<tr><td>tr|C2EQR0|C2E</td><td>45 kDa</td><td>0</td><td>0</td><td>0</td><td>0</td><td>13</td><td>0</td><td>8</td><td>2</td><td>0</td></tr>
<tr><td>sp|Q1G7W2|R</td><td>82 kDa</td><td>0</td><td>0</td><td>0</td><td>64</td><td>0</td><td>0</td><td>0</td><td>0</td><td>0</td></tr>
<tr><td>tr|D4YSZ7|D4Y</td><td>43 kDa</td><td>0</td><td>0</td><td>0</td><td>12</td><td>0</td><td>0</td><td>0</td><td>0</td><td>0</td></tr>
<tr><td>tr|F0JYM0|F0J</td><td>44 kDa</td><td>0</td><td>0</td><td>0</td><td>522</td><td>0</td><td>0</td><td>0</td><td>0</td><td>0</td></tr>
<tr><td>tr|K0NY49|K0N</td><td>44 kDa</td><td>0</td><td>0</td><td>0</td><td>113</td><td>0</td><td>0</td><td>0</td><td>0</td><td>0</td></tr>
<tr><td>tr|E7FRA2|E7F</td><td>10 kDa</td><td>0</td><td>0</td><td>12</td><td>0</td><td>0</td><td>0</td><td>0</td><td>0</td><td>0</td></tr>
<tr><td>tr|B3WE81|B3</td><td>10 kDa</td><td>0</td><td>0</td><td>64</td><td>0</td><td>0</td><td>0</td><td>0</td><td>0</td><td>0</td></tr>
<tr><td>tr|B2GC36|B2G</td><td>10 kDa</td><td>0</td><td>0</td><td>13</td><td>0</td><td>0</td><td>0</td><td>0</td><td>0</td><td>0</td></tr>
<tr><td>sp|Q04BZ8|RS5_</td><td>18 kDa</td><td>0</td><td>0</td><td>0</td><td>17</td><td>0</td><td>0</td><td>0</td><td>0</td><td>0</td></tr>
<tr><td>tr|K0NM87|K0</td><td>59 kDa</td><td>0</td><td>0</td><td>0</td><td>11</td><td>0</td><td>0</td><td>0</td><td>0</td><td>0</td></tr>
<tr><td>sp|Q04BM6|RF</td><td>59 kDa</td><td>0</td><td>0</td><td>0</td><td>18</td><td>1</td><td>0</td><td>0</td><td>0</td><td>0</td></tr>
<tr><td>tr|G8DA69|G8I</td><td>181 kDa</td><td>0</td><td>0</td><td>0</td><td>0</td><td>0</td><td>0</td><td>68</td><td>0</td><td>0</td></tr>
<tr><td>tr|F0TIT7|F0TI</td><td>178 kDa</td><td>0</td><td>0</td><td>0</td><td>0</td><td>0</td><td>0</td><td>52</td><td>0</td><td>0</td></tr>
<tr><td>tr|J3WLB4|J3W</td><td>181 kDa</td><td>0</td><td>0</td><td>0</td><td>0</td><td>0</td><td>0</td><td>59</td><td>0</td><td>0</td></tr>
<tr><td>tr|C2EM91|C2I</td><td>182 kDa</td><td>0</td><td>0</td><td>0</td><td>0</td><td>0</td><td>0</td><td>33</td><td>0</td><td>0</td></tr>
<tr><td>tr|D8FPS7|D8F</td><td>28 kDa</td><td>0</td><td>0</td><td>0</td><td>10</td><td>0</td><td>0</td><td>0</td><td>0</td><td>0</td></tr>
<tr><td>tr|J7LMD3|J7LM</td><td>33 kDa</td><td>0</td><td>0</td><td>0</td><td>0</td><td>13</td><td>2</td><td>4</td><td>0</td><td>0</td></tr>
<tr><td>sp|Q04C14|RL</td><td>23 kDa</td><td>0</td><td>0</td><td>0</td><td>30</td><td>0</td><td>0</td><td>0</td><td>0</td><td>0</td></tr>
<tr><td>sp|B3WAL6|RL</td><td>22 kDa</td><td>0</td><td>0</td><td>24</td><td>0</td><td>0</td><td>0</td><td>0</td><td>0</td><td>0</td></tr>
<tr><td>tr|D8FNX4|D8FN</td><td>27 kDa</td><td>0</td><td>0</td><td>0</td><td>27</td><td>0</td><td>0</td><td>0</td><td>0</td><td>0</td></tr>
<tr><td>tr|K0NB73|K0NB</td><td>19 kDa</td><td>0</td><td>0</td><td>31</td><td>0</td><td>0</td><td>0</td><td>0</td><td>0</td><td>0</td></tr>
<tr><td>tr|E4SJW9|E4SJW</td><td>19 kDa</td><td>0</td><td>20</td><td>0</td><td>0</td><td>0</td><td>0</td><td>0</td><td>0</td><td>0</td></tr>
<tr><td>tr|D5H065|D5H0</td><td>13 kDa</td><td>0</td><td>0</td><td>0</td><td>0</td><td>1</td><td>0</td><td>0</td><td>2</td><td>16</td></tr>
<tr><td>sp|Q04CA4|GU</td><td>57 kDa</td><td>0</td><td>0</td><td>0</td><td>67</td><td>0</td><td>0</td><td>0</td><td>0</td><td>0</td></tr>
<tr><td>tr|C8PBF9|C8PBF</td><td>14 kDa</td><td>0</td><td>0</td><td>10</td><td>0</td><td>0</td><td>0</td><td>0</td><td>3</td><td>0</td></tr>
<tr><td>tr|K1M0K1|K1M</td><td>13 kDa</td><td>0</td><td>0</td><td>0</td><td>0</td><td>0</td><td>0</td><td>0</td><td>0</td><td>23</td></tr>
<tr><td>tr|C9M149|C9I</td><td>32 kDa</td><td>0</td><td>0</td><td>0</td><td>0</td><td>9</td><td>1031</td><td>1216</td><td>214</td><td>4</td></tr>
<tr><td>tr|C0XKV9|C0X</td><td>38 kDa</td><td>0</td><td>0</td><td>0</td><td>0</td><td>8</td><td>874</td><td>1010</td><td>132</td><td>4</td></tr>
<tr><td>tr|J7LF58|J7LF</td><td>37 kDa</td><td>0</td><td>0</td><td>0</td><td>0</td><td>8</td><td>743</td><td>821</td><td>111</td><td>0</td></tr>
<tr><td>tr|R1B2S0|R1B</td><td>37 kDa</td><td>0</td><td>0</td><td>0</td><td>0</td><td>2</td><td>815</td><td>923</td><td>94</td><td>4</td></tr>
<tr><td>tr|A8YWG0|A8</td><td>35 kDa</td><td>0</td><td>38</td><td>0</td><td>0</td><td>401</td><td>65</td><td>167</td><td>220</td><td>74</td></tr>
<tr><td>tr|F0TGF8|F0T</td><td>35 kDa</td><td>0</td><td>15</td><td>0</td><td>0</td><td>257</td><td>30</td><td>66</td><td>68</td><td>77</td></tr>
<tr><td>tr|C2KE41|C2K</td><td>35 kDa</td><td>0</td><td>50</td><td>0</td><td>0</td><td>276</td><td>47</td><td>83</td><td>84</td><td>113</td></tr>
<tr><td>tr|F2M1U8|F2I</td><td>35 kDa</td><td>0</td><td>38</td><td>0</td><td>0</td><td>296</td><td>55</td><td>112</td><td>241</td><td>71</td></tr>
<tr><td>tr|C2HQZ2|C2H</td><td>38 kDa</td><td>0</td><td>0</td><td>0</td><td>0</td><td>8</td><td>0</td><td>0</td><td>0</td><td>0</td></tr>
<tr><td>tr|E4SLY4|E4SL</td><td>35 kDa</td><td>0</td><td>45</td><td>0</td><td>0</td><td>113</td><td>26</td><td>57</td><td>131</td><td>55</td></tr>
<tr><td>tr|F0NSX1|F0N</td><td>218 kDa</td><td>0</td><td>0</td><td>0</td><td>0</td><td>0</td><td>0</td><td>1101</td><td>53</td><td>0</td></tr>
<tr><td>tr|F6CDX9|F6C</td><td>217 kDa</td><td>0</td><td>0</td><td>0</td><td>0</td><td>0</td><td>0</td><td>880</td><td>46</td><td>0</td></tr>
</table>

tr|F3MPF4|F3N	103 kDa	0	0	0	0	0	0	415	26	0
tr|S5DUF9|S5D	31 kDa	0	0	0	0	414	4	389	92	0
tr|F3MPS2|F3N	27 kDa	0	0	0	0	255	4	315	81	0
tr|A8YWK2|A8YV	35 kDa	0	0	0	0	41	889	28	0	0
tr|I7JYF0|I7JYF	44 kDa	0	0	0	0	15	801	8	0	0
tr|E4SJM1|E4S.	44 kDa	0	0	0	0	11	781	7	0	0
tr|C2EP10|C2E	32 kDa	0	0	0	0	0	41	0	0	0
tr|A8YXT2|A8Y	54 kDa	0	0	0	0	1	107	288	34	0
tr|F3MQ94|F3I	12 kDa	0	0	0	0	0	58	178	17	0
tr|J7LME1|J7LN	84 kDa	0	0	0	0	1	107	308	17	0
tr|F6CF66|F6CI	85 kDa	0	0	0	0	0	93	239	16	0
sp|P22294|HVTJ_	38 kDa	0	0	0	0	3	472	7	0	0
tr|C2FCS9|C2F	75 kDa	0	2	496	0	0	0	0	0	0
tr|S6CJQ9|S6C.	74 kDa	0	0	6	0	0	0	0	0	0
tr|C2JZX8|C2JZ	74 kDa	0	0	56	0	0	0	0	0	0
tr|A8YW73|A8	15 kDa	0	3	0	0	129	53	94	9	0
tr|C2ENR7|C2E	15 kDa	0	3	0	0	66	32	70	5	0
tr|A5VM59|A5	14 kDa	0	0	0	0	0	0	0	0	0
tr|F2M071|F2N	15 kDa	0	19	0	0	46	0	40	0	0
tr|C2HKG5|C2H	15 kDa	0	6	0	0	0	0	42	0	0
tr|E4SJ99|E4SJ	15 kDa	0	19	0	0	0	0	0	0	0
tr|F3MP10|F3N	49 kDa	0	6	0	0	227	619	573	101	8
tr|J3ZCG6|J3ZC	49 kDa	0	6	0	0	96	614	509	92	0
tr|C9M1R7|C9I	34 kDa	0	0	0	0	76	55	154	45	0
tr|D5GYW1|D5G	19 kDa	0	0	0	0	0	0	0	299	45
tr|D5H0C3|D5H0	33 kDa	0	0	0	0	0	0	0	337	2
tr|D5GZF2|D5C	30 kDa	0	0	0	0	0	0	0	152	217
tr|D4FH92|D4F	27 kDa	0	0	0	0	0	0	0	0	32
tr|C2EQI4|C2E	31 kDa	0	0	0	0	0	0	0	11	51
tr|F0JYT6|F0JY	56 kDa	0	0	0	277	0	0	0	0	0
tr|D8FPU7|D8F	56 kDa	0	0	0	153	0	0	0	0	0
tr|F0HUB3|F0H	56 kDa	0	0	0	133	0	0	0	0	0
tr|F0JYI2|F0JYI	30 kDa	0	0	0	310	0	0	0	0	0
tr|R1CJD0|R1C	17 kDa	0	0	0	150	0	0	0	0	0
sp|B3WF44|RL35	8 kDa	0	0	190	0	0	0	0	0	0
tr|E4SW41|E4S	27 kDa	0	0	5	265	0	0	0	0	0
tr|G6EVI6|G6E	27 kDa	0	0	5	209	0	0	0	0	0
tr|K0NPK7|K0N	31 kDa	0	0	2	10	0	0	0	0	0
sp|B3WAL2|RL	13 kDa	0	5	224	0	0	0	0	1	0
tr|S2UB14|S2U	8 kDa	0	4	56	0	0	0	0	0	0
tr|S5DX82|S5D	45 kDa	0	0	0	0	109	41	68	7	0
tr|C9M1L3|C9N	48 kDa	0	0	0	0	93	31	70	7	0
tr|J4BP45|J4BP	45 kDa	0	0	0	0	41	22	39	5	0
tr|F3MNH8|F3	45 kDa	0	0	0	0	88	35	86	5	0
tr|A8YX17|A8Y	45 kDa	0	0	0	0	56	34	59	5	0
tr|G9ZRC2|G9ZR	38 kDa	0	0	0	0	0	390	394	51	0
tr|R5ZEC3|R5Z	54 kDa	0	174	0	0	0	0	0	0	0
tr|E4SIN8|E4SI	54 kDa	0	131	0	0	0	0	0	0	0
tr|E4SJL2|E4SJI	26 kDa	0	0	0	0	0	122	0	0	0
tr|C9M1B6|C9I	15 kDa	0	0	0	0	0	11	0	0	0
tr|A8YWF8|A8	57 kDa	0	38	0	0	19	20	41	2	0
tr|R5YS22|R5Y	57 kDa	0	47	0	0	12	20	38	2	0
tr|C2HQZ1|C2H	57 kDa	0	8	0	0	5	3	12	0	0
tr|E4SK72|E4SK7	41 kDa	0	136	0	0	0	0	0	0	0
tr|A8YWK1|A8YV	39 kDa	0	0	0	0	2	150	0	0	0
tr|G6F8P1|G6F8I	42 kDa	0	0	0	126	0	0	0	0	0
tr|F0JYR8|F0JY	33 kDa	0	0	0	141	0	0	1	0	0
sp|B3WE58|RL32	7 kDa	0	4	94	0	0	0	0	0	0
tr|C9LZ70|C9L	19 kDa	0	0	0	0	40	20	26	2	0
tr|F0NSG7|F0N	19 kDa	0	0	0	0	35	21	25	1	0
tr|F0K343|F0K34	66 kDa	0	0	0	133	0	0	0	0	0
tr|Q048B8|Q0	53 kDa	0	0	0	109	0	0	0	0	0
tr|D8FP44|D8F	74 kDa	0	0	0	78	0	0	0	0	0
tr|G6F744|G6F	74 kDa	0	0	0	98	0	0	0	0	0
tr|F0K061|F0K	74 kDa	0	0	0	97	0	0	0	0	0
tr|A4ZH10|A4Z	41 kDa	0	3	0	0	7	46	74	6	0
tr|A8YV73|A8Y	41 kDa	0	0	0	0	5	54	46	5	0
sp|A8YUI5|G6F	50 kDa	0	4	0	0	2	0	52	3	5
tr|C2KBW5|C2I	49 kDa	0	2	0	0	1	0	16	0	18
sp|B3WDC0|G	49 kDa	0	0	10	0	0	0	0	0	0
tr|N1ZU93|N1	49 kDa	0	0	0	0	0	0	13	0	8
tr|D5GZH9|D5	73 kDa	0	0	0	0	0	0	0	109	0
tr|C7XKH6|C7X	73 kDa	0	0	0	0	0	0	0	59	0
sp|B3WAK8|RS1	10 kDa	0	0	68	0	0	0	0	0	0
sp|Q04BA5|AT	55 kDa	0	3	0	37	8	0	11	0	0
tr|S5DVE6|S5D	55 kDa	0	0	0	16	20	0	23	0	0
sp|Q042L3|ATI	55 kDa	0	0	0	17	6	0	9	0	0
tr|D0DEM7|D0	55 kDa	0	3	0	22	4	0	12	0	0
tr|S5E0I5|S5E0	21 kDa	0	0	0	0	15	23	45	6	0
tr|A8YWD9|A8	21 kDa	0	0	0	0	14	23	44	6	0
tr|S5DZM9|S5DZ	28 kDa	0	9	0	0	52	7	34	0	0
sp|Q1G9Z4|BG	114 kDa	0	0	0	106	0	0	0	0	0
tr|F0K2P6|F0K	114 kDa	0	0	0	106	0	0	0	0	0
tr|S2PW45|S2I	48 kDa	0	0	99	0	0	0	0	0	0
tr|B5QKE3|B5C	48 kDa	0	0	57	0	0	0	0	0	0
tr|B3W6S8|B3	42 kDa	0	0	97	0	0	0	0	0	0
tr|S6C9A8|S6C	41 kDa	0	0	17	0	0	0	0	0	0
tr|F0K0F9|F0K	83 kDa	0	0	0	97	0	0	0	0	0
tr|F0JZK9|F0JZ	61 kDa	0	0	0	105	0	0	0	0	0
sp|Q1GBQ2|PY	61 kDa	0	0	0	94	0	0	0	0	0
tr|C9M1H5|C9	46 kDa	0	0	0	0	16	36	46	2	0
tr|A8YWP5|A8	46 kDa	0	0	0	0	16	40	40	2	0
tr|F0JZ25|F0JZ	34 kDa	0	0	0	71	0	0	0	0	0
tr|G6F802|G6F	58 kDa	0	0	0	70	0	0	0	0	0
tr|E4SVN4|E4S	56 kDa	0	0	0	48	0	0	0	0	0
tr|R1B2J2|R1B	58 kDa	0	0	0	38	0	0	0	0	0

<table>
<tr><td>sp|Q048U4|RL</td><td>18 kDa</td><td>0</td><td>1</td><td>4</td><td>66</td><td>0</td><td>0</td><td>0</td><td>0</td><td>0</td></tr>
<tr><td>tr|E4SWK5|E4S</td><td>18 kDa</td><td>0</td><td>1</td><td>4</td><td>51</td><td>0</td><td>0</td><td>0</td><td>0</td><td>0</td></tr>
<tr><td>tr|K0NNL1|K0N</td><td>18 kDa</td><td>0</td><td>1</td><td>1</td><td>17</td><td>0</td><td>0</td><td>0</td><td>0</td><td>0</td></tr>
<tr><td>tr|B3WEY9|B3WI</td><td>10 kDa</td><td>0</td><td>5</td><td>62</td><td>0</td><td>0</td><td>0</td><td>0</td><td>0</td><td>0</td></tr>
<tr><td>tr|F0NTU5|F0N</td><td>38 kDa</td><td>0</td><td>0</td><td>0</td><td>0</td><td>6</td><td>42</td><td>21</td><td>0</td><td>0</td></tr>
<tr><td>tr|F3MKP1|F3N</td><td>38 kDa</td><td>0</td><td>0</td><td>0</td><td>0</td><td>10</td><td>34</td><td>24</td><td>0</td><td>0</td></tr>
<tr><td>tr|C2KG71|C2KG</td><td>140 kDa</td><td>0</td><td>0</td><td>0</td><td>0</td><td>0</td><td>0</td><td>0</td><td>77</td><td>0</td></tr>
<tr><td>tr|D8FP85|D8F</td><td>69 kDa</td><td>0</td><td>0</td><td>0</td><td>76</td><td>0</td><td>0</td><td>0</td><td>0</td><td>0</td></tr>
<tr><td>tr|E4SYD1|E4S</td><td>70 kDa</td><td>0</td><td>0</td><td>0</td><td>69</td><td>0</td><td>0</td><td>0</td><td>0</td><td>0</td></tr>
<tr><td>sp|B3WE33|RS2C</td><td>9 kDa</td><td>0</td><td>5</td><td>48</td><td>0</td><td>0</td><td>0</td><td>0</td><td>0</td><td>0</td></tr>
<tr><td>tr|D8FRA2|D8F</td><td>92 kDa</td><td>0</td><td>0</td><td>0</td><td>36</td><td>3</td><td>0</td><td>0</td><td>0</td><td>0</td></tr>
<tr><td>sp|A8YUC4|SE(</td><td>92 kDa</td><td>0</td><td>0</td><td>0</td><td>10</td><td>3</td><td>0</td><td>1</td><td>0</td><td>0</td></tr>
<tr><td>tr|F0K210|F0K</td><td>90 kDa</td><td>0</td><td>2</td><td>0</td><td>18</td><td>3</td><td>0</td><td>0</td><td>0</td><td>0</td></tr>
<tr><td>tr|R1CME9|R1(</td><td>90 kDa</td><td>0</td><td>0</td><td>0</td><td>10</td><td>0</td><td>0</td><td>0</td><td>0</td><td>0</td></tr>
<tr><td>tr|E4SIR2|E4SIR2</td><td>44 kDa</td><td>0</td><td>62</td><td>0</td><td>0</td><td>0</td><td>0</td><td>0</td><td>0</td><td>0</td></tr>
<tr><td>tr|F0K3I8|F0K3I8</td><td>88 kDa</td><td>0</td><td>0</td><td>0</td><td>61</td><td>0</td><td>0</td><td>1</td><td>0</td><td>0</td></tr>
<tr><td>tr|F0K0F8|F0K(</td><td>58 kDa</td><td>0</td><td>0</td><td>0</td><td>59</td><td>0</td><td>0</td><td>0</td><td>0</td><td>0</td></tr>
<tr><td>sp|B3WAL5|RL</td><td>12 kDa</td><td>0</td><td>0</td><td>61</td><td>0</td><td>0</td><td>0</td><td>0</td><td>0</td><td>0</td></tr>
<tr><td>tr|K6RWM9|K6</td><td>12 kDa</td><td>0</td><td>0</td><td>48</td><td>0</td><td>0</td><td>0</td><td>0</td><td>0</td><td>0</td></tr>
<tr><td>tr|F0JZF2|F0JZI</td><td>47 kDa</td><td>0</td><td>0</td><td>0</td><td>55</td><td>0</td><td>0</td><td>0</td><td>0</td><td>0</td></tr>
<tr><td>tr|A4UAE7|A4U</td><td>35 kDa</td><td>0</td><td>0</td><td>1</td><td>0</td><td>0</td><td>0</td><td>26</td><td>1</td><td>4</td></tr>
<tr><td>sp|P00343|LDH</td><td>36 kDa</td><td>0</td><td>0</td><td>11</td><td>0</td><td>0</td><td>0</td><td>0</td><td>0</td><td>0</td></tr>
<tr><td>tr|F6CCW2|F6(</td><td>35 kDa</td><td>0</td><td>0</td><td>1</td><td>0</td><td>0</td><td>0</td><td>11</td><td>0</td><td>6</td></tr>
<tr><td>tr|E1NG16|E1N</td><td>35 kDa</td><td>0</td><td>0</td><td>0</td><td>66</td><td>6</td><td>0</td><td>0</td><td>0</td><td>0</td></tr>
<tr><td>tr|E7DZI3|E7DZ</td><td>35 kDa</td><td>0</td><td>66</td><td>0</td><td>0</td><td>0</td><td>0</td><td>0</td><td>1</td><td>1</td></tr>
<tr><td>sp|Q5FMB0|LD</td><td>35 kDa</td><td>0</td><td>0</td><td>0</td><td>69</td><td>0</td><td>0</td><td>0</td><td>0</td><td>0</td></tr>
<tr><td>tr|G2KWK8|G2</td><td>34 kDa</td><td>0</td><td>0</td><td>0</td><td>60</td><td>0</td><td>0</td><td>0</td><td>0</td><td>0</td></tr>
<tr><td>tr|M5J615|M5.</td><td>34 kDa</td><td>0</td><td>0</td><td>0</td><td>59</td><td>0</td><td>0</td><td>0</td><td>0</td><td>0</td></tr>
<tr><td>sp|Q049U9|SYP_</td><td>63 kDa</td><td>0</td><td>0</td><td>0</td><td>50</td><td>0</td><td>0</td><td>0</td><td>0</td><td>0</td></tr>
<tr><td>tr|E4SK59|E4SK5</td><td>45 kDa</td><td>0</td><td>0</td><td>0</td><td>49</td><td>0</td><td>0</td><td>0</td><td>0</td><td>0</td></tr>
<tr><td>sp|Q049U3|EFTS</td><td>37 kDa</td><td>0</td><td>0</td><td>0</td><td>43</td><td>0</td><td>0</td><td>0</td><td>0</td><td>0</td></tr>
<tr><td>tr|D8FN26|D8F</td><td>51 kDa</td><td>0</td><td>0</td><td>66</td><td>0</td><td>0</td><td>0</td><td>0</td><td>0</td><td>0</td></tr>
<tr><td>tr|G6EUG9|G6I</td><td>39 kDa</td><td>0</td><td>0</td><td>0</td><td>0</td><td>45</td><td>4</td><td>0</td><td>2</td><td>1</td></tr>
<tr><td>tr|E4SYX8|E4S</td><td>39 kDa</td><td>0</td><td>0</td><td>0</td><td>0</td><td>45</td><td>0</td><td>0</td><td>0</td><td>0</td></tr>
<tr><td>tr|G6F4J1|G6F4J</td><td>69 kDa</td><td>0</td><td>0</td><td>0</td><td>0</td><td>42</td><td>4</td><td>0</td><td>0</td><td>0</td></tr>
<tr><td>tr|C5F8A4|C5F8A</td><td>41 kDa</td><td>0</td><td>52</td><td>0</td><td>0</td><td>0</td><td>0</td><td>0</td><td>0</td><td>0</td></tr>
<tr><td>tr|F0K106|F0K10</td><td>51 kDa</td><td>0</td><td>44</td><td>0</td><td>0</td><td>0</td><td>0</td><td>0</td><td>0</td><td>0</td></tr>
<tr><td>tr|F0NUC1|F0N</td><td>41 kDa</td><td>0</td><td>0</td><td>0</td><td>51</td><td>0</td><td>0</td><td>0</td><td>0</td><td>0</td></tr>
<tr><td>tr|C9M1L4|C9N</td><td>41 kDa</td><td>0</td><td>0</td><td>0</td><td>50</td><td>0</td><td>0</td><td>0</td><td>0</td><td>0</td></tr>
<tr><td>tr|E4SN31|E4S</td><td>34 kDa</td><td>0</td><td>0</td><td>0</td><td>0</td><td>5</td><td>33</td><td>8</td><td>1</td><td>12</td></tr>
<tr><td>tr|C2EM17|C2I</td><td>34 kDa</td><td>0</td><td>0</td><td>54</td><td>0</td><td>0</td><td>0</td><td>0</td><td>0</td><td>0</td></tr>
<tr><td>tr|F0JZJ6|F0JZJ</td><td>99 kDa</td><td>0</td><td>0</td><td>47</td><td>0</td><td>0</td><td>0</td><td>0</td><td>0</td><td>0</td></tr>
<tr><td>tr|E4SW33|E4S</td><td>99 kDa</td><td>0</td><td>1</td><td>0</td><td>43</td><td>2</td><td>0</td><td>1</td><td>0</td><td>0</td></tr>
<tr><td>tr|S5DXX8|S5DX</td><td>199 kDa</td><td>0</td><td>0</td><td>0</td><td>43</td><td>0</td><td>0</td><td>1</td><td>0</td><td>0</td></tr>
<tr><td>tr|B3WE65|B3</td><td>63 kDa</td><td>0</td><td>0</td><td>0</td><td>50</td><td>0</td><td>0</td><td>0</td><td>0</td><td>0</td></tr>
<tr><td>tr|S2UFI9|S2UI</td><td>60 kDa</td><td>0</td><td>0</td><td>0</td><td>12</td><td>0</td><td>0</td><td>0</td><td>0</td><td>0</td></tr>
<tr><td>sp|Q047T2|ML</td><td>57 kDa</td><td>0</td><td>0</td><td>0</td><td>58</td><td>0</td><td>0</td><td>0</td><td>1</td><td>0</td></tr>
<tr><td>tr|E4SZ57|E4SZ</td><td>56 kDa</td><td>0</td><td>0</td><td>0</td><td>11</td><td>0</td><td>0</td><td>0</td><td>0</td><td>0</td></tr>
<tr><td>tr|I7LAM9|I7LA</td><td>56 kDa</td><td>0</td><td>0</td><td>0</td><td>52</td><td>0</td><td>0</td><td>0</td><td>0</td><td>0</td></tr>
<tr><td>tr|F0JZC5|F0JZ(</td><td>60 kDa</td><td>0</td><td>0</td><td>0</td><td>14</td><td>0</td><td>0</td><td>0</td><td>0</td><td>0</td></tr>
<tr><td>tr|F0HUM2|F0</td><td>60 kDa</td><td>0</td><td>0</td><td>0</td><td>5</td><td>0</td><td>0</td><td>35</td><td>0</td><td>0</td></tr>
<tr><td>tr|F9UN51|F9U</td><td>60 kDa</td><td>0</td><td>0</td><td>0</td><td>0</td><td>0</td><td>0</td><td>35</td><td>0</td><td>0</td></tr>
<tr><td>tr|G6F7E4|G6F</td><td>41 kDa</td><td>0</td><td>6</td><td>42</td><td>0</td><td>0</td><td>0</td><td>0</td><td>0</td><td>0</td></tr>
<tr><td>tr|K0NKB0|K0N</td><td>41 kDa</td><td>0</td><td>15</td><td>0</td><td>4</td><td>26</td><td>0</td><td>9</td><td>0</td><td>0</td></tr>
<tr><td>sp|Q59485|PEI</td><td>90 kDa</td><td>0</td><td>15</td><td>0</td><td>0</td><td>0</td><td>0</td><td>3</td><td>0</td><td>0</td></tr>
<tr><td>tr|C7XHB2|C7XHI</td><td>47 kDa</td><td>0</td><td>2</td><td>0</td><td>20</td><td>1</td><td>4</td><td>0</td><td>2</td><td>0</td></tr>
<tr><td>sp|B3WAJ1|RL17</td><td>14 kDa</td><td>0</td><td>0</td><td>0</td><td>20</td><td>0</td><td>0</td><td>0</td><td>0</td><td>0</td></tr>
<tr><td>sp|A8YTF2|RL7</td><td>12 kDa</td><td>0</td><td>0</td><td>0</td><td>41</td><td>0</td><td>0</td><td>0</td><td>0</td><td>0</td></tr>
<tr><td>tr|F0TIQ0|F0TI</td><td>12 kDa</td><td>0</td><td>0</td><td>0</td><td>0</td><td>0</td><td>27</td><td>0</td><td>0</td><td>0</td></tr>
<tr><td>tr|F0K0S4|F0K(</td><td>52 kDa</td><td>0</td><td>0</td><td>0</td><td>0</td><td>0</td><td>24</td><td>0</td><td>0</td><td>0</td></tr>
<tr><td>sp|Q1GAN1|SY</td><td>64 kDa</td><td>0</td><td>0</td><td>44</td><td>0</td><td>0</td><td>0</td><td>0</td><td>0</td><td>0</td></tr>
<tr><td>tr|A8YWJ6|A8Y</td><td>25 kDa</td><td>0</td><td>0</td><td>0</td><td>45</td><td>0</td><td>0</td><td>0</td><td>0</td><td>0</td></tr>
<tr><td>tr|F0THV2|F0T</td><td>25 kDa</td><td>0</td><td>0</td><td>0</td><td>46</td><td>0</td><td>0</td><td>0</td><td>0</td><td>0</td></tr>
<tr><td>sp|B3WAI0|RL</td><td>17 kDa</td><td>0</td><td>0</td><td>21</td><td>0</td><td>0</td><td>0</td><td>0</td><td>0</td><td>0</td></tr>
<tr><td>tr|K6SL09|K6SI</td><td>17 kDa</td><td>0</td><td>0</td><td>12</td><td>0</td><td>0</td><td>0</td><td>0</td><td>0</td><td>0</td></tr>
<tr><td>sp|Q1GAB5|PPA(</td><td>34 kDa</td><td>0</td><td>3</td><td>0</td><td>26</td><td>5</td><td>0</td><td>0</td><td>0</td><td>0</td></tr>
<tr><td>tr|Q04AM9|Q04</td><td>37 kDa</td><td>0</td><td>2</td><td>0</td><td>26</td><td>2</td><td>0</td><td>0</td><td>0</td><td>0</td></tr>
<tr><td>sp|B3WAH9|RS</td><td>14 kDa</td><td>0</td><td>0</td><td>32</td><td>0</td><td>0</td><td>0</td><td>0</td><td>0</td><td>0</td></tr>
<tr><td>tr|K6R5S2|K6R</td><td>14 kDa</td><td>0</td><td>0</td><td>38</td><td>0</td><td>0</td><td>0</td><td>0</td><td>0</td><td>0</td></tr>
<tr><td>tr|F0K0W9|F0K</td><td>35 kDa</td><td>0</td><td>0</td><td>0</td><td>0</td><td>0</td><td>0</td><td>38</td><td>1</td><td>0</td></tr>
<tr><td>tr|B3WD17|B3</td><td>66 kDa</td><td>0</td><td>0</td><td>0</td><td>41</td><td>0</td><td>0</td><td>0</td><td>0</td><td>0</td></tr>
<tr><td>tr|C2F9X0|C2F9X</td><td>50 kDa</td><td>0</td><td>0</td><td>0</td><td>41</td><td>0</td><td>0</td><td>0</td><td>0</td><td>0</td></tr>
<tr><td>tr|C9M4A3|C9I</td><td>33 kDa</td><td>0</td><td>0</td><td>0</td><td>40</td><td>0</td><td>0</td><td>0</td><td>0</td><td>0</td></tr>
<tr><td>tr|C2KD36|C2K</td><td>33 kDa</td><td>0</td><td>0</td><td>0</td><td>49</td><td>0</td><td>0</td><td>0</td><td>0</td><td>0</td></tr>
<tr><td>tr|F0K1H3|F0K</td><td>35 kDa</td><td>0</td><td>2</td><td>0</td><td>0</td><td>0</td><td>0</td><td>31</td><td>0</td><td>2</td></tr>
<tr><td>tr|E4SJT0|E4SJT0</td><td>46 kDa</td><td>0</td><td>2</td><td>0</td><td>3</td><td>0</td><td>0</td><td>12</td><td>0</td><td>6</td></tr>
<tr><td>tr|F0JYV9|F0JYV</td><td>37 kDa</td><td>0</td><td>0</td><td>38</td><td>0</td><td>0</td><td>0</td><td>0</td><td>0</td><td>0</td></tr>
<tr><td>tr|F0K0A2|F0K0A</td><td>96 kDa</td><td>0</td><td>0</td><td>38</td><td>0</td><td>0</td><td>0</td><td>0</td><td>0</td><td>0</td></tr>
<tr><td>tr|A8YU96|A8Y</td><td>64 kDa</td><td>0</td><td>0</td><td>28</td><td>0</td><td>0</td><td>0</td><td>0</td><td>0</td><td>0</td></tr>
<tr><td>tr|C2KBL1|C2K</td><td>63 kDa</td><td>0</td><td>0</td><td>28</td><td>0</td><td>0</td><td>0</td><td>0</td><td>0</td><td>0</td></tr>
<tr><td>tr|D8GEE0|D8(</td><td>34 kDa</td><td>0</td><td>0</td><td>0</td><td>22</td><td>0</td><td>0</td><td>0</td><td>0</td><td>0</td></tr>
<tr><td>tr|B3WBZ2|B3</td><td>34 kDa</td><td>0</td><td>0</td><td>0</td><td>22</td><td>0</td><td>0</td><td>0</td><td>0</td><td>0</td></tr>
<tr><td>sp|B3W7F8|RL</td><td>17 kDa</td><td>0</td><td>0</td><td>11</td><td>11</td><td>0</td><td>0</td><td>2</td><td>0</td><td>0</td></tr>
<tr><td>tr|S2N724|S2N</td><td>17 kDa</td><td>0</td><td>0</td><td>11</td><td>0</td><td>0</td><td>0</td><td>0</td><td>0</td><td>0</td></tr>
<tr><td>tr|F0K056|F0K(</td><td>88 kDa</td><td>0</td><td>0</td><td>0</td><td>25</td><td>0</td><td>0</td><td>0</td><td>0</td><td>0</td></tr>
<tr><td>tr|F0JZE7|F0JZE7</td><td>36 kDa</td><td>0</td><td>0</td><td>0</td><td>20</td><td>0</td><td>0</td><td>0</td><td>0</td><td>0</td></tr>
<tr><td>tr|B3WFA8|B3</td><td>77 kDa</td><td>0</td><td>0</td><td>0</td><td>40</td><td>0</td><td>0</td><td>0</td><td>0</td><td>0</td></tr>
<tr><td>tr|G6F770|G6F</td><td>41 kDa</td><td>0</td><td>0</td><td>0</td><td>0</td><td>0</td><td>0</td><td>0</td><td>39</td><td>0</td></tr>
<tr><td>tr|E4SXW7|E4S</td><td>40 kDa</td><td>0</td><td>0</td><td>0</td><td>0</td><td>0</td><td>0</td><td>0</td><td>39</td><td>0</td></tr>
<tr><td>tr|F0JZ23|F0JZ</td><td>43 kDa</td><td>0</td><td>0</td><td>0</td><td>35</td><td>0</td><td>0</td><td>0</td><td>0</td><td>0</td></tr>
<tr><td>tr|R1CXF1|R1C</td><td>43 kDa</td><td>0</td><td>0</td><td>33</td><td>0</td><td>0</td><td>0</td><td>0</td><td>0</td><td>0</td></tr>
<tr><td>tr|J7LNX2|J7LN</td><td>74 kDa</td><td>0</td><td>0</td><td>0</td><td>43</td><td>0</td><td>0</td><td>0</td><td>0</td><td>0</td></tr>
<tr><td>tr|F0K1P1|F0K</td><td>46 kDa</td><td>0</td><td>0</td><td>0</td><td>39</td><td>0</td><td>0</td><td>0</td><td>0</td><td>0</td></tr>
<tr><td>sp|Q03AK4|EN</td><td>47 kDa</td><td>0</td><td>0</td><td>24</td><td>0</td><td>0</td><td>0</td><td>0</td><td>1</td><td>0</td></tr>
<tr><td>tr|D8FMY1|D8FN</td><td>39 kDa</td><td>0</td><td>0</td><td>11</td><td>0</td><td>0</td><td>0</td><td>0</td><td>1</td><td>0</td></tr>
<tr><td>tr|F0K1Q5|F0K</td><td>49 kDa</td><td>0</td><td>0</td><td>0</td><td>23</td><td>0</td><td>0</td><td>0</td><td>0</td><td>0</td></tr>
</table>

sp|B3WAL3|RS	11 kDa	0	0	1	33	0	0	0	0	0
sp|Q38UR6|RS	11 kDa	0	0	0	32	0	0	0	0	0
tr|F0HXH1|F0H	47 kDa	0	0	0	32	0	0	0	0	0
sp|Q048F6|SYS	49 kDa	0	0	29	0	0	0	0	0	0
tr|D8FP51|D8F	43 kDa	0	0	26	0	0	0	0	0	0
tr|F0K0B3|F0K	52 kDa	0	0	0	0	1	10	21	0	0
sp|B3WAL7|RL	23 kDa	0	0	0	20	0	0	0	7	0
tr|C2JYI0|C2JYI	23 kDa	0	0	0	19	0	0	0	1	0
tr|C9M3R2|C9I	82 kDa	0	0	0	31	0	0	0	0	0
tr|A8YXN8|A8Y	82 kDa	0	0	0	19	0	0	0	0	0
tr|D8FPJ7|D8FI	31 kDa	0	0	21	0	0	0	0	0	0
sp|P54262|SYN	50 kDa	0	0	0	33	0	0	0	0	0
tr|K0NUN6|K0I	50 kDa	0	0	27	0	0	0	0	0	0
tr|B3WAW5|B3	57 kDa	0	0	0	34	0	0	0	0	0
tr|F0K3J1|F0K3	28 kDa	0	0	0	47	0	5	2	0	0
tr|C5F2U8|C5F2L	77 kDa	0	0	0	32	3	0	0	0	0
tr|F0JYK3|F0JYK3	33 kDa	0	0	0	0	1	0	0	19	81
tr|F0JZ55|F0JZ55	55 kDa	0	0	26	0	0	0	0	0	0
tr|F0JZR4|F0JZR4	39 kDa	0	0	17	0	0	0	0	0	0
tr|G6EWR9|G6EV	38 kDa	0	0	18	0	0	0	0	0	0
sp|Q048U5|RL7_	12 kDa	0	0	14	0	0	0	0	0	0
tr|F2M2I8|F2M2I	31 kDa	0	0	0	22	0	0	0	0	0
tr|K6Q4Z0|K6C	36 kDa	0	0	18	0	0	0	0	0	0
tr|B3WAU5|B3	36 kDa	0	0	16	0	0	0	0	0	0
tr|S2R7Y4|S2R	19 kDa	0	0	7	0	0	0	0	0	0
tr|F0K0B7|F0K	51 kDa	0	0	0	28	0	0	0	0	0
sp|B3W8W8|R	6 kDa	0	0	23	0	0	0	0	0	0
tr|K6SHA2|K6S	6 kDa	0	5	0	0	0	0	15	0	0
tr|F0JYY1|F0JY	37 kDa	0	0	4	24	0	0	0	0	0
tr|F0HVK2|F0H	33 kDa	0	0	2	24	0	0	0	0	0
sp|B3WAK0|RL3C	7 kDa	0	0	4	19	0	0	0	0	0
tr|A8YW42|A8	42 kDa	0	0	21	0	0	0	0	0	0
tr|A4ZH58|A4Z	43 kDa	0	0	0	23	0	0	0	0	0
tr|B3TNN9|B3T	43 kDa	0	0	0	0	19	0	0	0	0
tr|B3W6Z3|B3W	82 kDa	0	0	20	0	0	0	0	0	0
tr|B3W9U4|B3W	14 kDa	0	0	21	0	0	0	0	0	0
tr|F0JYZ4|F0JYZ4	113 kDa	0	2	24	0	0	0	0	0	0
tr|F0K1V4|F0K1V	36 kDa	0	25	0	0	0	0	6	10	1
tr|J7LF50|J7LF50	30 kDa	0	0	0	27	1	0	1	0	0
tr|S2LZU1|S2LZU	50 kDa	0	0	0	2	4	0	10	0	1
tr|B3WEB6|B3WI	37 kDa	0	0	0	0	4	0	10	0	0
sp|B3WAL0|RL16	16 kDa	0	0	19	0	0	0	0	0	0
tr|E4SIL6|E4SIL6_	42 kDa	0	0	15	0	0	0	0	0	0
tr|F0JZK2|F0JZK2	31 kDa	0	20	0	0	0	0	0	0	0
tr|A8YTM6|A8	57 kDa	0	0	0	12	0	0	0	0	0
tr|B3WAW4|B3W	83 kDa	0	0	0	18	0	0	0	0	0
tr|B3WEZ1|B3WI	11 kDa	0	0	0	23	0	0	0	0	0
tr|E4SJU1|E4SJU	19 kDa	0	0	0	23	0	0	0	0	0
tr|D8FPV8|D8F	57 kDa	0	0	11	0	0	0	0	0	0
tr|F0K1C9|F0K	56 kDa	0	0	11	0	0	0	0	0	0
tr|G6F842|G6F	49 kDa	0	0	13	0	0	0	0	0	0
tr|F0JYY5|F0JY	48 kDa	0	0	13	0	0	0	0	0	0
tr|C2FDY3|C2F	100 kDa	0	0	0	21	0	0	0	0	0
sp|B3WE34|RS15	10 kDa	0	0	0	21	0	0	0	0	0
tr|B3WEB4|B3	85 kDa	0	0	0	22	0	0	0	0	0
tr|B5QQY5|B5	85 kDa	0	0	0	27	0	0	1	0	1
tr|F0K0S2|F0K	34 kDa	0	0	0	21	0	0	0	0	0
tr|D8FNV3|D8FN	39 kDa	0	0	0	21	0	0	0	0	0
tr|F0JYT8|F0JYT8	45 kDa	0	0	0	21	0	0	0	0	0
tr|F0K0T6|F0K0T	63 kDa	0	0	0	24	0	0	0	0	0
tr|F0NWR2|F0NV	50 kDa	0	0	0	24	0	0	0	0	0
tr|F0JYP1|F0JY	39 kDa	0	0	0	0	0	0	15	0	0
tr|G6EUG1|G6EU	39 kDa	0	0	0	0	0	0	11	0	0
tr|D8FQ53|D8	70 kDa	0	0	0	20	0	0	0	0	0
tr|A8YX34|A8Y	50 kDa	0	0	0	17	0	0	0	0	0
tr|C2HLZ7|C2H	51 kDa	0	0	0	20	0	0	0	0	0
tr|F0JZI4|F0JZI	30 kDa	0	0	0	10	0	0	0	0	0
sp|Q04CC7|SY	48 kDa	0	0	0	0	1	18	0	0	0
tr|G6F413|G6F	33 kDa	0	0	0	18	0	0	0	0	0
tr|G6ESC1|G6E	22 kDa	0	0	0	14	0	0	0	0	0
sp|B3WE64|K6PF	34 kDa	0	0	2	21	0	0	0	0	0
tr|A8YXH6|A8YX	39 kDa	0	0	0	0	0	0	0	0	22
tr|D8FQD7|D8FC	53 kDa	0	1	0	10	1	0	2	0	0
tr|E4SXX2|E4SX	36 kDa	0	1	0	10	1	0	0	0	0
tr|F0HV09|F0HV	13 kDa	0	0	0	0	2	0	12	0	0
tr|F0K0G2|F0K0C	23 kDa	0	0	0	0	2	0	12	0	0
tr|F6CCA9|F6CC	27 kDa	0	0	13	0	0	0	0	0	0
sp|Q04AY6|DE	49 kDa	0	0	7	0	0	0	0	0	0
tr|A8YUD2|A8	64 kDa	0	0	0	16	2	21	4	0	0
tr|B3WD80|B3	23 kDa	0	0	16	0	0	0	0	0	0
tr|B5QN95|B5	23 kDa	0	0	16	0	0	0	0	0	0
sp|Q1GBI6|RL13_	16 kDa	0	0	4	0	0	0	0	0	0
tr|F6CCE1|F6CCE	38 kDa	0	0	19	0	0	0	0	0	0
tr|S5DWF2|S5DV	53 kDa	0	0	16	0	0	0	0	0	0
tr|C5F593|C5F	47 kDa	0	0	0	0	7	10	2	0	0
tr|S6CAD9|S6C	47 kDa	0	0	0	5	16	0	1	1	0
sp|Q034Z1|RL29_	7 kDa	0	0	0	5	16	0	1	1	0
tr|B3W8T5|B3W	27 kDa	0	0	0	17	0	0	0	0	0
tr|B3WA57|B3W	21 kDa	0	0	0	17	0	0	0	0	0
tr|C9LZX4|C9LZX	18 kDa	0	0	0	11	0	0	0	0	0
tr|A4ZGX2|A4Z	50 kDa	0	0	0	18	0	0	1	0	0
tr|F0K1B0|F0K	43 kDa	0	0	0	13	0	0	0	0	0
tr|D8FRG6|D8	43 kDa	0	0	0	20	0	0	0	0	0
tr|D8FLW5|D8	61 kDa	0	0	0	10	0	0	0	0	0
sp|Q1GBJ7|KAI	24 kDa	0	0	0	14	0	0	0	0	0
tr|E4SVS1|E4S	27 kDa	0	0	0	14	1	0	0	0	0

tr|A8YWC4|A8Y	27 kDa	0	0	0	17	0	0	1	0	0
sp|B3WAK2|RL18	13 kDa	0	0	0	15	0	0	1	0	0
tr|G6F7Y5|G6F7Y	76 kDa	0	0	0	17	0	0	1	0	0
sp|Q1GB17|PO	41 kDa	0	0	0	18	0	0	0	0	0
sp|Q04AV4|SYI	49 kDa	0	0	0	17	0	0	0	0	0
sp|Q1GAG8|SY	49 kDa	0	0	0	16	0	0	0	0	0
tr|F0K0B1|F0K(	41 kDa	0	0	0	16	0	0	0	0	0
sp|B3WCW6|TPIS	27 kDa	0	1	0	13	0	0	0	0	0
sp|P22733|LACY_	68 kDa	0	1	0	13	0	0	0	0	0
tr|F0K010|F0K01	34 kDa	0	0	0	11	0	0	0	0	0
tr|F0K0G5|F0K0G	32 kDa	0	0	0	11	0	0	0	0	0
tr|F0K384|F0K38	41 kDa	0	0	0	17	0	0	0	0	0
tr|F0K095|F0K(	27 kDa	0	0	0	14	0	0	0	0	0
sp|Q1G9C7|SYI	40 kDa	0	0	0	0	10	0	0	0	0
sp|Q049X1|APT_	19 kDa	0	0	0	14	0	0	0	0	0
tr|F0K2X2|F0K2X	34 kDa	0	0	0	14	0	0	0	0	0
tr|G6F5A7|G6F5/	41 kDa	0	0	0	14	0	0	0	0	0
tr|K6S8B1|K6S8B	14 kDa	0	0	0	10	0	0	0	0	0
tr|S5DWF5|S5DV	40 kDa	0	0	1	17	0	0	0	0	0
tr|F0K105|F0K10	51 kDa	0	0	0	17	0	0	0	0	0
tr|E4T001|E4T(	46 kDa	0	0	0	14	0	0	0	0	0
tr|S2KR84|S2KI	46 kDa	0	0	14	0	0	0	0	0	0
tr|F0JZK5|F0JZI	35 kDa	0	15	0	0	0	0	0	0	0
sp|P80019|K6PF_	34 kDa	0	0	0	0	0	0	11	0	1
sp|Q04C00|RL6_I	19 kDa	0	0	0	0	0	0	11	0	0
tr|B3W8H8|B3W	33 kDa	0	0	0	10	1	0	0	0	0
tr|G6ESP1|G6ESF	45 kDa	0	0	0	10	1	0	0	0	0
tr|F2M3J6|F2M3.	43 kDa	0	0	0	10	0	0	0	0	0
tr|C9M278|C9I	51 kDa	0	0	0	16	0	0	0	0	0
tr|K0NSC4|K0N	49 kDa	0	0	0	15	0	0	0	0	0
tr|F0JZT2|F0JZ'	46 kDa	0	0	0	24	0	0	0	0	0
tr|F0K011|F0K(	28 kDa	0	3	0	17	0	0	0	0	0
tr|G6EV17|G6E	28 kDa	0	14	0	0	0	0	0	0	0
tr|F0K0K4|F0K(	28 kDa	0	0	0	12	0	0	0	0	0
tr|D8FLF1|D8F	28 kDa	0	0	12	0	0	0	0	0	0
tr|E4SWL8|E4SW	27 kDa	0	0	12	0	0	0	0	0	0
tr|R5ZFC8|R5ZFC	16 kDa	0	0	1	20	0	0	0	0	0
tr|F0K387|F0K:	73 kDa	0	0	13	0	0	0	0	0	0
tr|B3WEH2|B3'	82 kDa	0	0	0	12	0	0	0	0	0
tr|E4SYE1|E4SYE:	40 kDa	0	0	0	15	0	0	0	0	0
tr|F0K2W1|F0K2'	32 kDa	0	0	0	15	0	0	0	0	0
tr|S2MNT7|S2MI	36 kDa	0	0	0	13	0	0	0	0	0
tr|D8FLP4|D8F	48 kDa	0	0	0	15	0	0	0	0	0
tr|F0JZE8|F0JZI	50 kDa	0	0	10	0	0	0	0	0	0
tr|E4SVY9|E4S'	50 kDa	0	0	0	14	0	0	0	0	0
sp|B3WA08|RL1_	24 kDa	0	11	0	0	0	0	0	0	0
sp|Q04A20|ERA_	34 kDa	0	0	0	11	0	0	0	0	0
sp|Q038L9|SYP_l	64 kDa	0	0	0	12	0	0	0	0	0
tr|B3WCK8|B3W(	10 kDa	0	0	0	12	0	0	0	0	0
tr|D8FPL3|D8FPL	84 kDa	0	0	0	10	0	0	0	0	0
tr|E4SLE9|E4SLE!	40 kDa	0	0	0	11	0	0	0	0	0
tr|F0JZT8|F0JZT8	26 kDa	0	0	0	12	0	0	0	0	0
tr|Q1G8F1|Q1(	43 kDa	0	0	0	0	10	0	0	0	0
tr|E4SWM9|E4SV	26 kDa	0	0	0	11	0	0	0	0	0
tr|F0HUQ5|F0HU	17 kDa	0	0	0	11	0	0	0	0	0
tr|F0K1Q2|F0K1C	32 kDa	0	0	0	10	0	0	0	0	0
tr|F0K355|F0K35	133 kDa	0	0	0	11	0	0	0	0	0
tr|D4YSH9|D4YSI	18 kDa	0	2	0	18	0	0	0	0	0
tr|D8FRD0|D8F	36 kDa	0	0	1	13	0	0	0	0	0
tr|D8FRH5|D8FRI	85 kDa	0	0	1	13	0	0	0	0	0
tr|Q04B82|Q04B:	30 kDa	0	0	1	13	0	0	0	0	0
sp|Q049G2|RL35	8 kDa	0	0	0	11	0	0	0	0	0
tr|A8YXF4|A8YXF	13 kDa	0	0	0	7	0	0	0	0	0
tr|C0WLS5|C0\	36 kDa	0	0	0	7	0	0	0	0	0
tr|C2CY45|C2C	36 kDa	0	0	0	7	0	0	0	0	0
tr|D8FPH3|D8FPI	11 kDa	0	0	0	7	0	0	0	0	0
tr|D8FPV5|D8FP\	49 kDa	0	0	0	12	0	0	0	0	0
tr|E4SYW8|E4SY\	66 kDa	0	0	0	10	1	0	2	0	0
tr|E4SZ83|E4SZ8:	54 kDa	0	0	0	11	0	0	2	0	0
tr|F0JYI4|F0JYI4_	21 kDa	0	0	0	11	0	0	2	0	0
tr|F0JZP6|F0JZP6	38 kDa	0	19	0	0	0	0	1	0	0

Supplemental Table 2: Putative SLAPs in *L. acidophilus* , *L. helveticus* , *L. amylovorus* , and *L. crispatus*

Identified protein	ORF	UniProt ID	Organism	Molecular weight	Pfam domain(s)	Amino Acid coverage	Spectral count
			***L. acidophilus* NCFM**				
Putative serine protease	LBA1578	Q5FIS9	*L. acidophilus* NCFM	78 kDa	-	583/694 (84%)	1129
Putative uncharacterized protein	LBA0695	Q5FL54	*L. acidophilus* NCFM	62 kDa	Big_3;SH3_8	410/550 (75%)	898
Putative uncharacterized protein	LBA0222	Q5FMF7	*L. acidophilus* NCFM	30 kDa	-	152/282 (54%)	609
Putative surface layer protein	LBA1029	Q5FK97	*L. acidophilus* NCFM	43 kDa	-	300/385 (78%)	562
SlpX	LBA0512	Q5FLN0	*L. acidophilus* NCFM	54 kDa	SLAP	324/499 (65%)	428
Cell separation protein	cdpA (LBA0223)	Q5FMF6	*L. acidophilus* NCFM	64 kDa	SLAP (2)	372/599 (62%)	276
Putative uncharacterized protein	LBA1426	Q5FJ73	*L. acidophilus* NCFM	28 kDa	-	159/252 (69%)	255
Aminopeptidase	LBA1567	Q5FIU0	*L. acidophilus* NCFM	57 kDa	Peptidase_M1	339/505 (67%)	265
Putative uncharacterized protein	LBA0864	Q5FKQ1	*L. acidophilus* NCFM	55 kDa	-	316/467 (64%)	255
Putative fibronectin domain	LBA0191	Q5FMI7	*L. acidophilus* NCFM	52 kDa	fn3	329/463 (71%)	249
Putative surface protein	LBA1568	Q5FIT9	*L. acidophilus* NCFM	39 kDa	-	233/353 (66%)	226
Putative uncharacterized protein	LBA1539	Q5FIW6	*L. acidophilus* NCFM	19 kDa	-	122/171 (71%)	196
Penecillin-binding protein	LBA0858	Q5FKQ5	*L. acidophilus* NCFM	42 kDa	Beta-lactamase	248/369 (67%)	171
Oligopeptide ABC transporter substrate bindin	oppA (LBA0197)	Q5FMI2	*L. acidophilus* NCFM	65 kDa	SBP_bac_5	366/585 (63%)	169
Lysin	lysA (LBA0851)	Q5FKR2	*L. acidophilus* NCFM	35 kDa	N/A	226/323 (70%)	157
Penecillin-binding protein	LBA0805	Q5FKV5	*L. acidophilus* NCFM	79 kDa	PASTA	379/720 (53%)	156
Putative uncharacterized protein	LBA1227	Q5FJR3	*L. acidophilus* NCFM	21 kDa	-	109/182 (60%)	128
Penecillin-binding protein	LBA1006	Q5FKB8	*L. acidophilus* NCFM	41 kDa	Beta-lactamase	276/364 (76%)	127
Putative alkylphosphonate ABC transporter pro	LBA0014	Q5FN03	*L. acidophilus* NCFM	35 kDa	-	187/313 (60%)	124
Glycerol-3-phosphate ABC transporter	LBA1641	Q5FIM1	*L. acidophilus* NCFM	47 kDa	-	293/433 (68%)	118
Putative membrane protein	LBA1690	Q5FIH2	*L. acidophilus* NCFM	31 kDa	-	207/280 (74%)	116
Foldase	prsA (LBA1588)	Q5FIS0	*L. acidophilus* NCFM	33 kDa	Foldase	193/300 (64%)	116
Putative enterolysin A	LBA1207	Q5FJT1	*L. acidophilus* NCFM	24 kDa	Peptidase_M23	139/213 (65%)	89
Putative membrane protein	LBA1661	Q5FIK1	*L. acidophilus* NCFM	20 kDa	PepSY (2)	86/180 (48%)	77
Putative uncharacterized protein	LBA0040	Q5FMX9	*L. acidophilus* NCFM	10 kDa	DUF4430	64/87 (74%)	74
N-acetylmuramidase	LBA0176	Q5FMJ9	*L. acidophilus* NCFM	45 kDa	Glucosaminidase; SLAP	176/409 (43%)	61
Putative surface exclusion protein	LBA0494	Q5FLP5	*L. acidophilus* NCFM	40 kDa	-	168/355 (47%)	50
Autolysin, amidase	LBA0177	Q5FMJ8	*L. acidophilus* NCFM	41 kDa	Amidase_2; SLAP	160/364 (44%)	42
Putative uncharacterized protein	LBA0046	Q5FMX4	*L. acidophilus* NCFM	13 kDa	-	62/118 (53%)	40
Putative cell surface protein	LBA1079	Q5FK50	*L. acidophilus* NCFM	23 kDa	ykuD	79/202 (39%)	34
			***L. helveticus* CNRZ32**				
Putative bacterial surface layer protein	lhe_1849	S5E4S6	*L. helveticus* CNRZ32	37 kDa	SLAP	214/338 (63%)	841
Surface layer protein	lhe_0185	S5DTT0	*L. helveticus* CNRZ32	47 kDa	SLAP	352/437 (81%)	767
Uncharacterized protein	lhe_0702	S5DUP5	*L. helveticus* CNRZ32	61 kDa	Big_3; SH3_8	357/541 (66%)	629
Uncharacterized protein	lhe_1516	S5E402	*L. helveticus* CNRZ32	35 kDa	-	190/325 (58%)	443
Uncharacterized protein	lhe_0573	S5DUF9	*L. helveticus* CNRZ32	31 kDa	-	140/247 (51%)	427
Putative bacterial surface layer protein	lhe_1848	S5DY29	*L. helveticus* CNRZ32	49 kDa	SLAP	239/464 (52%)	288
Uncharacterized protein	lhe_1520	S5E8T5	*L. helveticus* CNRZ32	24 kDa	-	96/214 (45%)	206
30S ribosomal protein S2	rpsB (lhe_1284)	S5DWQ6	*L. helveticus* CNRZ32	29 kDa	N/A	190/257 (74%)	181
Glutamate tRNA ligase	gltX (lhe_1731)	S5DXP8	*L. helveticus* CNRZ32	58 kDa	N/A	199/499 (40%)	177
Uncharacterized protein	lhe_0056	S5DTJ2	*L. helveticus* CNRZ32	15 kDa	DUF4430	57/136 (42%)	145
Oligopeptide ABC transport protein periplasmi	oppA2 (lhe_1309)	S5E085	*L. helveticus* CNRZ32	65 kDa	SBP_bac_5	192/583 (33%)	127
Fibronectin domain-containing protein	lhe_1881	S5E4V9	*L. helveticus* CNRZ32	52 kDa	fn3	191/464 (41%)	125
N-acetylmuramidase	lhe_0189	S5DX82	*L. helveticus* CNRZ32	45 kDa	Glucosaminidase; SLAP	165/407 (41%)	112
Proline tRNA ligase	proS (lhe_1277)	S5DWW8	*L. helveticus* CNRZ32	63 kDa	N/A	210/565 (37%)	109
30S ribosomal protein S1	lhe_0967	S5DZ59	*L. helveticus* CNRZ32	44 kDa	N/A	149/403 (37%)	85
DNA gyrase subunit A	gyrA (lhe_0006)	S5DWM8	*L. helveticus* CNRZ32	92 kDa	N/A	244/827 (30%)	81
30S ribosomal protein S7	rpsG (lhe_1792)	S5E0Z5	*L. helveticus* CNRZ32	18 kDa	N/A	80/156 (51%)	69
Pyruvate kinase	lhe_0957	S5DZ51	*L. helveticus* CNRZ32	63 kDa	N/A	198/589 (34%)	67
Conserved hypothetical penicillin-binding prot	pbpX (lhe_0876)	A4ZH27	*L. helveticus* CNRZ32	41 kDa	beta-lactamase	106/360 (29%)	62
Putative lactocepin S-layer protein	lhe_1255	S5DWI6	*L. helveticus* CNRZ32	19 kDa	SLAP (2)	66/165 (40%)	61
Uncharacterized protein	lhe_0100	S5DZM9	*L. helveticus* CNRZ32	28 kDa	-	77/245 (31%)	55
GroEL chaperonin	GroL (lhe_1673)	A4ZGY6	*L. helveticus* CNRZ32	58 kDa	N/A	220/540 (41%)	53
Autolysin, amidase	lhe_0190	S5DTT4	*L. helveticus* CNRZ32	41 kDa	Amidase (2); SLAP	112/363 (31%)	50
50S ribosomal protein L18	rplL (lhe_1711)	S5E0T9	*L. helveticus* CNRZ32	13 kDa	N/A	43/119 (36%)	47
Phenylalanine tRNA ligase beta subunit	pheT (lhe_1463)	S5E3W0	*L. helveticus* CNRZ32	89 kDa	N/A	154/804 (19%)	44
Threonine tRNA ligase	thrS (lhe_1489)	S5E3X9	*L. helveticus* CNRZ32	74 kDa	N/A	135/644 (21%)	43
Serine hydroxymethyltransferase	glyA (lhe_1825)	C4MCM0	*L. helveticus* CNRZ32	45 kDa	N/A	89/411 (22%)	41
Translation initiation factor IF-2	infB (lhe_1271)	S5DZN8	*L. helveticus* CNRZ32	97 kDa	N/A	132/820 (15%)	38
50S ribosomal protein L20	rplT (lhe_1479)	S5DX46	*L. helveticus* CNRZ32	14 kDa	N/A	50/118 (42%)	38
50S ribosomal protein L7/L12	rplL (lhe_1711)	S5E0T9	*L. helveticus* CNRZ32	12 kDa	N/A	74/120 (62%)	33
Glycerol-3-phosphate ABC transport protein pe	lhe_0473	S5DXX4	*L. helveticus* CNRZ32	48 kDa	-	128/433 (30%)	33
Purine operon repressor	lhe_1853	S5DY32	*L. helveticus* CNRZ32	31 kDa	N/A	94/276 (34%)	33
Asparagine tRNA ligase	asnS (lhe_1150)	S5E325	*L. helveticus* CNRZ32	50 kDa	N/A	97/432 (22%)	32
Enolase	eno (lhe_0896)	S5E776	*L. helveticus* CNRZ32	47 kDa	N/A	117/428 (27%)	31
Elogation factor Tu	tuf (lhe_0862)	S5E2B3	*L. helveticus* CNRZ32	44 kDa	N/A	108/396 (27%)	31
DNA-directed RNA polymerase subunit beta	rpoB (lhe_1796)	S5E9C3	*L. helveticus* CNRZ32	136 kDa	N/A	104/1213 (9%)	26
ABC transport protein substrate-binding comp	malE (lhe_0253)	S5DXE6	*L. helveticus* CNRZ32	44 kDa	-	147/408 (36%)	25
ATP synthase subunit alpha	atpA (lhe_0780)	S5DVE6	*L. helveticus* CNRZ32	55 kDa	N/A	79/503 (16%)	25
Membrane alanine aminopeptidase	pepM1 (lhe_1515)	G8DA67	*L. helveticus* CNRZ32	57 kDa	N/A	96/504 (19%)	23
Probable tRNA sulfurtransferase	thiI (lhe_0795)	S5DVF8	*L. helveticus* CNRZ32	46 kDa	N/A	108/405 (17%)	23
Uncharacterized protein	lhe_0099	S5DTK6	*L. helveticus* CNRZ32	41 kDa	SLAP	107/373 (29%)	22
Foldase protein PrsA	prsA (lhe_1533)	A4UAE0	*L. helveticus* CNRZ32	33 kDa	N/A	70/300 (23%)	22
DNA topoisomerase 1	topA (lhe_0980)	S5DVV3	*L. helveticus* CNRZ32	81 kDa	N/A	85/704 (12%)	21
Uncharacterized protein	lhe_401	S5E0Y4	*L. helveticus* CNRZ32	38 kDa	CAP	59/337 (18%)	20
30S ribosomal protein S17	rpsQ (lhe_1780)	S5DXT7	*L. helveticus* CNRZ32	11 kDa	N/A	31/88 (35%)	20
			***L. helveticus* 481-C**				
Putative bacterial surface layer protein	lhe_1849	S5E4S6	*L. helveticus* CNRZ32	37 kDa	SLAP	195/338 (58%)	776
Putative bacterial surface layer protein	lhe_1848	S5DY29	*L. helveticus* CNRZ32	49 kDa	SLAP	184/464 (40%)	619
Uncharacterized protein	lhe_0702	S5DUP5	*L. helveticus* CNRZ32	61 kDa	Big_3; SH3_8	414/541 (77%)	392

Fibronectin domain-containing protein	lhe_1881	S5E4V9	*L. helveticus* CNRZ32	52 kDa	fn3	336/464 (72%)	261
Uncharacterized protein	lhe_0099	S5DTK6	*L. helveticus CNRZ32*	41 kDa	SLAP	213/373 (57%)	197
Oligopeptide ABC transport protein periplasmi	oppA2 (lhe_1309)	S5E085	*L. helveticus* CNRZ32	65 kDa	SBP_bac_5	310/583 (53%)	162
Surface layer protein	lhe_0185	S5DTT0	*L. helveticus* CNRZ32	47 kDa	SLAP	58/437 (13%)	136
Lysin	lhe_0210	S5E5K2	*L. helveticus* CNRZ32	29 kDa	Glyco_hydro_25	154/276 (56%)	81
Uncharacterized protein	lhe_1516	S5E402	*L. helveticus* CNRZ32	35 kDa	-	118/325 (36%)	71
Conserved hypothetical penecillin-binding prot	pbpX (lhe_0876)	A4ZH27	*L. helveticus* CNRZ32	41 kDa	beta-lactamase	175/360 (49%)	69
Uncharacterized protein	lhe_0056	S5DTJ2	*L. helveticus* CNRZ32	15 kDa	DUF4430	53/136 (39%)	54
Conserved hypothetical penecillin-binding prot	pbpC1 (lhe_1002)	A4ZH10	*L. helveticus* CNRZ32	41 kDa	beta-lactamase	164/365 (45%)	48
N-acetylmuramidase	lhe_0189	S5DX82	*L. helveticus* CNRZ32	45 kDa	glucosamidase; SLAP	117/407 (29%)	40
Uncharacterized protein	lhe_0152	S5E573	*L. helveticus* CNRZ32	46 kDa	CAP	158/411 (38%)	35
Lactocepin H proteinase PrtH	lhe_1828	S5DXX8	*L. helveticus* CNRZ32	199 kDa	DUF1034; Inhibitor_I9; PA; Pepti	328/1843 (18%)	34
Foldase protein PrsA	prsA (lhe_1533)	A4UAE0	*L. helveticus* CNRZ32	33 kDa	N/A	171/300 (57%)	34
Uncharacterized protein	lhe_1445	S5E0G0	*L. helveticus* CNRZ32	36 kDa	-	127/333 (38%)	33
Conserved hypothetical penecillin-binding prot	pbpC2 (lhe_0078)	A4ZGY7	*L. helveticus* CNRZ32	38 kDa	beta-lactamase	184/336 (55%)	31
Membrane alanin aminopeptidase	pepM1 (lhe_1515)	G8DA67	*L. helveticus* CNRZ32	57 kDa	N/A	167/504 (33%)	22
Uncharacterized protein	lhe_1487	S5E0I5	*L. helveticus* CNRZ32	21 kDa	-	65/186 (35%)	22
Glycerol-3-phosphate ABC transport protein pe	lhe_0473	S5DXX4	*L. helveticus* CNRZ32	48 kDa	-	128/433 (30%)	20
			***L. helveticus* 15009**				
Putative bacterial surface layer protein	lhe_1849	S5E4S6	*L. helveticus* CNRZ32	37 kDa	SLAP	201/338 (59%)	1336
Uncharacterized protein	lhe_0702	S5DUP5	*L. helveticus* CNRZ32	61 kDa	Big_3; SH3_8	432/541 (80%)	615
Putative bacterial surface layer protein	lhe_1848	S5DY29	*L. helveticus* CNRZ32	49 kDa	SLAP	266/464 (57%)	597
Uncharacterized protein	lhe_573	S5DUF9	*L. helveticus* CNRZ32	31 kDa	-	142/274 (52%)	410
Fibronectin domain-containing protein	lhe_1881	S5E4V9	*L. helveticus* CNRZ32	52 kDa	fn3	335/464 (72%)	297
Oligopeptide ABC transport protein periplasmi	oppA2 (lhe_1309)	S5E085	*L. helveticus* CNRZ32	65 kDa	SBP_bac_5	368/583 (63%)	292
ATP-dependent protease ATP-binding subunit	clpE (lhe_0217)	S5DXB1	*L. helveticus* CNRZ32	79 kDa	N/A	519/707 (73%)	243
Uncharacterized protein	lhe_1516	S5E402	*L. helveticus* CNRZ32	35 kDa	-	183/325 (56%)	184
GroEL chaperonin	GroL (lhe_1673)	A4ZGY6	*L. helveticus* CNRZ32	58 kDa	N/A	395/540 (73%)	166
Pyruvate kinase	lhe_0957	S5DZ51	*L. helveticus* CNRZ32	63 kDa	N/A	410/589 (70%)	161
Lactocepin H3 proteinase	prtH3 (lhe_1520)	G8DA68	*L. helveticus* CNRZ32	179 kDa	DUF1034; Peptidase_s8	697/1637 (43%)	139
Enolase	eno (lhe_0896)	S5E776	*L. helveticus* CNRZ32	47 kDa	N/A	332/428 (78%)	132
Uncharacterized protein	lhe_0056	S5DTJ2	*L. helveticus* CNRZ32	15 kDa	DUF4430	67/136 (49%)	108
30S ribosomal protein S1	lhe_0967	S5DZ59	*L. helveticus* CNRZ32	44 kDa	N/A	272/403 (67%)	99
Surface layer protein	lhe_0185	S5DTT0	*L. helveticus* CNRZ32	47 kDa	SLAP	106/437 (24%)	92
Elogation factor Tu	tuf (lhe_0862)	S5E2B3	*L. helveticus* CNRZ32	44 kDa	N/A	217/396 (55%)	92
			***L. amylovorus* ATCC 3620**				
Uncharacterized protein	LA2_01250	E4SKI1	*L. amylovorus* GRL 111	28 kDa	-	133/256 (52%)	791
Uncharacterized protein	LA2_00480	E4SJM6	*L. amylovorus* GRL 111	34 kDa	SLAP	181/303 (60%)	514
Uncharacterized protein	LA2_03575	E4SN46	*L. amylovorus* GRL 111	61 kDa	Big_3	275/542 (51%)	501
Cell Separation protein	LA2_01255	E4SKI2	*L. amylovorus* GRL 111	62 kDa	FIVAR; SLAP (3)	236/582 (41%)	444
Uncharacterized protein	LA2_02085	E4SLF0	*L. amylovorus* GRL 111	28 kDa	SLAP	164/240 (68%)	433
S-layer protein	LA2_08875	E4SLY7	*L. amylovorus* GRL 111	71 kDa	Ig/albumin binding	260/683 (41%)	348
Uncharacterized protein	LA2_05380	E4SIN8	*L. amylovorus* GRL 111	54 kDa	Fn3 (2)	146/479 (30%)	173
30S ribosomal protein S2	rpsB (LA2_0717	E4SK06	*L. amylovorus* GRL 111	29 kDa	N/A	137/257 (53%)	171
Fibronectin-domain protein	LA2_01095	E4SK72	*L. amylovorus* GRL 111	41 kDa	-	185/364 (51%)	157
Lysin	LA2_07550	E4SKF6	*L. amylovorus* GRL 111	44 kDa	Glyco_hydro_25; SLAP	93/409 (23%)	139
30S ribosomal protein S1	rpsA (LA2_0504	E4SIH2	*L. amylovorus* GRL 111	44 kDa	N/A	201/403 (50%)	125
SlpX	LA2_02740	E4SM72	*L. amylovorus* GRL 111	53 kDa	SLAP	45/501 (9%)	113
Glutamate tRNA ligase	gltX (LA2_0179!	E4SL16	*L. amylovorus* GRL 111	58 kDa	N/A	189/499 (38%)	108
Oligopeptide ABC transporter substrate bindin	LA2_07335	E4SKB5	*L. amylovorus* GRL 111	66 kDa	SBP_bac_5	134/590 (23%)	102
Elongation factor Tu	tuf (LA2_04435	E4SI61	*L. amylovorus* GRL 111	44 kDa	N/A	169/396 (43%)	102
Cell division protein FtsI/penicillin-binding prot	LA2_04230	E4SI20	*L. amylovorus* GRL 111	79 kDa	PASTA (2); PBP_dimer; Transpept	242/720 (34%)	95
Trigger factor	tig (LA2_04440	E4SI62	*L. amylovorus* GRL 111	49 kDa	N/A	165/443 (37%)	94
50S ribosomal protein L4	rplD (LA2_0155	E4SKN8	*L. amylovorus* GRL 111	22 kDa	N/A	93/205 (45%)	93
30S ribosomal protein S7	rpsG (LA2_0153	E4SKN4	*L. amylovorus* GRL 111	18 kDa	N/A	82/156 (53%)	86
50S ribosomal protein L18	rplR (LA2_0163	E4SKY3	*L. amylovorus* GRL 111	13 kDa	N/A	43/119 (36%)	82
50S ribosomal protein L2	rplB (LA2_0156	E4SKX0	*L. amylovorus* GRL 111	30 kDa	N/A	139/278 (50%)	78
30S ribosomal protein S4	rpsD (LA2_0412	E4SNM7	*L. amylovorus* GRL 111	23 kDa	N/A	77/203 (38%)	72
N-acetylmuramidase	LA2_01030	E4SK59	*L. amylovorus* GRL 111	45 kDa	glucosamidase; SLAP	87/409 (21%)	70
Uncharacterized protein	LA2_05510	E4SIR2	*L. amylovorus* GRL 111	44 kDa	-	149/393 (38%)	68
Pyruvate kinase	LA2_04990	E4SIG2	*L. amylovorus* GRL 111	62 kDa	N/A	207/589 (35%)	68
Oligopeptide ABC transporter substrate bindin	LA2_09360	E4SMF4	*L. amylovorus* GRL 111	61 kDa	SBP_bac_5	159/545 (29%)	63
Oligopeptide ABC transporter substrate bindin	LA2_08075	E4SL67	*L. amylovorus* GRL 111	65 kDa	SBP_bac_5	126/581 (22%)	61
Penicillin binding protein	LA2_04505	E4SI75	*L. amylovorus* GRL 111	41 kDa	Beta-lactamase	131/367 (36%)	54
50S ribosomal protein L20	rplT (LA2_0859	E4SLN0	*L. amylovorus* GRL 111	14 kDa	N/A	50/118 (42%)	54
Thioredoxin reductase	LA2_03500	E4SN31	*L. amylovorus* GRL 111	34 kDa	N/A	126/309 (41%)	48
Surface protein	LA2_08860	E4SLY4	*L. amylovorus* GRL 111	35 kDa	-	40/325 (12%)	45
30S ribosomal protein S3	rpsC (LA2_0158	E4SKX3	*L. amylovorus* GRL 111	25 kDa	N/A	58/224 (26%)	45
50S ribosomal protein L5	rplE (LA2_0161	E4SKX9	*L. amylovorus* GRL 111	20 kDa	N/A	86/180 (48%)	44
Elogation factor Ts	tsf (LA2_07170	E4SK05	*L. amylovorus* GRL 111	37 kDa	N/A	91/340 (27%)	44
Aminopeptidase N	LA2_08855	E4SLY3	*L. amylovorus* GRL 111	50 kDa	peptidase_M1	112/434 (26%)	43
DNA gyrase subunit A	gyrA (LA2_0003	E4SJ61	*L. amylovorus* GRL 111	91 kDa	N/A	149/821 (18%)	43
Uncharacterized protein	LA2_00765	E4SJT0	*L. amylovorus* GRL 111	46 kDa	CAP	128/413 (31%)	43
Penicillin binding protein	LA2_00330	E4SJB8	*L. amylovorus* GRL 111	41 kDa	Beta-lactamase	92/368 (25%)	41
Elongation factor G	fusA (LA2_0154	E4SKN5	*L. amylovorus* GRL 111	77 kDa	N/A	197/697 (28%)	39
PrtP	prtP (LA2_0270	E4SM65	*L. amylovorus* GRL 111	183 kDa	DUF1034; peptidase_s8; SLAP (2)	8/1665 (0%)	39
30S ribosomal protein S13	rpsM (LA2_0167	E4SKZ1	*L. amylovorus* GRL 111	13 kDa	N/A	66/116 (57%)	36
Penicillin binding protein	LA2_05270	E4SIL6	*L. amylovorus* GRL 111	42 kDa	Beta-lactamase	111/374 (30%)	35
Enolase	eno (LA2_04675	E4SIA4	*L. amylovorus* GRL 111	47 kDa	N/A	89/428 (21%)	34
Oligopeptide ABC transporter substrate bindin	LA2_07340	E4SKB6	*L. amylovorus* GRL 111	66 kDa	SBP_bac_5	25/589 (4%)	34
Putative cell surface protein	LA2_09065	E4SM23	*L. amylovorus* GRL 111	25 kDa	YkuD	38/224 (17%)	33
50S ribosomal protein L3	rplC (LA2_0155	E4SKN7	*L. amylovorus* GRL 111	23 kDa	N/A	82/212 (39%)	32
Translation initiation factor IF-2	infB (LA2_0711	E4SJZ3	*L. amylovorus* GRL 111	97 kDa	N/A	117/867 (13%)	31

Threonine-tRNA ligase	thrS (LA2_0860	E4SLN3	*L. amylovorus* GRL 111	74 kDa	N/A	98/685 (15%)	28
Glycerol-3-phosphate ABC trnasporter	LA2_09235	E4SMC9	*L. amylovorus* GRL 111	47 kDa	-	86/434 (20%)	28
Asparagine tRNA ligase	asnC (LA2_0654	E4SJH5	*L. amylovorus* GRL 111	50 kDa	N/A	100/432 (23%)	28
50S ribosomal protein L22	rplV (LA2_0157	E4SKX2	*L. amylovorus* GRL 111	13 kDa	N/A	44/117 (38%)	28
S-layer protein	LA2_00970	E4SK47	*L. amylovorus* GRL 111	48 kDa	SLAP	19/456 (4%)	28
30S ribosomal protein S11	rpsK (LA2_0167	E4SKZ2	*L. amylovorus* GRL 111	14 kDa	N/A	43/129 (33%)	26
Fructose-bisphosphate aldolase	LA2_09055	E4SM21	*L. amylovorus* GRL 111	33 kDa	N/A	61/305 (20%)	25
DNA-directed RNA polymerase subunit alpha	rpoA (LA2_0168	E4SKZ3	*L. amylovorus* GRL 111	35 kDa	N/A	94/312 (30%)	24
30S ribosomal protein S12	rpsL (LA2_0153	E4SKN3	*L. amylovorus* GRL 111	15 kDa	N/A	31/135 (23%)	23
50S ribosomal protein L21	rplU (LA2_0752	E4SKF0	*L. amylovorus* GRL 111	11 kDa	N/A	53/103 (51%)	23
50S ribosomal protein L15	rplO (LA2_0164	E4SKY6	*L. amylovorus* GRL 111	16 kDa	N/A	62/146 (42%)	22
Putative lactocepin S-layer protein	LA2_06960	E4SJW9	*L. amylovorus* GRL 111	19 kDa	SLAP (2)	28/165 (17%)	22
Putative enterolysin A	LA2_06770	E4SJU1	*L. amylovorus* GRL 111	19 kDa	peptidase_M23	31/172 (18%)	22
Uncharacterized protein	LA2_05360	E4SIN4	*L. amylovorus* GRL 111	20 kDa	-	40/176 (23%)	20
			***L. crispatus* ATCC 33820**				
Putative uncharacterized protein	LCRIS_00224	D5H0Y6	*L. crispatus* ST1	27 kDa	SLAP	125/250 (50%)	1751
Cell separation protein	LCRIS_00225	D5H0Y7	*L. crispatus* ST1	63 kDa	FIVAR; SLAP (3)	386/589 (66%)	1482
S-layer protein	slpX (LCRIS_00508	D5H1S0	*L. crispatus* ST1	53 kDa	SLAP	340/491 (69%)	1207
Conserved protein with bacterial Ig-like domai	LCRIS_00697	D5H2A9	*L. crispatus* ST1	61 kDa	Big_3; SH3_8	350/542 (65%)	790
Conserved protein with bacterial Ig-like domai	LCRIS_00705	D5H2B7	*L. crispatus* ST1	56 kDa	Big_3	325/510 (64%)	733
S-layer protein	LCRIS_00012	D5H0C4	*L. crispatus* ST1	42 kDa	SLAP	198/368 (54%)	362
Bacteriocin helveticin-J	LCRIS_00011	D5H0C3	*L. crispatus* ST1	33 kDa	-	176/292 (60%)	351
Conserved protein	LCRIS_01523	D5GYW1	*L. crispatus* ST1	19 kDa	-	99/173 (57%)	303
S-layer protein	LCRIS_01496	D5GYT4	*L. crispatus* ST1	44 kDa	**LBA1029**	238/393 (61%)	268
Surface protein	LCRIS_01538	D5GYX6	*L. crispatus* ST1	35 kDa	-	121/325 (37%)	260
Fibronectin domain	LCRIS_00195	D5H0V7	*L. crispatus* ST1	52 kDa	fn3	280/464 (60%)	256
Lysin	LCRIS_01354	D5GYE2	*L. crispatus* ST1	44 kDa	Glyco_hydro_25; SLAP	153/412 (37%)	208
S-layer protein	LCRIS_01471	D5GYQ9	*L. crispatus* ST1	27 kDa	SLAP	145/259 (56%)	199
Penicillin-binding protein	pbpX (LCRIS_0108	D5GXM1	*L. crispatus* ST1	40 kDa	Beta-lactamase	209/356 (59%)	170
Membrane protein	LCRIS_01714	D5GZF2	*L. crispatus* ST1	30 kDa	Surface exclusion protein	172/277 (62%)	161
S-layer protein	slp1 (LCRIS_00171	D5H0T3	*L. crispatus* ST1	49 kDa	SLAP	227/466 (49%)	160
Putative uncharacterized protein	LCRIS_00591	D5H203	*L. crispatus* ST1	18 kDa	-	88/165 (53%)	146
Fibronectin-binding protein	LCRIS_01031	D5H393	*L. crispatus* ST1	52 kDa	fn3; Ig_fold	272/460 (59%)	125
Maltose ABC transporter, maltose binding prot	malE2 (LCRIS_018	D5GZY0	*L. crispatus* ST1	44 kDa	-	208/408 (51%)	117
Levansucrase	sacB (LCRIS_01741	D5GZH9	*L. crispatus* ST1	73 kDa	Glyco_hydro_68	207/695 (31%)	116
30S ribosomal protein S4	rpsD (LCRIS_0078	D5H2J4	*L. crispatus* ST1	23 kDa	N/A	160/203 (79%)	115
50S ribosomal protein L2	rplB (LCRIS_00301	D5H163	*L. crispatus* ST1	30 kDa	N/A	150/278 (54%)	108
Penicillin-binding protein	LCRIS_00057	D5H0G9	*L. crispatus* ST1	39 kDa	Beta-lactamase	237/350 (68%)	102
30S ribosomal protein S7	rpsG (LCRIS_0029	D5H157	*L. crispatus* ST1	18 kDa	N/A	109/156 (70%)	98
Lysin	LCRIS_01961	D5H049	*L. crispatus* ST1	34 kDa	Glyco_hydro_25; SLAP	144/316 (46%)	94
30S ribosomal protein S2	rpsB (LCRIS_0128	D5GY73	*L. crispatus* ST1	29 kDa	N/A	123/257 (48%)	93
Glycerol-3-phosphate ABC transporter	LCRIS_01668	D5GZA6	*L. crispatus* ST1	48 kDa	-	250/433 (58%)	92
50S ribosomal protein L4	rplD (LCRIS_00299	D5H161	*L. crispatus* ST1	22 kDa	N/A	131/205 (64%)	87
Putative uncharacterized protein	LCRIS_01297	D5GY85	*L. crispatus* ST1	42 kDa	SLAP	184/374 (49%)	86
N-acetylmuramidase	LCRIS_00183	D5H0U5	*L. crispatus* ST1	45 kDa	glucosaminidase; SLAP	196/408 (48%)	84
Conserved protein	LCRIS_01395	D5GYI3	*L. crispatus* ST1	27 kDa	-	74/249 (30%)	83
Oligopeptide ABC transporter, oligopeptide-bi	LCRIS_00200	D5H0W2	*L. crispatus* ST1	60 kDa	SBP_bac_5	258/539 (48%)	82
Glutamate tRNA ligase	gltX (LCRIS_00348	D5H1B0	*L. crispatus* ST1	58 kDa	N/A	229/499 (46%)	76
Phosphonate ABC transporter, phosphonate-b	phnD1 (LCRIS_000	D5H0C8	*L. crispatus* ST1	34 kDa	-	140/311 (45%)	76
Glycerol-3-phosphate ABC transporter	LCRIS_00593	D5H205	*L. crispatus* ST1	48 kDa	SBP_bac_1	164/434 (38%)	68
Oligopeptide ABC transporter, oligopeptide-bi	oppA2 (LCRIS_013	D5GY99	*L. crispatus* ST1	66 kDa	SBP_bac_5	46/583 (8%)	66
Lactocepin S-layer protein	LCRIS_01044	D5H3A6	*L. crispatus* ST1	19 kDa	SLAP (2)	58/166 (35%)	57
Oligopeptide ABC transporter, oligopeptide-bi	oppA1 (LCRIS_000	D5H0E5	*L. crispatus* ST1	66 kDa	SBP_bac_5	172/586 (29%)	52
Foldase protein PrsA	prsA (LCRIS_0155	D5GYY8	*L. crispatus* ST1	33 kDa	prsA	146/298 (49%)	52
30S ribosomal protein S3	rpsC (LCRIS_00304	D5H166	*L. crispatus* ST1	25 kDa	N/A	108/224 (48%)	47
30S ribosomal protein S13	rpsM (LCRIS_0032	D5H183	*L. crispatus* ST1	13 kDa	N/A	64/116 (55%)	46
Lysin	LCRIS_01140	D5GXS8	*L. crispatus* ST1	42 kDa	Glyco_hydro_25; SLAP	88/385 (23%)	45
Putative uncharacterized protein	LCRIS_01553	D5GYZ1	*L. crispatus* ST1	55 kDa	Ig/albumin binding	146/492 (30%)	41
Conserved protein	LCRIS_01449	D5GYN7	*L. crispatus* ST1	35 kDa	-	125/329 (38%)	40
Translation initiation factor IF-3	infC (LCRIS_01517	D5GYV5	*L. crispatus* ST1	17 kDa	N/A	68/149 (46%)	39
Elongation factor Tu	tufA (LCRIS_00865	D5H2S7	*L. crispatus* ST1	44 kDa	N/A	146/396 (37%)	38
Phosphonate ABC transporter, phosphonate-b	phnD2 (LCRIS_001	D5H0Q8	*L. crispatus* ST1	34 kDa	-	130/309 (42%)	38
50S ribosomal protein L5	rplE (LCRIS_00310	D5H172	*L. crispatus* ST1	20 kDa	N/A	89/180 (49%)	35
50S ribosomal protein L20	rplT (LCRIS_01515	D5GYV3	*L. crispatus* ST1	14 kDa	N/A	49/118 (42%)	33
Aggregation promoting protein	LCRIS_01883	D5GZX1	*L. crispatus* ST1	13 kDa	-	32/120 (27%)	31
Penicillin-binding protein	LCRIS_00881	D5H2U3	*L. crispatus* ST1	39 kDa	Beta-lactamase	113/337 (33%)	30
50S ribosomal protein L13	rplM (LCRIS_0032	D5H191	*L. crispatus* ST1	17 kDa	N/A	56/147 (38%)	30
Putative uncharacterized protein	LCRIS_01470	D5GYQ8	*L. crispatus* ST1	22 kDa	-	65/197 (33%)	27
30S ribosomal protein S17	rpsQ (LCRIS_0030	D5H169	*L. crispatus* ST1	11 kDa	N/A	31/88 (35%)	26
50S ribosomal protein L6	rplF (LCRIS_00313	D5H175	*L. crispatus* ST1	19 kDa	N/A	83/176 (47%)	25
Conserved protein	LCRIS_00127	D5H0N9	*L. crispatus* ST1	47 kDa	CAP	98/416 (24%)	22
30S ribosomal protein S11	rpsK (LCRIS_00322	D5H184	*L. crispatus* ST1	14 kDa	N/A	43/129 (33%)	21
50S ribosomal protein L22	rplV (LCRIS_00303	D5H165	*L. crispatus* ST1	13 kDa	N/A	33/117 (28%)	20
			***L. crispatus* NCK 953**				
Cell separation protein	LCRIS_00225	D5H0Y7	*L. crispatus* ST1	63 kDa	FIVAR; SLAP (3)	428/589 (72%)	1687
Putative uncharacterized protein	LCRIS_00224	D5H0Y6	*L. crispatus* ST1	27 kDa	SLAP	141/250 (56%)	1041
Conserved protein with bacterial Ig-like domai	LCRIS_00697	D5H2A9	*L. crispatus* ST1	61 kDa	Big_3; SH3_8	336/542 (62%)	449
Conserved protein with bacterial Ig-like domai	LCRIS_00705	D5H2B7	*L. crispatus* ST1	56 kDa	Big_3	307/510 (60%)	307
S-layer protein	LCRIS_01496	D5GYT4	*L. crispatus* ST1	44 kDa	LBA1029	228/393 (58%)	258
Putative uncharacterized protein	LCRIS_00703	D5H2B5	*L. crispatus* ST1	29 kDa	CAP	164/261 (63%)	217
S-layer protein	LCRIS_01471	D5GYQ9	*L. crispatus* ST1	27 kDa	SLAP	155/259 (60%)	213
Membrane protein	LCRIS_01714	D5GZF2	*L. crispatus* ST1	30 kDa	-	157/277 (57%)	182
SlpX	slpX (LCRIS_00508	D5H1S0	*L. crispatus* ST1	53 kDa	SLAP	210/491 (43%)	162

Putative uncharacterized protein	LCRIS_00591	D5H203	*L. crispatus* ST1	18 kDa	-	82/165 (50%)	156
Fibronectin-binding protein	LCRIS_01031	D5H393	*L. crispatus* ST1	52 kDa	fn3	317/460 (69%)	120
Glyceraldehyde-3-phosphate dehydrogenase	gapA (LCRIS_0070	D5H2B9	*L. crispatus* ST1	37 kDa	N/A	191/338 (57%)	116
Putative uncharacterized protein	LCRIS_01297	D5GY85	*L. crispatus* ST1	42 kDa	SLAP	233/374 (62%)	102
Penecillin-binding protein	LCRIS_00057	D5H0G9	*L. crispatus* ST1	39 kDa	Beta-lactamase	251/350 (72%)	97
Lysin	LCRIS_01354	D5GYE2	*L. crispatus* ST1	44 kDa	Glyco_hydro_25; SLAP	167/412 (41%)	92
Enolase	eno (LCRIS_00903	D5H2W5	*L. crispatus* ST1	47 kDa	N/A	230/428 (54%)	85
Surface protein	LCRIS_01538	D5GYX6	*L. crispatus* ST1	35 kDa	-	89/325 (27%)	78
Elongation factor Tu	tufA (LCRIS_00865	D5H2S7	*L. crispatus* ST1	44 kDa	N/A	205/396 (52%)	78
Putative uncharacterized protein	LCRIS_01612	D5GZ50	*L. crispatus* ST1	23 kDa	-	73/213 (34%)	63
Conserved protein	LCRIS_01395	D5GYI3	*L. crispatus* ST1	27 kDa	-	82/249 (33%)	53
Conserved protein	LCRIS_01523	D5GYW1	*L. crispatus* ST1	19 kDa	-	96/173 (55%)	51
Glycerol-3-phosphate ABC transporter	ugpB (LCRIS_0109	D5GXN7	*L. crispatus* ST1	47 kDa	-	167/425 (39%)	48
N-acetylmuramidase	LCRIS_00183	D5H0U5	*L. crispatus* ST1	45 kDa	Glucosaminidase; SLAP	173/408 (42%)	43
Penicillin-binding protein	pbpX (LCRIS_0108	D5GXM1	*L. crispatus* ST1	40 kDa	Beta-lactamase	138/356 (39%)	31
Penicillin-binding protein	LCRIS_00881	D5H2U3	*L. crispatus* ST1	39 kDa	Beta-lactamase	120/347 (35%)	31
			***L. crispatus* CZ6**				
Putative uncharacterized protein	LCRIS_00224	D5H0Y6	*L. crispatus* ST1	27 kDa	SLAP	141/250 (56%)	1255
Cell separation protein	LCRIS_00225	D5H0Y7	*L. crispatus* ST1	63 kDa	FIVAR; SLAP (3)	393/589 (67%)	886
SlpX	slpX (LCRIS_00508	D5H1S0	*L. crispatus* ST1	53 kDa	SLAP	306/491 (62%)	881
Conserved protein with bacterial Ig-like domai	LCRIS_00697	D5H2A9	*L. crispatus* ST1	61 kDa	Big_3; SH3_8	359/542 (66%)	801
Putative uncharacterized protein	LCRIS_00796	D5H2K8	*L. crispatus* ST1	105 kDa	SLAP	610/955 (64%)	543
Conserved protein with bacterial Ig-like domai	LCRIS_00705	D5H2B7	*L. crispatus* ST1	56 kDa	Big_3	317/510 (62%)	446
S-layer protein	LCRIS_01496	D5GYT4	*L. crispatus* ST1	44 kDa	LBA1029	245/393 (62%)	365
Conserved protein	LCRIS_01103	D5GXP1	*L. crispatus* ST1	79 kDa	-	355/697 (51%)	283
S-layer protein	LCRIS_00012	D5H0C4	*L. crispatus* ST1	42 kDa	SLAP	220/368 (60%)	220
Putative uncharacterized protein	LCRIS_00703	D5H2B5	*L. crispatus* ST1	29 kDa	CAP	161/261 (62%)	188
50S ribosomal protein L2	rplB (LCRIS_00301	D5H163	*L. crispatus* ST1	30 kDa	N/A	172/278 (62%)	167
Glycerol-3-phosphate ABC transporter	LCRIS_01668	D5GZA6	*L. crispatus* ST1	48 kDa	-	292/433 (67%)	152
Conserved protein	LCRIS_01395	D5GYI3	*L. crispatus* ST1	27 kDa	-	87/249 (35%)	146
Lipoprotein A antigen	bmpA1	D5H071	*L. crispatus* ST1	39 kDa	Bmp	195/364 (54%)	145
Putative uncharacterized protein	LCRIS_00591	D5H203	*L. crispatus* ST1	18 kDa	-	88/165 (53%)	138
50S ribosomal protein L4	rplD (LCRIS_00299	D5H161	*L. crispatus* ST1	22 kDa	N/A	131/205 (64%)	138
Fibronectin-binding protein	LCRIS_01031	D5H393	*L. crispatus* ST1	52 kDa	fn3	276/460 (60%)	134
30S ribosomal protein S4	rpsD (LCRIS_00782	D5H2J4	*L. crispatus* ST1	23 kDa	N/A	145/203 (72%)	125
S-layer protein	LCRIS_01471	D5GYQ9	*L. crispatus* ST1	27 kDa	SLAP	116/259 (45%)	123
Elongation factor Tu	tufA (LCRIS_00865	D5H2S7	*L. crispatus* ST1	44 kDa	N/A	198/396 (50%)	120
30S ribosomal protein S7	rpsG (LCRIS_00295	D5H157	*L. crispatus* ST1	18 kDa	N/A	106/156 (68%)	115
Foldase protein PrsA	prsA (LCRIS_01550	D5GYY8	*L. crispatus* ST1	33 kDa	prsA	187/298 (63%)	115
30S ribosomal protein S2	rpsB (LCRIS_01285	D5GY73	*L. crispatus* ST1	29 kDa	N/A	147/257 (57%)	103
Elongation factor G	fusA (LCRIS_00296	D5H158	*L. crispatus* ST1	77 kDa	N/A	295/697 (42%)	96
60 kDa chaperonin	groL (LCRIS_00404	D5H1G6	*L. crispatus* ST1	58 kDa	Cpn60_TCP1	360/541 (67%)	96
Putative uncharacterized protein	LCRIS_01297	D5GY85	*L. crispatus* ST1	42 kDa	SLAP	214/374 (57%)	92
30S ribosomal protein S1	rpsA (LCRIS_01018	D5H380	*L. crispatus* ST1	44 kDa	N/A	231/402 (57%)	83
Pyruvate kinase	pyk (LCRIS_01008)	D5H370	*L. crispatus* ST1	63 kDa	N/A	265/589 (45%)	77
Glutamate tRNA ligase	gltX (LCRIS_00348	D5H1B0	*L. crispatus* ST1	58 kDa	N/A	196/499 (39%)	71
Lysin	LCRIS_01961	D5H049	*L. crispatus* ST1	34 kDa	Glyco_hydro_25; SLAP	149/316 (47%)	67
Phosphonate ABC transporter, phosphonate-b	phnD2 (LCRIS_001	D5H0Q8	*L. crispatus* ST1	34 kDa	-	130/309 (42%)	67
Penicillin-binding protein	LCRIS_00881	D5H2U3	*L. crispatus* ST1	39 kDa	Beta-lactamase	130/347 (37%)	67
DNA gyrase subunit A	gyrA (LCRIS_0000	D5H0B8	*L. crispatus* ST1	92 kDa	N/A	245/826 (30%)	64
30S ribosomal protein S13	rpsM (LCRIS_0032	D5H183	*L. crispatus* ST1	13 kDa	N/A	79/116 (68%)	60
Maltose ABC transporter, maltose binding prot	malE2 (LCRIS_018	D5GZY0	*L. crispatus* ST1	44 kDa	-	225/408 (55%)	56
Putative uncharacterized protein	LCRIS_01612	D5GZ50	*L. crispatus* ST1	23 kDa	-	57/213 (27%)	54
Conserved protein	LCRIS_00127	D5H0N9	*L. crispatus* ST1	47 kDa	CAP	162/416 (39%)	53
30S ribosomal protein S17	rpsQ (LCRIS_0030	D5H169	*L. crispatus* ST1	11 kDa	N/A	31/88 (35%)	51
50S ribosomal protein L20	rplT (LCRIS_01515	D5GYV3	*L. crispatus* ST1	14 kDa	N/A	50/118 (42%)	46
30S ribosomal protein S3	rpsC (LCRIS_00304	D5H166	*L. crispatus* ST1	25 kDa	N/A	101/224 (45%)	41
S-layer protein	slp1 (LCRIS_00171	D5H0T3	*L. crispatus* ST1	49 kDa	SLAP	47/466 (10%)	40
30S ribosomal protein S9	rpsI (LCRIS_00330	D5H192	*L. crispatus* ST1	14 kDa	N/A	53/131 (40%)	38
Phosphonate ABC transporter, phosphonate-b	phnD1 (LCRIS_000	D5H0C8	*L. crispatus* ST1	34 kDa	-	108/311 (35%)	37
50S ribosomal protein L18	rplR (LCRIS_00314	D5H176	*L. crispatus* ST1	13 kDa	N/A	87/118 (74%)	37
Translation initiation factor IF-2	infB (LCRIS_01272	D5GY60	*L. crispatus* ST1	97 kDa	N/A	191/867 (22%)	35
Enolase	eno (LCRIS_00903	D5H2W5	*L. crispatus* ST1	47 kDa	N/A	120/428 (28%)	35
Sex pheromone biosynthesis protein	LCRIS_00529	D5H1U1	*L. crispatus* ST1	42 kDa	CamS	236/382 (62%)	34
50S ribosomal protein L5	rplE (LCRIS_00310	D5H172	*L. crispatus* ST1	20 kDa	N/A	89/180 (49%)	33
Glyceraldehyde-3-phosphate dehydrogenase	gapA (LCRIS_0070	D5H2B9	*L. crispatus* ST1	37 kDa	N/A	99/338 (29%)	31

Figure S1

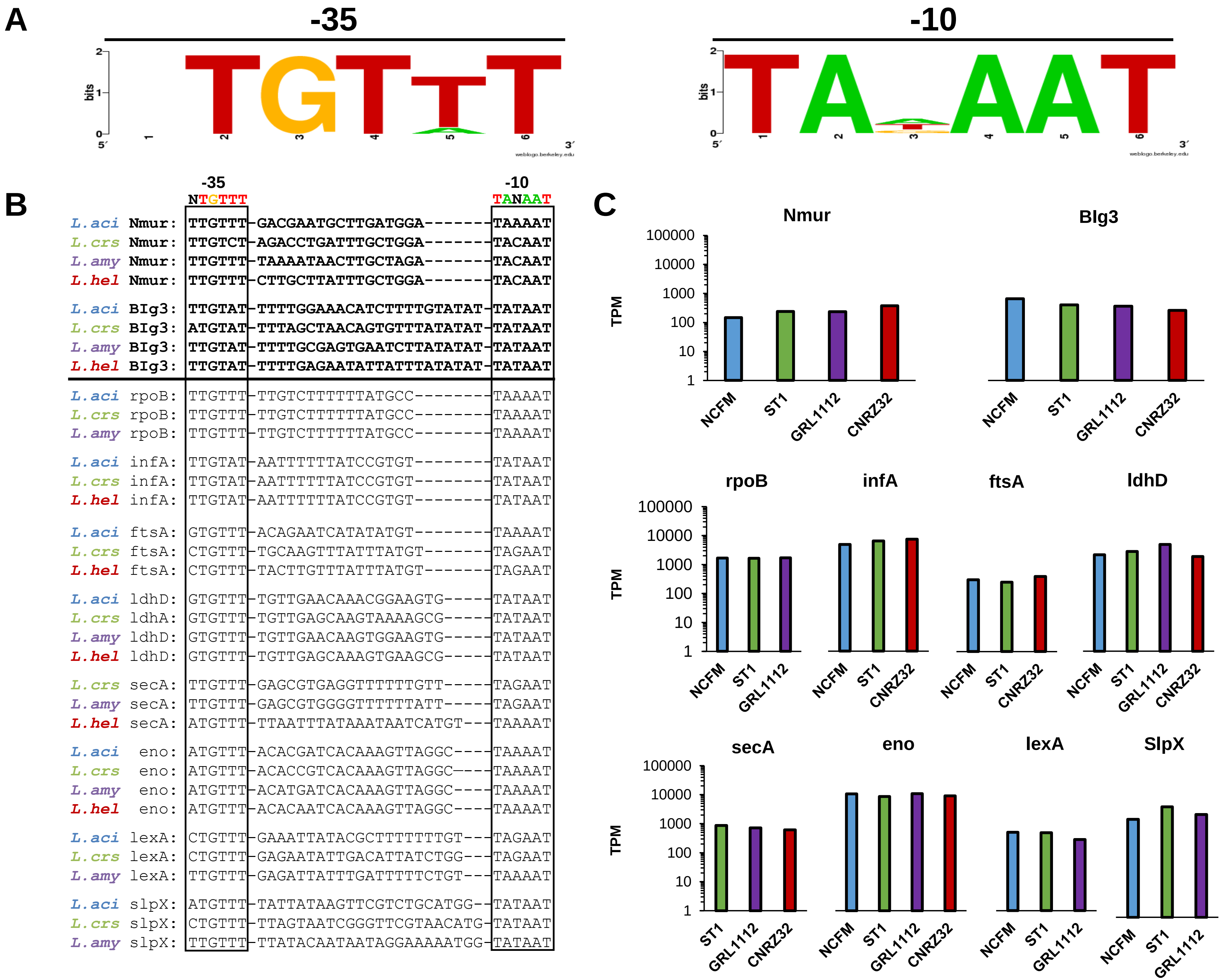
A
-35
-10
bits
weblogo.berkeley.edu
B
-35
-10
NTGTTT
TANAAT
L.aci Nmur: TTGTTT-GACGAATGCTTGATGGA-------TAAAAT
L.crs Nmur: TTGTCT-AGACCTGATTTGCTGGA-------TACAAT
L.amy Nmur: TTGTTT-TAAAATAACTTGCTAGA-------TACAAT
L.hel Nmur: TTGTTT-CTTGCTTATTTGCTGGA-------TACAAT
L.aci BIg3: TTGTAT-TTTTGGAAACATCTTTTGTATAT-TATAAT
L.crs BIg3: ATGTAT-TTTAGCTAACAGTGTTTATATAT-TATAAT
L.amy BIg3: TTGTAT-TTTTGCGAGTGAATCTTATATAT-TATAAT
L.hel BIg3: TTGTAT-TTTTGAGAATATTATTTATATAT-TATAAT
L.aci rpoB: TTGTTT-TTGTCTTTTTTATGCC--------TAAAAT
L.crs rpoB: TTGTTT-TTGTCTTTTTTATGCC--------TAAAAT
L.amy rpoB: TTGTTT-TTGTCTTTTTTATGCC--------TAAAAT
L.aci infA: TTGTAT-AATTTTTTATCCGTGT--------TATAAT
L.crs infA: TTGTAT-AATTTTTTATCCGTGT--------TATAAT
L.hel infA: TTGTAT-AATTTTTTATCCGTGT--------TATAAT
L.aci ftsA: GTGTTT-ACAGAATCATATATGT--------TAAAAT
L.crs ftsA: CTGTTT-TGCAAGTTTATTTATGT-------TAGAAT
L.hel ftsA: CTGTTT-TACTTGTTTATTTATGT-------TAGAAT
L.aci ldhD: GTGTTT-TGTTGAACAAACGGAAGTG-----TATAAT
L.crs ldhA: GTGTTT-TGTTGAGCAAGTAAAAGCG-----TATAAT
L.amy ldhD: GTGTTT-TGTTGAACAAGTGGAAGTG-----TATAAT
L.hel ldhD: GTGTTT-TGTTGAGCAAAGTGAAGCG-----TATAAT
L.crs secA: TTGTTT-GAGCGTGAGGTTTTTTGTT-----TAGAAT
L.amy secA: TTGTTT-GAGCGTGGGGTTTTTTATT-----TAGAAT
L.hel secA: ATGTTT-TTAATTTATAAATAATCATGT---TAAAAT
L.aci eno: ATGTTT-ACACGATCACAAAGTTAGGC----TAAAAT
L.crs eno: ATGTTT-ACACCGTCACAAAGTTAGGC----TAAAAT
L.amy eno: ATGTTT-ACATGATCACAAAGTTAGGC----TAAAAT
L.hel eno: ATGTTT-ACACAATCACAAAGTTAGGC----TAAAAT
L.aci lexA: CTGTTT-GAAATTATACGCTTTTTTTGT---TAGAAT
L.crs lexA: CTGTTT-GAGAATATTGACATTATCTGG---TAGAAT
L.amy lexA: TTGTTT-GAGATTATTTGATTTTTCTGT---TAAAAT
L.aci slpX: ATGTTT-TATTATAAGTTCGTCTGCATGG--TATAAT
L.crs slpX: CTGTTT-TTAGTAATCGGGTTCGTAACATG-TATAAT
L.amy slpX: TTGTTT-TTATACAATAATAGGAAAAATGG-TATAAT
C
Nmur
BIg3
rpoB
infA
ftsA
ldhD
secA
eno
lexA
SlpX
TPM
100000
10000
1000
100
10
1
NCFM
ST1
GRL1112
CNRZ32
